# Antiproliferation, 3D-multicellular spheroid and VEGFR-2 inhibitory properties of spiroindolin-2-ones with phosphonate function

**DOI:** 10.1038/s41598-025-20712-4

**Published:** 2025-10-07

**Authors:** Sara M. Hassan, Alyaa Farid, Mohamed S. Bekheit, Siva S. Panda, Benson M. Kariuki, Anwar Abdelnaser, Soad Nasr, Walid Fayad, May A. El-Manawaty, Ahmed A. F. Soliman, Adel S. Girgis

**Affiliations:** 1 Biotechnology Department, Faculty of Science, Cairo University, Giza, Egypt; 2 Department of Pesticide Chemistry, National Research Centre, Dokki, Giza, 12622 Egypt; 3 Department of Chemistry and Biochemistry, Augusta University, Augusta, GA 30912 USA; 4 Department of Biochemistry and Molecular Biology, Augusta University, Augusta, GA 30912 USA; 5 School of Chemistry, Cardiff University, Main Building, Park Place, Cardiff, CF10 3AT UK; 6 Institute of Global Health and Human Ecology, School of Sciences and Engineering, The American University in Cairo (AUC), Cairo, 11835 Egypt; 7 Drug Bioassay-Cell Culture Laboratory, Pharmacognosy Department, National Research Centre, Dokki, Giza, 12622 Egypt

**Keywords:** Spiroindolin-2-one, Cancer, Antiproliferation, 3D-spheroid, VEGFR-2, Cancer, Chemistry, Drug discovery

## Abstract

Spiroindolin-2-ones with phosphonate function 17a‒t (20 analogs, 96‒72% yield) were generated by microwave synthetic methodology using azomethine cycloaddition of the appropriate 3,5-bis(ylidene)-4-piperidone-1-phosphonate 14a‒g. Single crystal X-ray analysis of 17d confirmed the structure. Promising 2D-monolayer antiproliferation properties (MTT assay) were observed for some of the synthesized agents with no harm to normal (RPE1) cell line. Compound 17h (R = 4-ClC_6_H_4_, R′ = H; IC_50_ = 3.08 μM; 6.6- and 3.1-fold the standard drugs, 5-fluorouracil and sunitinib, respectively) is the most distinguished agent against colon/HCT116 cell line. Compound 17f (R = 4-FC_6_H_4_, R′ = Cl; IC_50_ = 5.252 μM; 3.2-fold the activity of sunitinib, the clinically approved standard drug) also has significant activity against pancreatic/PaCa2 cell line. 3D-multicellular spheroid (HCT116) testing was also performed. Notable VEGFR-2 inhibitory properties were evident for some of the synthesized analogs. Considerable activity against COX-1/-2 and TNF-α, relative to the established NSAIDs ibuprofen and indomethacin, was also detected. CAM testing evidenced the anti-VEGFR-2 observations and anti-angiogenic properties. Internal and external validated QSAR models explored the functions necessary for the antiproliferation potency. In conclusion, the designed spiroindolin-2-ones conjugated with phosphonate function can be useful for optimizing novel anti-cancer therapeutic agent(s) with anti-angiogenic (anti-VEGFR-2) mode of action after considering more needed advanced pharmacological studies.

**Supplementary Information:**

The online version contains supplementary material available at 10.1038/s41598-025-20712-4.

## Introduction

**Fig. 1:**
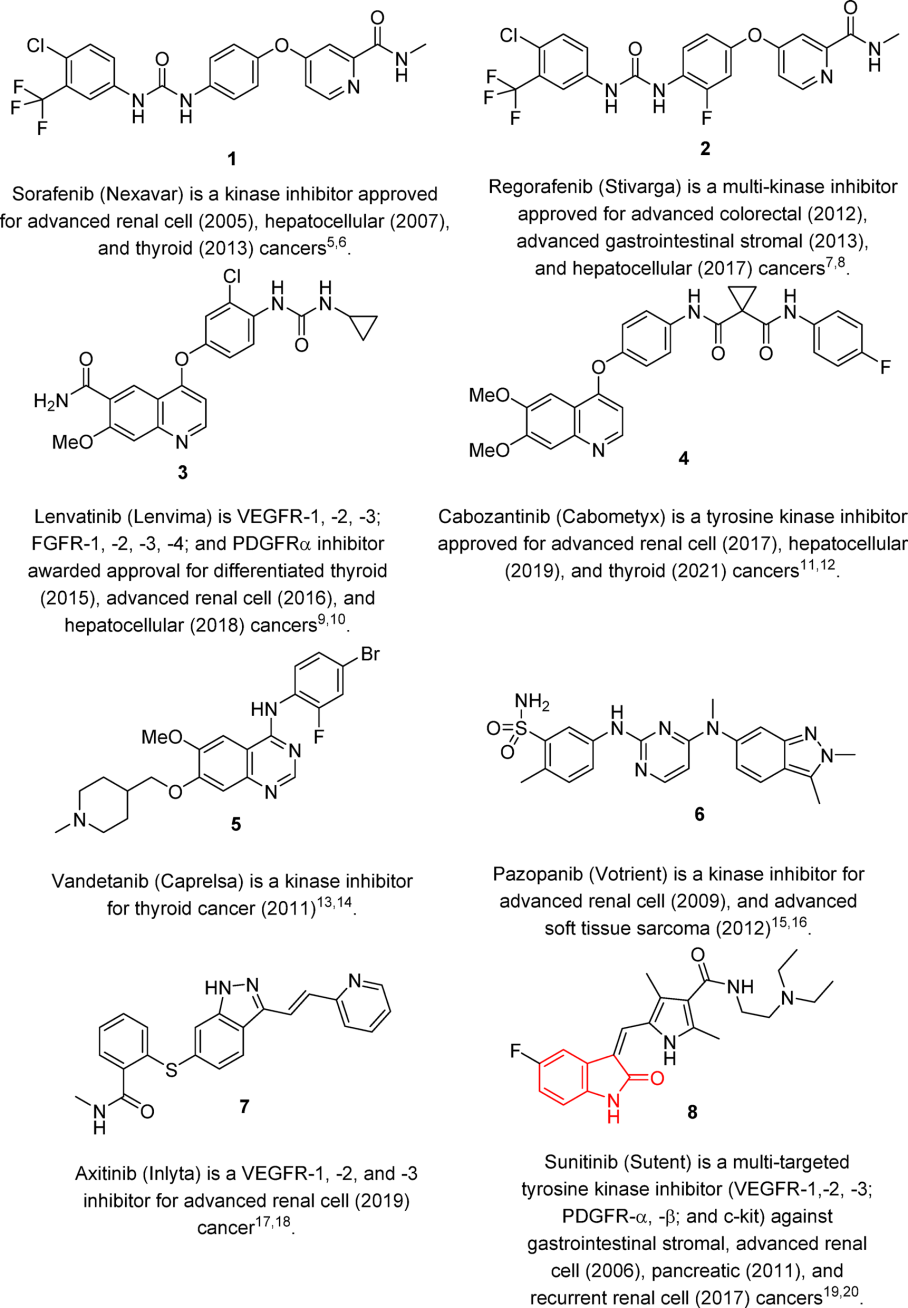
Anti-angiogenic FDA approved drugs.

Angiogenesis is a key physiological process necessary for delivering nutrients, metabolites and oxygen to endothelial cells besides the disposal of waste products, including carbon dioxide. It involves the development of new capillaries and eventually the formation of the blood vascular network from pre-existing vasculature. The process is important in a range of normal and therapeutic processes, including embryogenesis, wound healing and muscle repair, ischemic and peripheral arterial diseases and the menstrual cycle. Some pathological conditions, such as arthritis, atherosclerosis, and some solid cancerogenesis development and metastasis, are characterized by uncontrolled angiogenesis^1–4^. An approach to combating various types of cancer that has received a lot of attention in medicinal chemistry targets the design and synthesis of promising angiogenesis inhibition agents. Some anti-angiogenic small molecules have been investigated against various cancer types and awarded Food and Drug Administration (FDA) approval^5–20^ (Fig. 1).

Multiple angiogenic proteins have been identified, one family of which is the vascular endothelial growth factors (VEGF), which are tyrosine kinases hyperactivated in cancer cells. The two main categories of tyrosine kinases are receptors and non-receptors. The receptor type is trans-membrane (extracellular or intracellular) whereas the non-receptor type is only intracellular. VEGF sub-types include VEGF-A, -B, -C, -D, and –E, but only the first four subtypes are naturally found in the human genome. They are generally categorized according to their binding into receptors VEGFR-1, -2, and -3^21–23^. Anti-VEGFR-2 agents have been reported to be promising hits and/or leads against many solid cancer types (lung, breast, colon, renal, skin, etc.)^21,24–26^.

Indolin-2-one is a heterocyclic motif that has progressively gained interest in the last decades due to the biological properties associated with the developed new analogs^27^. Sunitinib (Sutent) 8 is an analog that has famously gained FDA approval for clinical use against gastrointestinal stromal, advanced renal cell (2006), pancreatic (2011), and recurrent renal cell (2017) cancers with a multi-targeted tyrosine kinase (VEGFR-1,-2, -3; PDGFR-α, -β; and c-kit) inhibitory effect^19,20,28^ (Fig. 1).

**Fig. 2:**
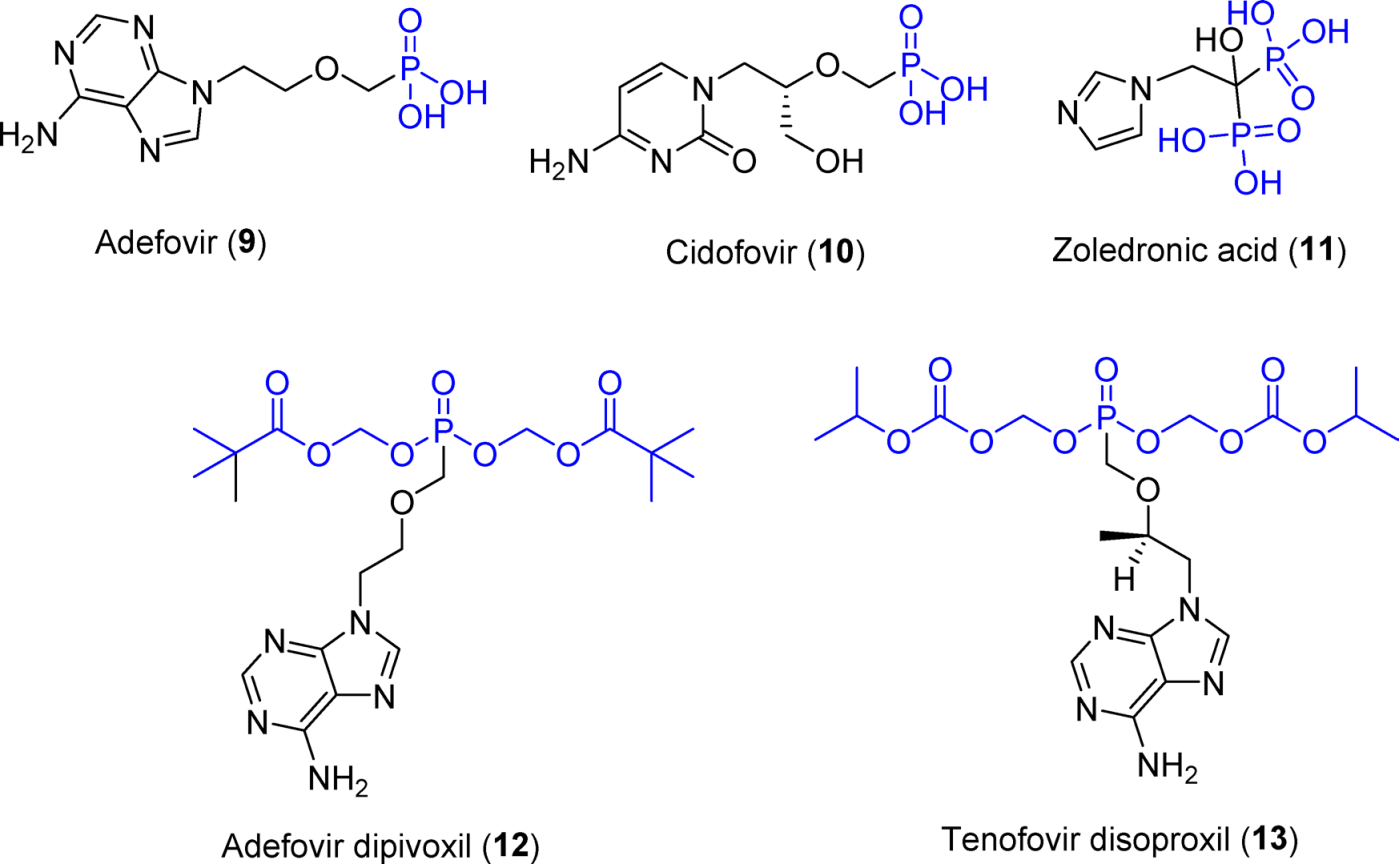
Drugs/pro-drugs with phosphonate group.

The study adopts synthesis of spiroindolin-2-one analogs collaborating with phosphonate group. The phosphonate group is isosteric to the phosphate group. Some phosphonate compounds are well known drugs, including adefovir 9^29^, cidofovir 10^30^ (antiviral), and zoledronic acid 11^31^ (anti-osteoporotic), or pro-drugs such as adefovir dipivoxil 12 (a pro-drug of adefovir)^32^ and tenofovir disoproxil 13 (a pro-drug of tenofovir phosphonate^33^, which is used to treat hepatitis B). Phosphate esters are highly successful in enhancing the delivery of drug and/or drug candidates that are poorly soluble in water following oral administration^34,35^. Thus, judicious modification of the target agent can elevate its potency^36^ (Fig. 2).

The current study is directed towards synthesizing spiro-indolin-2-one-containing compounds and investigating their antiproliferation properties against a variety of human cancer cell lines with determination of their mode of action as anti-angiogenic agents against VEGFR-2. Interest in this subject is attributed to the antitumor properties associated with the structure of the targeted scaffold and continuation of our efforts in this drug discovery program directed towards exploring antiproliferation properties of spiroindolin-2-one containing compounds^37–41^. The reason for addition of the phosphonate group into the targeted analogs is due to the associated hydrophobic properties that may positively impact the overall pharmacokinetic properties of the designed agents. The pro-drug design approach is a viable option to achieve enhanced bioavailability of a drug candidate characterized by low hydrophilicity and poor cell membrane permeability. Functionalizing the drug candidate with a moiety like carboxylic, carbonyl, carboxamide, or phosphate/phosphonate groups is one of the most effective pro-drug design approaches for a wide range of drug molecules^42^. Incorporation of an ionizable group, such as phosphate, amino acid and sugar moieties, in the promising agents/drug candidates results in enhancement in aqueous solubility, which helps to achieve the desired bioavailability of the pharmaceutically active potential molecule(s)^34^. This justifies the collaboration of the phosphonate group with the targeted spiro-heterocyclic scaffold in this work. Some of the targeted analogs have been investigated by our group, revealing promising antiviral properties with potential M^pro^-SARS-CoV-2 inhibitory effect^43^. Clinical trials supported the ability of treating colon cancer patient with viral infection by either antiviral drug alone or in combination with anticancer drug(s)^44^ motivated our previous work for investigating their antiviral properties. However, the current work moved towards antiproliferation properties investigation of the synthesized spiroindolin-2-one analogs conjugated with phosphonate group against a variety of cancer types and focusing on studying their mode of action against VEGFR-2 motivated by the reported anti-cancer properties of the adopted chemical scaffold^37–41^.

## Results and discussion

### Design of the targeted agents

**Fig. 3:**
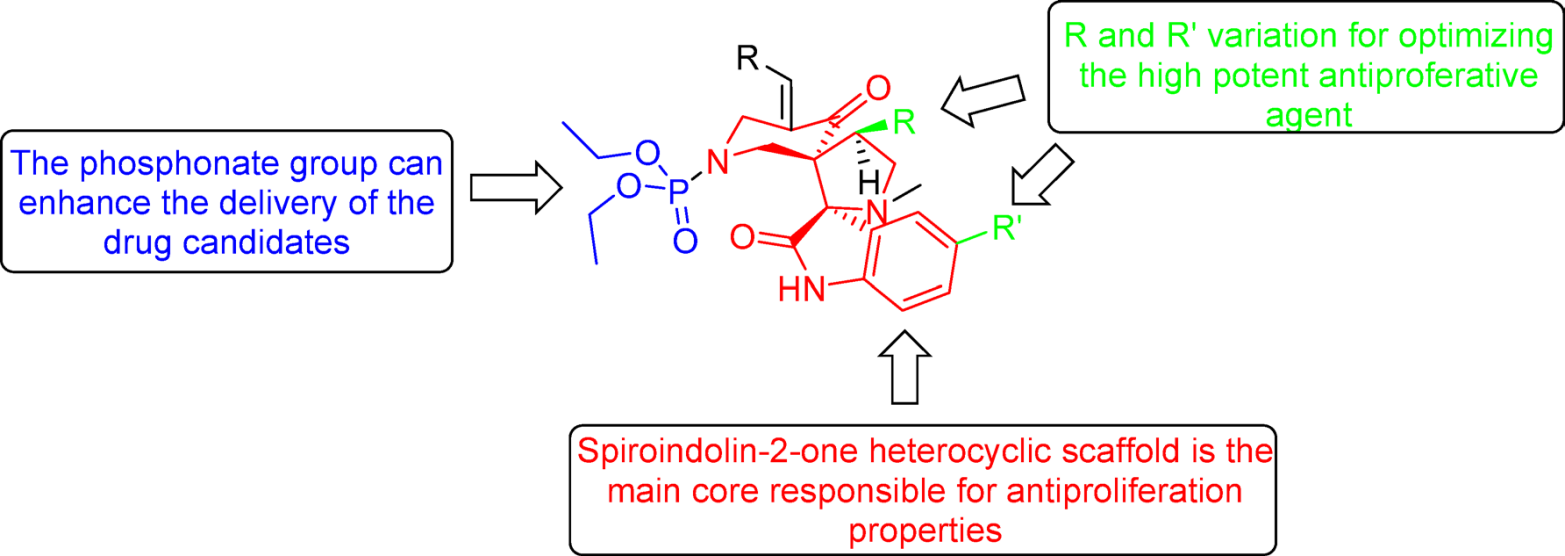
Design of the targeted agents.

The current study is directed towards development of spiroindolin-2-one analogs conjugated with a phosphonate group of potential antiproliferation properties against a variety of cancer cell lines. The main heterocyclic core (spiroindolin-2-one) is responsible for the antiproliferation properties of the targeted agents^37–41^. The conjugated phosphonate group can assist in the delivery of the drug candidates^34–36^. Meanwhile, variation of R and R′ is adopted for optimizing a high effective/potent agent(s) (Fig. 3).

### Chemical synthesis

**Fig. 4:**
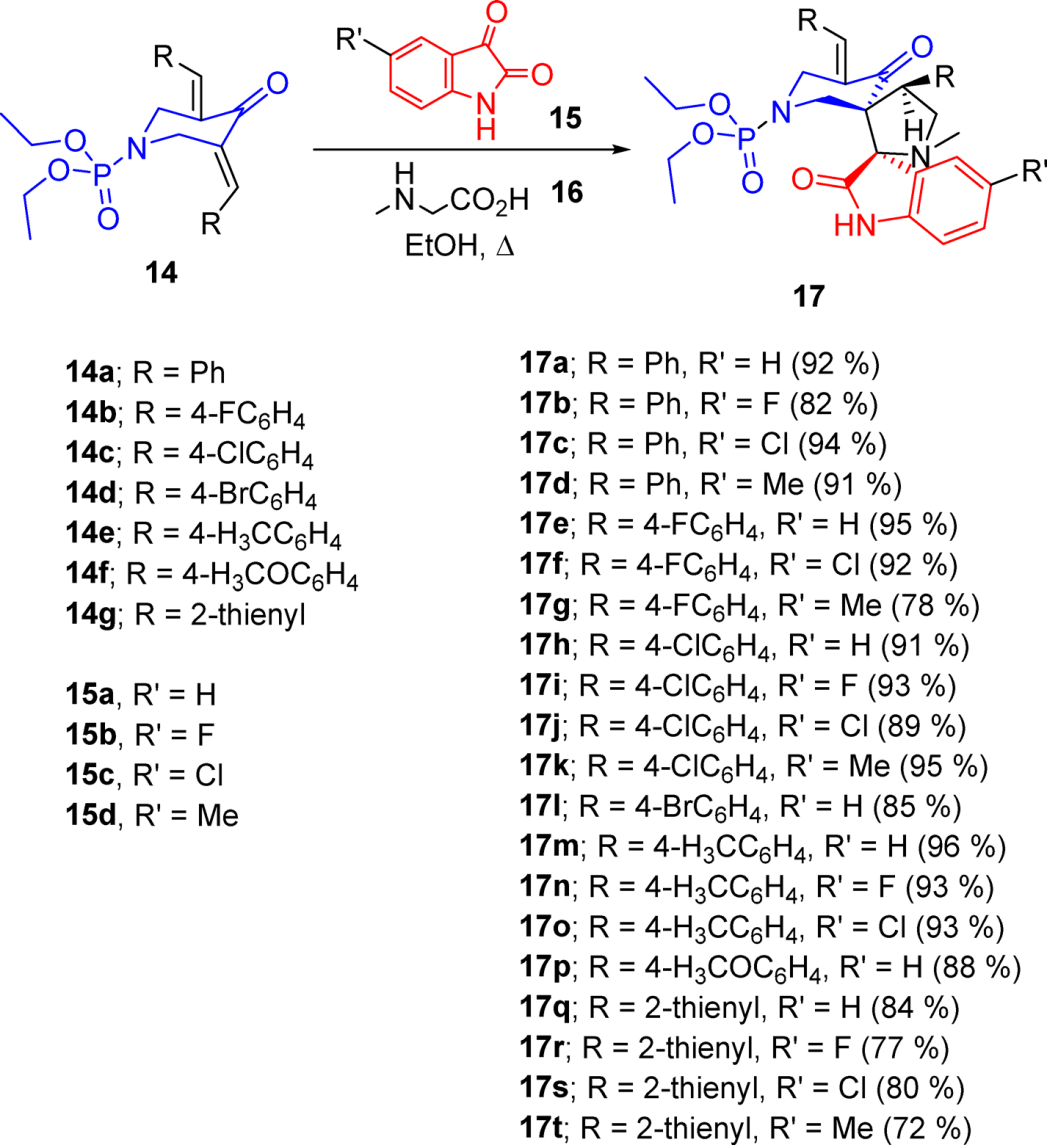
Synthetic route towards the targeted agents 17a‒t.

Microwave-assisted dipolar cycloaddition towards the targeted spiroindolin-2-ones bearing phosphonate group 17a‒t were generated in high yield (96‒72%) through reaction of non-stabilized azomethine ylides (produced during the interaction “in-situ” from isatins 15a‒d and sarcosine 16) with the activated olefinic bond of piperidines 14a‒g^43^ (Fig. 4). ^1^H-NMR showed the methylene groups of piperidone (H_2_C-2′′ and H_2_C-6′′) and pyrrolidine (H_2_C-5′) heterocycles as diastereotopic protons. The multiple signals of the methyl and methylene carbons of phosphonate groups are due to the effect of phosphorus atom. Regioselectivity of the reaction seems a general characteristic function associated with the azomethine cycloaddition under the applied experimental conditions^43^. Single crystal X-ray characterization of 17d evidenced the chemical structure.

### Crystal structure of 17d

**Fig. 5:**
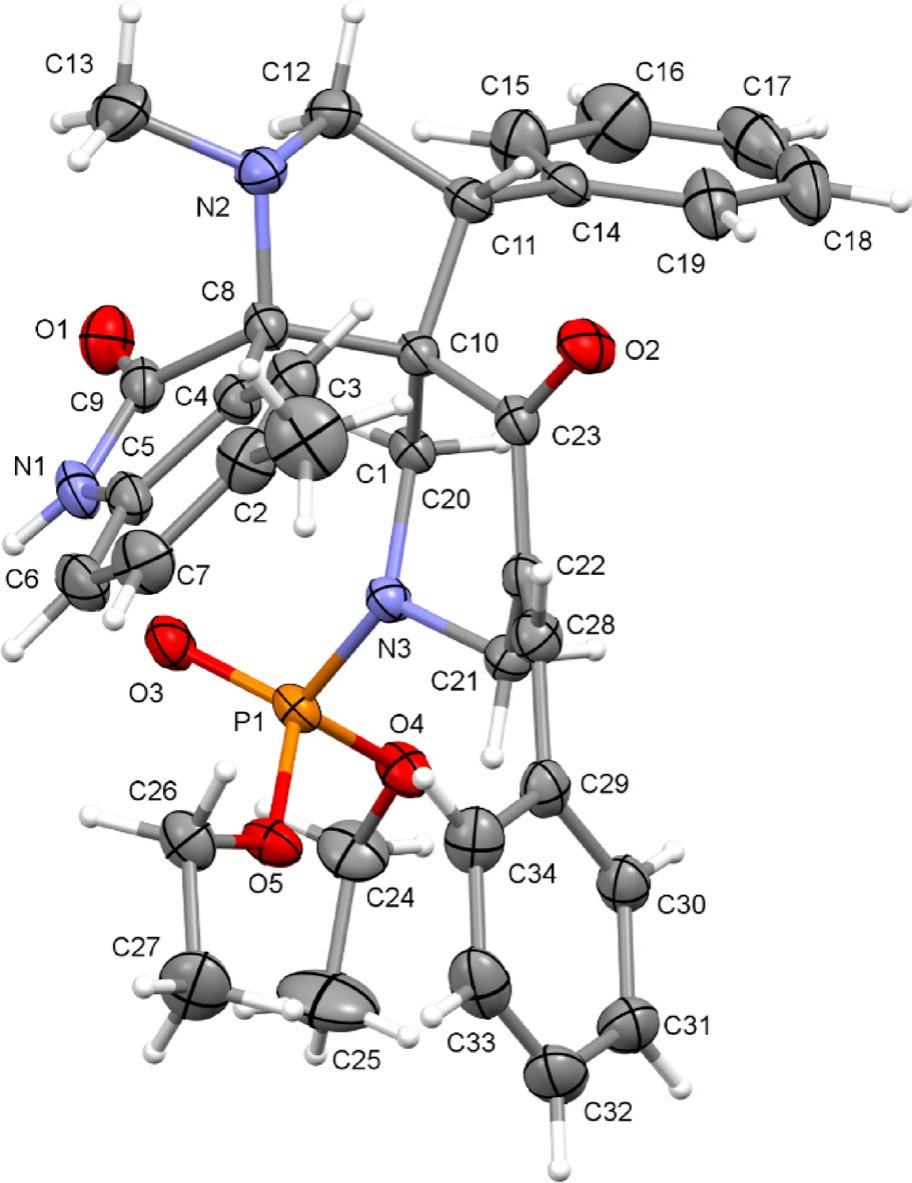
An Ortep representation of the molecule from the crystal structure of compound 17d.

The crystal structure is triclinic, P-1 (Supplementary Table S1), and the asymmetric unit contains one molecule of 17d and half a molecule of methanol (solvent of crystallization) which is disordered on an inversion symmetry center. At the center of the molecule of 17d (Fig. 5) is a 2,7-diazaspiro[4.5]decan-10-one ring system comprising a pyrrolidine ring (PYR: C8 – C12, N2) and a piperidin-4-one (PIP: C10, C20-C23, N3, O2) group. The pyrrolidine ring is in chair conformation with the methylene group as the flap and the piperidin-4-one ring is in an unsymmetrical chair conformation. The 1,3-dihydro-2*H*-indol-2-one group (DHI: C2 – C9, N1, O1) is planar and is connected to the pyrrolidine ring through the shared atom C8. The linked PYR, PIP and DHI groups form a core with limited flexibility whereas phenyl (C14‒C19), methylbenzene (C28–C34) and diethylphosphonate (C24‒C27, O3 O5, P1) groups introduce more conformational flexibility to the molecule. In the crystal, pairs of molecules of 17d are linked by two N–H…O across an inversion center. The N–H group is the donor, and the acceptor is the diethyl phosphonate oxygen, with a N1-H1…O3 angle of 164.7° and N1…O3 distance of 2.828(2) Å. An O–H…O hydrogen bond is also observed between the methanol molecule and the piperidin-4-one oxygen, with a O6-H6A…O2 angle of 171.0° and O6…O2 distance of 2.926(7) Å.

### Biological studies

#### 2D-Monolayer antiproliferation properties

The MTT [3-(4,5-dimethylthiazol-2-yl)-2,5-diphenyl-tetrazolium bromide] to purple formazan standard technique was adopted for studying the 2D-monolayer antiproliferation properties of the synthesized analogs 17a‒t against a set of cancer cell lines (HCT116/colon, PaCa2/pancreatic, MCF7/breast, and A549/lung) in addition to normal (RPE1 “retinal pigment epithelial”) cell for determining the safety index/behavior^30,45^. The properties were compared with clinically approved anticancer drugs including sunitinib (gastrointestinal, renal, and pancreatic cancer)^19,20^, 5-fluorouracil (colon, rectum, breast and skin)^46,47^, and doxorubicin (broad spectrum anti-cancer drug against many cancer types, including breast, lung, gastric, and ovarian)^48^. The results are presented in Table 1 and Supplementary Figs. S25‒S29.

**Table 1:** Antiproliferation properties of the tested compounds.

Compd	IC_50_, μM ± SEM (SI^a^)				
	HCT116	PaCa2	MCF7	A549	RPE1
17a	7.971 ± 0.338 (2.1)	12.920 ± 0.559 (1.3)	5.857 ± 0.259 (2.9)	14.450 ± 0.259 (1.2)	16.790 ± 0.516
17b	7.409 ± 0.228 (3.1)	12.230 ± 0.297 (1.9)	5.826 ± 0.213 (4.0)	10.990 ± 0.135 (2.1)	23.100 ± 0.884
17c	6.317 ± 0.132 (2.2)	9.148 ± 0.154 (1.5)	5.269 ± 0.172 (2.6)	12.540 ± 0.205 (1.1)	13.750 ± 0.423
17d	9.278 ± 0.084 (1.9)	15.570 ± 0.329 (1.2)	5.782 ± 0.130 (3.1)	14.590 ± 0.270 (1.2)	17.900 ± 0.945
17e	6.989 ± 0.101 (2.4)	9.479 ± 0.278 (1.8)	5.640 ± 0.273 (2.9)	7.727 ± 0.155 (2.1)	16.600 ± 0.907
17f	3.716 ± 0.099 (10.7)	5.252 ± 0.224 (7.6)	5.289 ± 0.175 (7.5)	> 50.000 ± 2.111 (< 0.8)	39.890 ± 1.162
17g	5.006 ± 0.364 (3.8)	6.092 ± 0.132 (3.1)	5.426 ± 0.152 (3.5)	8.149 ± 0.277 (2.3)	19.040 ± 0.647
17h	3.080 ± 0.165 (2.6)	6.354 ± 0.170 (1.3)	4.067 ± 0.054 (2.0)	4.149 ± 0.288 (2.0)	8.147 ± 0.675
17i	4.142 ± 0.042 (> 12.1)	13.450 ± 0.898 (> 3.7)	4.808 ± 0.148 (> 10.4)	> 50.000 ± 1.431 (–)	> 50.000 ± 1.173
17j	3.487 ± 0.033 (> 14.3)	6.930 ± 0.337 (> 7.2)	3.874 ± 0.102 (> 12.9)	6.059 ± 0.157 (> 8.3)	> 50.000 ± 1.491
17k	3.281 ± 0.022 (2.5)	6.659 ± 0.095 (1.2)	4.978 ± 0.095 (1.6)	6.467 ± 0.385 (1.3)	8.150 ± 0.507
17l	4.162 ± 0.129 (1.9)	6.151 ± 0.413 (1.3)	4.852 ± 0.082 (1.6)	7.281 ± 0.316 (1.1)	7.848 ± 0.284
17m	3.438 ± 0.083 (2.8)	5.605 ± 0.063 (1.7)	4.210 ± 0.085 (2.3)	8.182 ± 0.435 (1.2)	9.569 ± 0.537
17n	5.095 ± 0.191 (> 9.8)	15.340 ± 0.911 (> 3.3)	4.368 ± 0.156 (> 11.5)	> 50.000 ± 1.752 (–)	> 50.000 ± 1.739
17o	3.756 ± 0.064 (> 13.3)	6.273 ± 0.148 (> 8.0)	4.666 ± 0.086 (> 10.7)	36.000 ± 1.150 (> 1.3)	> 50.000 ± 1.541
17p	5.512 ± 0.138 (2.6)	7.316 ± 0.226 (1.9)	5.430 ± 0.140 (2.6)	12.990 ± 0.284 (1.1)	14.200 ± 0.638
17q	11.760 ± 0.400 (2.3)	13.360 ± 0.325 (2.0)	9.134 ± 0.314 (3.0)	24.510 ± 0.204 (1.1)	27.200 ± 0.813
17r	7.670 ± 0.379 (2.2)	8.292 ± 0.497 (2.1)	6.224 ± 0.082 (2.7)	14.180 ± 0.135 (1.2)	17.020 ± 0.557
17s	5.416 ± 0.288 (1.9)	6.134 ± 0.131 (1.7)	4.639 ± 0.068 (2.2)	8.096 ± 0.091 (1.3)	10.200 ± 0.545
17t	7.342 ± 0.161 (3.5)	9.474 ± 0.097 (2.7)	6.447 ± 0.111 (3.9)	14.120 ± 0.201 (1.8)	25.350 ± 0.951
Sunitinib^b^	9.67	16.91	3.97	–	–
5-Fluorouracil^b,c^	20.43	–	3.15	–	–
Doxorubicin^c^	–	–	–	5.93	–

^a^SI = Selectivity index (IC_50_ of RPE1 “normal cell”/IC_50_ of cancer cell), ^b^Reference 30, ^c^Reference 45.

##### HCT116

Colon cancer is one of the most prevalent malignant tumors of the digestive system worldwide and is ranked second of all cancers based on mortality rate. Environmental, dietary, and lifestyle factors are connected to its incidence. Although many chemotherapeutics have been developed and some have been awarded clinical approval, the limited efficacy especially towards the advanced, recurrent, and metastatic conditions as well as the associated side effects drives the need for newer agents^49,50^.

All the agents synthesized in this investigation exhibited higher anti-HCT116 efficacies than the clinically approved drug 5-fluorouracil (IC_50_ = 20.43 μM). Compound 17h (R = 4-ClC_6_H_4_, R′ = H) is the most promising of the agents with potency IC_50_ = 3.08 μM (6.6- and 3.1-folds of the standard drugs 5-fluorouracil and sunitinib, respectively). Compounds 17f (R = 4-FC_6_H_4_, R′ = Cl), 17j (R = 4-ClC_6_H_4_, R′ = Cl), 17k (R = 4-ClC_6_H_4_, R′ = Me) 17m (R = 4-H_3_CC_6_H_4_, R′ = H) and 17o (R = 4-H_3_CC_6_H_4_, R′ = Cl) also had high efficacies (IC_50_ = 3.281‒3.756 μM).

**Fig. 6:**
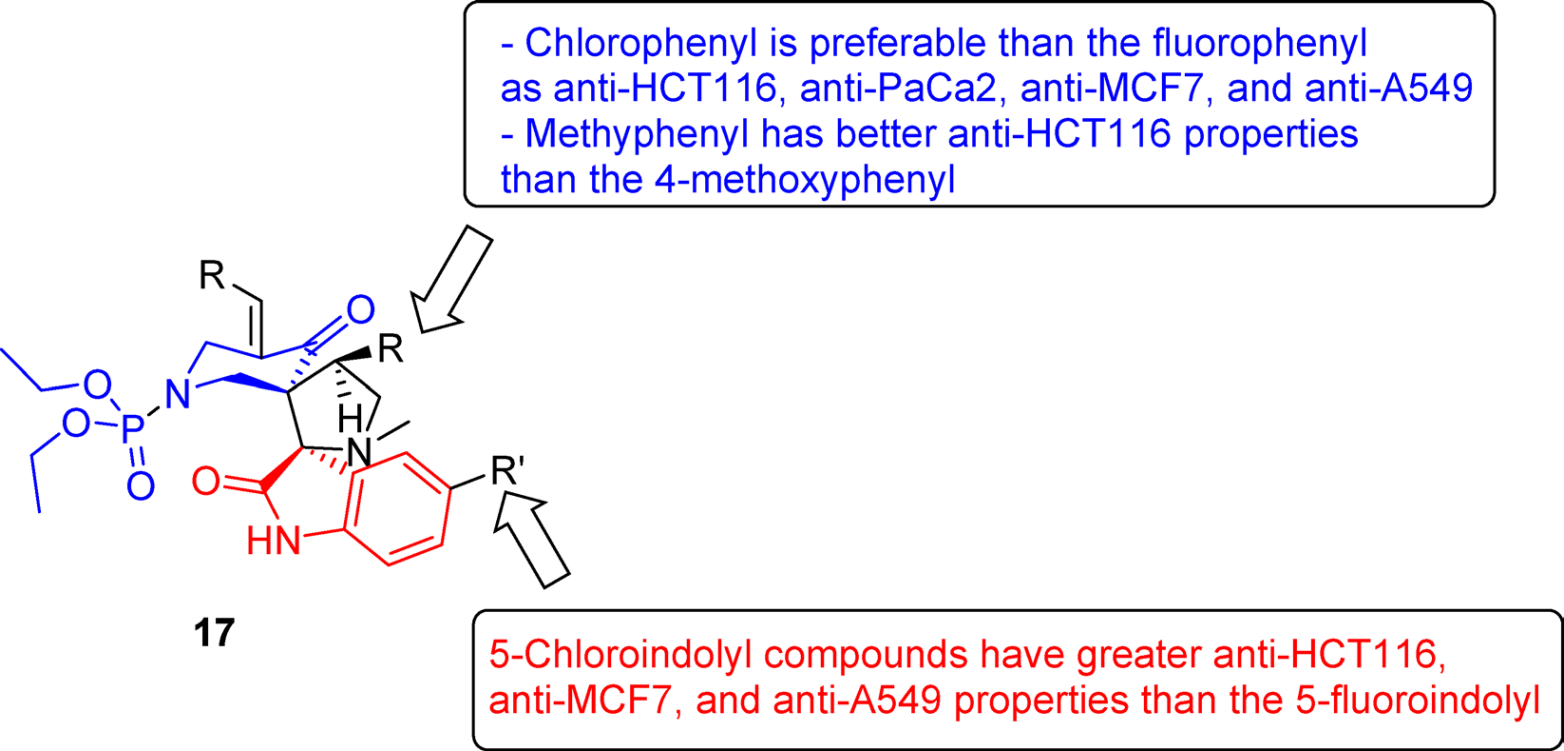
Summary of the most important items revealed through analysis of SARs due to antiproliferation properties of 17a‒t against the tested cancer cell lines.

Based on the observed anti-HCT116 properties, some SARs (structure–activity relationships) can be deduced. The 4-chlorophenyl-containing analogs show enhanced anti-HCT116 properties relative to the other halogen-containing compounds as noted in 17h/17e/17l (IC_50_ = 3.080/6.989/4.162 μM, respectively), 17j/17f (IC_50_ = 3.487/3.716 μM, respectively), and 17k/17g (IC_50_ = 3.281/5.006 μM, respectively). Similarly, the 4-methy-containing analog has better anti-HCT116 properties than the 4-methoxy-containing compound as shown by 17m/17p (IC_50_ = 3.438/5.512 μM, respectively). The 5-chloroindolyl-containing compounds have greater anti-HCT116 properties than the 5-fluoroindolyl analogs as noted in pairs 17c/17b (IC_50_ = 6.317/7.409 μM, respectively), 17j/17i (IC_50_ = 3.487/4.142 μM, respectively), 17o/17n (IC_50_ = 3.756/5.095 μM, respectively), and 17s/17r (IC_50_ = 5.416/7.670 μM, respectively) (Fig. 6).

##### PaCa2

Pancreatic cancer is one of the most aggressive forms of the disease (often referred to as the king of cancers) with a low five-year survival rate (≈10%) and high recurrence rates^51^. Patients diagnosed with pancreatic cancer are usually 40‒85 years old^52^. Difficulties in early diagnosis, due to non-specific symptoms, in addition to the ability for migration and metastasis account for the survival rates^53^.

All agents 17a‒t show efficacy against PaCa2 cell line with higher potencies or close to that of sunitinib (clinically approved drug against pancreatic cancer, IC_50_ = 16.91 μM). Compound 17f (R = 4-FC_6_H_4_, R′ = Cl) is the most promising anti-PaCa2 agent observed (IC_50_ = 5.252 μM, i.e. 3.2-fold that of the standard drug). Notable efficacy is also observed for compound 17m (R = 4-H_3_CC_6_H_4_, R′ = H; IC_50_ = 5.605 μM). Additionally, compounds 17g (R = 4-FC_6_H_4_, R′ = Me), 17h (R = 4-ClC_6_H_4_, R′ = H), 17l (R = 4-BrC_6_H_4_, R′ = H), 17o (R = 4-H_3_CC_6_H_4_, R′ = Cl), and 17s (R = 2-thienyl, R′ = Cl), exhibit considerable potencies (IC_50_ = 6.092‒6.354 μM).

SAR based on the observed anti-PaCa2 properties indicate that the 5-chloroindolyl bearing compounds have higher anti-PaCa2 activity than that of the 5-fluoroindolyl-containing analogs as seen in pairs 17c/17b (IC_50_ = 9.148/12.230 μM, respectively), 17j/17i (IC_50_ = 6.930/13.450 μM, respectively), 17o/17n (IC_50_ = 6.273/15.340 μM, respectively), and 17s/17r (IC_50_ = 6.134/8.292 μM, respectively) (Fig. 6).

##### MCF7

Breast cancer is the second highest cause of malignant mortality in women. According to the National Breast Cancer Coalition, one woman dies due to this disease every 13 min globally. It is a heterogeneous disease that is difficult to diagnose, especially in the early stages. Although several techniques have been developed and are clinically used (including, X-ray, ultrasound, computed tomography (CT), and magnetic resonance imaging), low sensitivity and specificity is a challenge impacting these tools. Patients with less than 1 cm^3^ (about 10^9^ cancer cells) usually return negative imaging results. This is a serious challenge, not only for the diagnosis but also for the treatment, that needs highly effective agents with minimal drawbacks^54,55^.

Compound 17j (R = 4-ClC_6_H_4_, R′ = Cl) is the most promising agent synthesized (IC_50_ = 3.874 μM) with anti-MCF7 potency close to that of 5-fluorouracil (a clinically usable drug agent with IC_50_ = 3.15 μM). Compounds 17h (R = 4-ClC_6_H_4_, R′ = H), 17m (R = 4-H_3_CC_6_H_4_, R′ = H), and 17n (R = 4-H_3_CC_6_H_4_, R′ = F) also exhibit anti-MCF7 properties (IC_50_ = 4.067‒4.368 μM) close to that of 5-fluorouracil. Based on the anti-MCF7 properties of the synthesized compound 17a‒t, some SARs were noted that seem similar to those for anti-HCT116 agents. The 4-chlorophenyl-containing compounds are superior among all the halogenated phenyl-containing analogs (fluoro or bromo derivatives) as shown by compounds 17h/17e/17l (IC_50_ = 4.067/5.640/4.852 μM, respectively), 17j/17f (IC_50_ = 3.874/5.289 μM, respectively), and 17k/17g (IC_50_ = 4.978/5.426 μM, respectively). The 5-chloroindolyl-containing compounds have more enhanced anti-MCF7 properties than the 5-fluoroindolyl analogs (compound 17o is an exception), as noted for compounds 17c/17b (IC_50_ = 5.269/5.826 μM, respectively), 17j/17i (IC_50_ = 3.874/4.808 μM, respectively), and 17s/17r (IC_50_ = 4.639/6.224 μM, respectively) (Fig. 6).

##### A549

Lung cancer is one of the most prevalent and deadly types of cancer (with a low five-year survival rate of 15%), due to difficulties in early detection as it is usually asymptomatic^56^. Metastasis and recurrence of this disease are major factors contributing to the low survival rate. Tobacco smoking, unhealthy environmental conditions and genetic elements are linked to lung cancer^57^. Two main types of lung cancers have been identified, non-small cell cancer which is the most common (85%), and small cell lung cancer (15%), which is more aggressive^58^.

Compound 17h (R = 4-ClC_6_H_4_, R′ = H; IC_50_ = 4.149 μM) is the most effective anti-A549 analog, surpassing doxorubicin (IC_50_ = 5.93 μM). Additionally, compounds 17j (R = 4-ClC_6_H_4_, R′ = Cl), and 17k (R = 4-ClC_6_H_4_, R′ = Me) also show high efficacies (IC_50_ = 6.059, 6.467 μM, respectively).

SARs derived from the observed anti-A549 properties mirror those noted for other cancer cell lines (HCT116, MCF7). The 4-chlorophenyl group is preferable over the other 4-halogenated phenyl residues as shown by compounds 17h/17e/17l (IC_50_ = 4.149/7.727/7.281 μM, respectively), 17j/17f (IC_50_ = 6.059/ > 50.000 μM, respectively), and 17k/17g (IC_50_ = 6.467/8.149 μM, respectively). 5-Chloroindolyl-containing compounds have more enhanced anti-A549 properties than the 5-fluoroindolyl-containing analogs (17b is an exception), as illustrated by compounds 17j/17i (IC_50_ = 6.059/ > 50.000 μM, respectively), 17o/17n (IC_50_ = 36.000/ > 50.000 μM, respectively), and 17s/17r (IC_50_ = 8.096/14.180 μM, respectively) (Fig. 6).

Some of the synthesized agents showed a high selectivity index (SI) due to IC_50_ values against RPE1 (healthy/non-cancer) compared to the tested cancer cell line(s) including 17f, 17i, 17j, 17n, and 17o. However, these preliminary observations are only indicative based on cell line testing and the most important data come from further biological studies, such as animal modeling.

### 3D-multicellular spheroid

The 3D-multicellular spheroid model is an important technique bridging and/or linking the biological properties of a specific agent, shown to have a promising antiproliferation effect in the in vitro 2D-single layer culture, with the in vivo animal model. This is due to the physiological environment available in the 3D-spheroid that allows cell–cell interactions, due to their morphological capacity, with similarity to that of cancer tissue. Oxygen and nutrient transmission in the 3D-multilayer model is highly similar to that of the in vivo tissue. In contrast, cells in the 2D-single layer technique possess sufficient nutrients and oxygen allowing high proliferation rates relative to the 3D-technique. Additionally, drug penetration to the inner cell layers of the spheroid is more difficult thus allowing high survival rates for the inner layers than the outer ones. This makes the 3D-spheroid technique a close mimic platform to in vivo modeling^59,60^.

**Fig. 7:**
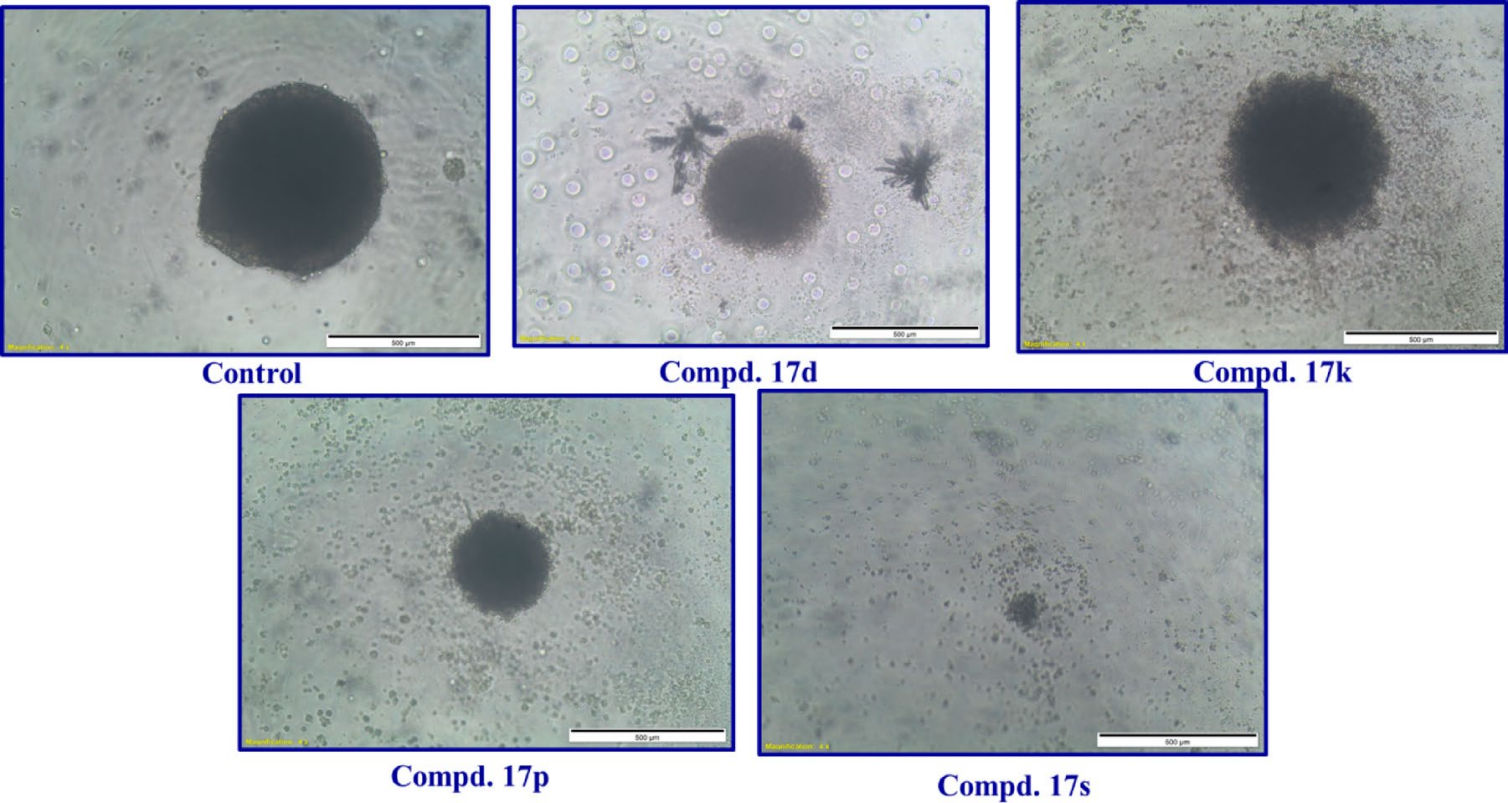
HCT116 3D-multilayer spheroids contained proliferating control and treated with 50 μM of compounds 17d, 17k, 17p, and 17s.

The synthesized agents were subjected to 3D-multilayer HCT116 cancer spheroid utilizing the standard methodology^61,62^. From the observed results it is noted that, some of the synthesized agents reveal promising activity at 50 μM (Table 2, Fig. 7). Compound 17s (R = 2-thienyl, R′ = Cl) is the most promising agent noted revealing antiproliferation properties of 77.9% relative to the control experiment. Compounds 17p (R = 4-H_3_COC_6_H_4_, R′ = H), and 17d (R = Ph, R′ = Me) show slightly lower efficacies (%CT = 68.3, and 64.1, respectively) and compound 17k (R = 4-ClC_6_H_4_, R′ = Me) has even milder properties (%CT = 53.1).

**Table 2:** The percentage cytotoxicity (%CT) of the effective agents against HCT116 3D-spheroid at 50 μM.

Compd	%CT (± SEM)
17d	64.1 ± 7.0
17k	53.1 ± 2.2
17p	68.3 ± 0.6
17s	77.9 ± 1.2

### Biochemical studies

#### VEGFR-2 inhibitory properties

**Fig. 8:**
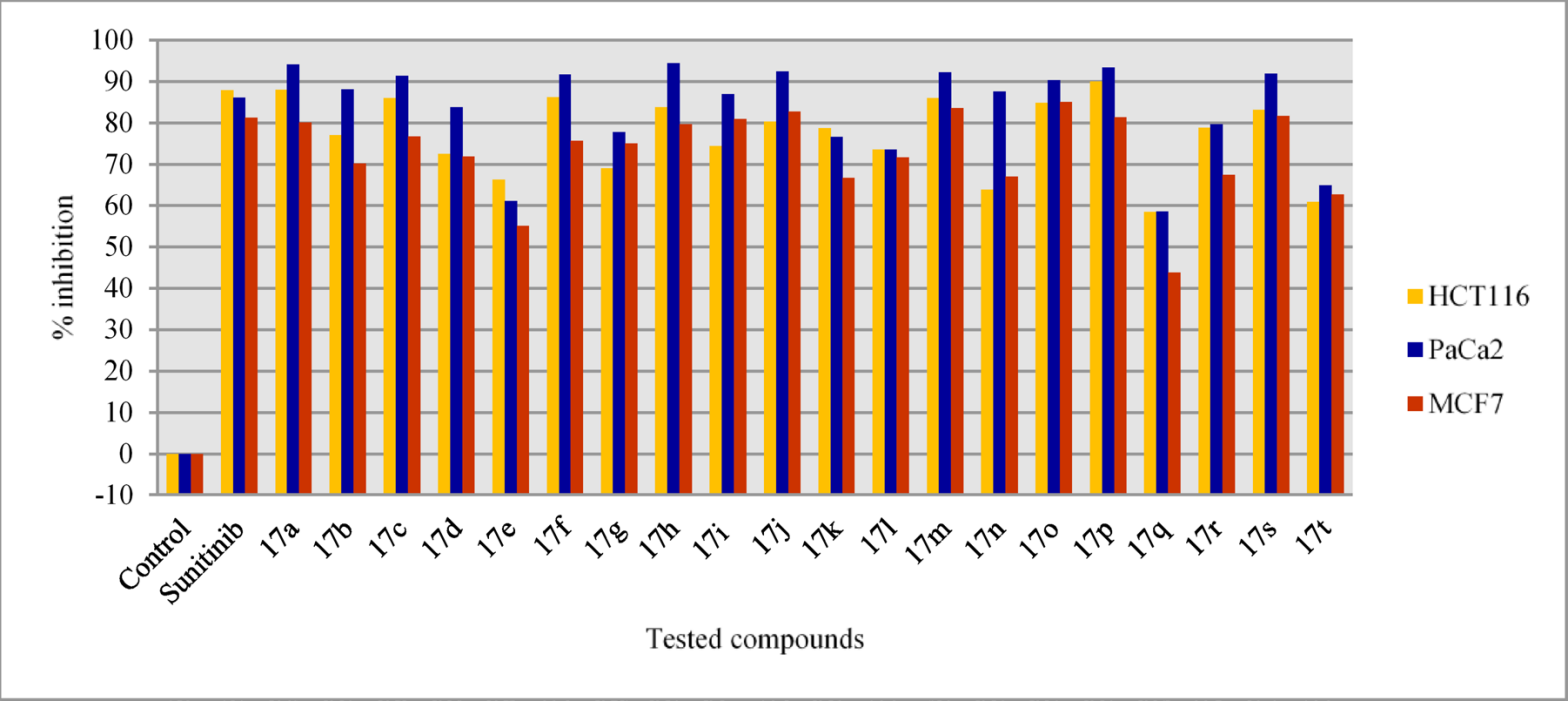
VEGFR-2 % inhibitory properties of the synthesized agents 17a‒t and sunitinib (standard drug) against HCT116, PaCa2, and MCF7 cancer cells.

**Fig. 9:**
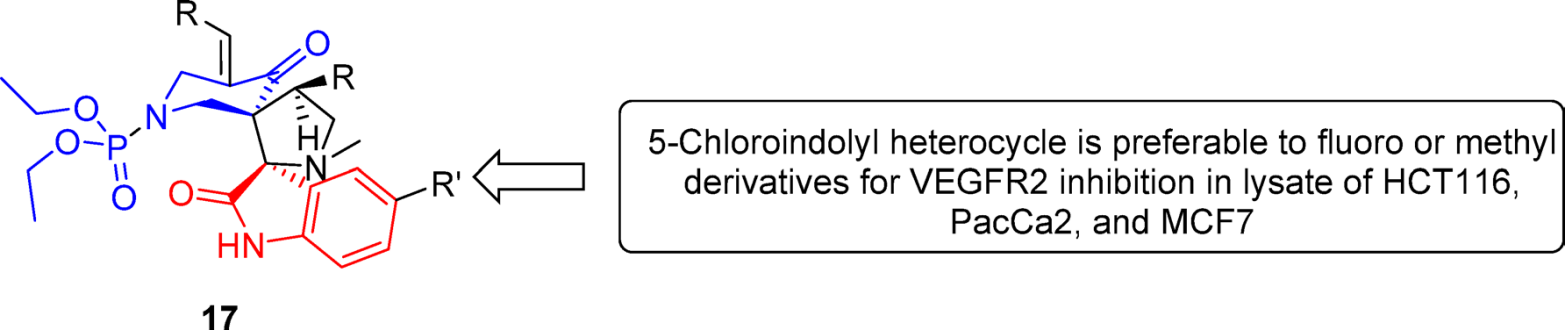
Summary of the most important items revealed through analysis of SARs due to VEGFR-2 inhibitory properties of 17a‒t utilizing the lysate of the tested cancer cell lines.

VEGFR-2 inhibitory properties of the synthesized agents 17a‒t were determined by the standard technique^63^ at 10 μM against HCT116, MCF7, and PaCa2 cancer cells and compared with the standard reference, sunitinib (Table 3, Figs. 8 and 9).

**Table 3:** The percentage VEGFR-2 inhibitory properties of the tested compounds at 10 μM.

Compd	HCT116		PaCa2		MCF7	
	*p*g/mL ± SEM	% Inhibition	*p*g/mL ± SEM	% Inhibition	*p*g/mL ± SEM	% Inhibition
17a	38.20 ± 1.6	88.0	19.26 ± 1.0	94.1	51.50 ± 0.8	80.1
17b	73.25 ± 2.0	77.0	38.75 ± 1.4	88.1	77.20 ± 0.6	70.2
17c	44.50 ± 1.1	86.0	28.43 ± 1.0	91.3	60.40 ± 0.5	76.7
17d	87.40 ± 1.9	72.5	53.42 ± 0.7	83.7	72.80 ± 1.2	71.9
17e	107.50 ± 2.0	66.2	127.43 ± 1.3	61.0	116.50 ± 1.0	55.0
17f	43.70 ± 0.9	86.2	27.53 ± 1.1	91.6	63.20 ± 0.9	75.6
17g	98.40 ± 1.2	69.0	72.80 ± 0.9	77.7	64.80 ± 0.5	75.0
17h	51.74 ± 1.5	83.7	18.25 ± 0.6	94.4	52.70 ± 0.7	79.6
17i	81.40 ± 1.9	74.4	42.76 ± 0.7	86.9	49.50 ± 0.3	80.9
17j	62.50 ± 0.7	80.3	24.78 ± 0.3	92.4	44.80 ± 0.6	82.7
17k	67.80 ± 1.3	78.7	76.40 ± 0.9	76.6	86.40 ± 0.5	66.6
17l	84.20 ± 1.5	73.5	86.45 ± 1.2	73.5	73.50 ± 0.8	71.6
17m	44.50 ± 0.5	86.0	25.36 ± 1.0	92.2	42.80 ± 0.4	83.5
17n	115.30 ± 2.2	63.7	40.70 ± 1.6	87.5	85.60 ± 0.7	66.9
17o	48.40 ± 0.6	84.8	31.64 ± 1.9	90.3	38.70 ± 0.4	85.0
17p	31.80 ± 0.8	90.0	21.76 ± 0.4	93.3	48.30 ± 0.4	81.3
17q	132.30 ± 0.9	58.4	135.70 ± 1.7	58.5	145.70 ± 1.9	43.7
17r	67.34 ± 0.4	78.8	66.53 ± 0.8	79.6	84.30 ± 1.0	67.4
17s	53.80 ± 0.9	83.1	26.50 ± 0.5	91.9	47.60 ± 0.6	81.6
17t	124.70 ± 1.3	60.8	115.00 ± 0.9	64.8	96.70 ± 1.2	62.6
Sunitinib	38.50 ± 1.0	87.9	45.30 ± 0.2	86.1	48.70 ± 1.0	81.2
Control	317.80 ± 3.5	–	326.80 ± 1.9	–	258.70 ± 2.3	–

##### HCT116

Synthesized agents 17p (R = 4-H_3_COC_6_H_4_, R′ = H) and 17a (R = Ph, R′ = H) revealed high VEGFR-2 inhibitory properties at 10 μM against HCT116 cancer cells with higher efficacies (% inhibition = 90.0 and 88.0, respectively) than that of the standard reference sunitinib (% inhibition = 87.9). Agents 17c (R = Ph, R′ = Cl), 17f (R = 4-FC_6_H_4_, R′ = Cl), and 17m (R = 4-H_3_CC_6_H_4_, R′ = H) also showed biochemical properties (% inhibition = 86.2‒ 86.0) close to that of sunitinib. Compounds 17h (R = 4-ClC_6_H_4_, R′ = H), 17o (R = 4-H_3_CC_6_H_4_, R′ = Cl), and 17s (R = 2-thienyl, R′ = Cl) also had considerable VEGFR-2 inhibitory effect (% inhibition = 84.8‒ 83.1).

SAR based on the observations indicated that the 5-chloroindolyl heterocycle is preferable to fluoro or methyl derivatives for VEGFR-2 inhibition as noted in analogs 17c/17b/17d (% inhibition = 86.0/77.0/72.5), 17f/17g (% inhibition = 86.2/69.0), 17j/17i/17k (% inhibition = 80.3/74.4/78.7), 17o/17n (% inhibition = 84.8/63.7), and 17s/17r/17t (% inhibition = 83.1/78.8/60.8).

##### PaCa2

Some of the synthesized agents [17a (R = Ph, R′ = H), 17h (R = 4-ClC_6_H_4_, R′ = H), and 17p (R = 4-H_3_COC_6_H_4_, R′ = H, % inhibition = 94.4‒93.3) are with distinguished anti-VEGFR-2 properties higher than that of sunitinib (% inhibition = 86.1) upon testing on PaCa2 cancer cells at 10 μM. Promising anti-VEGFR-2 properties (% inhibition = 92.4‒90.3) are also noted by compounds 17c (R = Ph, R′ = Cl), 17f (R = 4-FC_6_H_4_, R′ = Cl), 17j (R = 4-ClC_6_H_4_, R′ = Cl), 17m (R = 4-H_3_CC_6_H_4_, R′ = H), 17o (R = 4-H_3_CC_6_H_4_, R′ = Cl), and 17s (R = 2-thienyl, R′ = Cl).

SAR based on the observed anti-VEGFR-2 properties evidenced the importance of the chloro substituent linked at the 5-position of the indolyl heterocycle relative to the fluoro and methyl substitution for the anti-VEGFR-2 properties enhancement as noted in analogs 17c/17b/17d (% inhibition = 91.3/88.1/83.7), 17f/17g (% inhibition = 91.6/77.7), 17j/17i/17k (% inhibition = 92.4/86.9/76.6), 17o/17n (% inhibition = 90.3/87.5), and 17s/17r/17t (% inhibition = 91.9/79.6/64.8).

##### MCF7

Agents 17o (R = 4-H_3_CC_6_H_4_, R′ = Cl), 17m (R = 4-H_3_CC_6_H_4_, R′ = H), and 17j (R = 4-ClC_6_H_4_, R′ = Cl) have VEGFR-2 inhibitory effects (% inhibition = 85.0, 83.5, and 82.7, respectively) higher than that of the standard drug (sunitinib, % inhibition = 81.2) at 10 μM in breast cancer cell (MCF7). Compounds 17s (R = 2-thienyl, R′ = Cl), and 17p (R = 4-H_3_COC_6_H_4_, R′ = H) also have high efficacies (% inhibition = 81.6, 81.3). Other analogs, including 17i (R = 4-ClC_6_H_4_, R′ = F), and 17a (R = Ph, R′ = H), also have close inhibitory effect (% inhibition = 80.9, 80.1) to that of sunitinib.

SAR noted from the anti-VEGFR-2 properties show the favorable effect of chloro substituent of the indolyl heterocycle relative to the fluoro or methyl substituents as seen in analogs 17c/17b/17d (% inhibition = 76.7/70.2/71.9), 17f/17g (% inhibition = 75.6/75.0), 17j/17i/17k (% inhibition = 82.7/80.9/66.6), 17o/17n (% inhibition = 85.0/66.9), and 17s/17r/17t (% inhibition = 81.6/67.4/62.6). From these observations, it is clear that the attachment of a chloro substituent to the indolyl heterocycle at the 5-position enhances anti-VEGFR-2 properties relative to other elements/group (fluoro or methyl) in MCF7 in common with the other tested cell lines (HCT116 and PaCa2).

### COX-1/-2 inhibitory properties

Cyclooxygenases (COXs) are enzymes involved in the production of important bio-mediators including prostaglandins from arachidonic acid. Two main forms have been identified, COX-1 (expressed in many tissues with many biological functions including in gastric mucosa and kidney), and COX-2 (inducible during inflammation). Several non-steroidal anti-inflammatory drugs (NSAIDs) with COX inhibition have been discovered and clinically recommended for inflammation and pain symptoms. Few of them are known with selectivity towards COX-2^64^. Previous publications have mentioned the anti-inflammatory properties of both natural^65^, and synthetic spiro-indolin-2-ones^66^. Many reports have described the linkage between inflammation and some cancer types^67^. Some inflammatory mediators such as TNF-α (tumor necrosis factor-α)^68,69^, IL-6 (interleukin-6)^70,71^, and COX-2^72,73^ are also involved in cancer initiation, invasion, and development.

The synthesized agents 17a‒t were tested for COX-1/-2 activity by the standard techniques^74,75^ and compared with the known NSAIDs (ibuprofen and indomethacin) (Table 4). Compound 17g (R = 4-FC_6_H_4_, R′ = Me) is the most promising agent against COX-1 (% inhibition = 62.1) with slightly lower efficacy than the standard NASIDs (% inhibition = 65.3 and 64.3 for ibuprofen and indomethacin, respectively). Close efficacies were also noted for compounds 17l (R = 4-BrC_6_H_4_, R′ = H), 17o (R = 4-H_3_CC_6_H_4_, R′ = Cl), and 17q (R = 2-thienyl, R′ = H) (% inhibition = 61.7‒61.3).

**Table 4:** COX-1/-2 inhibitory properties of the tested agents at 10 μM.

Compd	% Inhibition of COX-1 ± SEM	% Inhibition of COX-2 ± SEM	SI
17a	49.8 ± 1.8	64.9 ± 2.8	0.96
17b	53.7 ± 3.7	61.3 ± 2.6	1.04
17c	58.6 ± 3.5	53.9 ± 1.7	1.05
17d	51.8 ± 2.2	56.4 ± 1.9	0.82
17e	55.0 ± 3.6	59.6 ± 1.3	1.08
17f	57.6 ± 1.1	60.8 ± 2.7	1.01
17g	62.1 ± 3.1	51.8 ± 2.8	0.96
17h	53.5 ± 2.8	51.5 ± 3.1	1.06
17i	46.2 ± 2.4	49.7 ± 1.9	1.09
17j	48.9 ± 1.5	53.5 ± 2.3	1.05
17k	57.7 ± 1.0	55.0 ± 1.6	1.30
17l	61.6 ± 1.2	50.3 ± 2.3	0.92
17m	58.3 ± 3.0	60.9 ± 1.1	0.93
17n	50.6 ± 2.3	47.3 ± 1.4	1.08
17o	61.7 ± 3.4	59.3 ± 2.4	1.14
17p	51.1 ± 2.7	53.5 ± 2.1	1.10
17q	61.3 ± 1.5	62.1 ± 3.1	0.93
17r	55.5 ± 2.6	51.8 ± 2.0	1.09
17s	53.8 ± 2.8	56.3 ± 2.3	0.83
17t	49.2 ± 1.9	63.7 ± 1.7	1.29
Ibuprofen	65.3 ± 1.2	71.3 ± 1.8	0.95
Indomethacin	64.3 ± 2.7	68.3 ± 1.6	1.09

Compound 17a (R = Ph, R′ = H) is the most promising agent against COX-2 (% inhibition = 64.9) being closest to the NSAIDs (% inhibition = 71.3 and 68.3 for ibuprofen and indomethacin, respectively). Notable properties are also observed for compounds 17t (R = 2-thienyl, R′ = Me), 17q (R = 2-thienyl, R′ = H), and 17b (R = Ph, R′ = F) (% inhibition = 63.7‒61.3). Slight enhancement of selectivity index (SI, COX-2/COX-1) was noted for 17k, and 17t (SI = 1.30 and 1.29, respectively), relative to the NSAIDs (SI = 0.95 and 1.09 for ibuprofen and indomethacin, respectively).

### TNF-α inhibitory properties

TNF-α is an important cytokine auto-produced by the immune system in association with different disorders including inflammation and cancer^76–79^. Concentration of TNF-α can be a therapeutic indicator for many diseases associated with inflammation and infectious diseases^80–82^. The synthesized compounds 17a‒t within the current study were assessed for TNF-α inhibitory properties by the standard technique and compared with those of NSAIDs (ibuprofen, and indomethacin)^83^. The results show that some of the synthesized agents have more promising inhibitory properties towards TNF-α relative to the standard drug used (Table 5). Compound 17h (R = 4-ClC_6_H_4_, R′ = H) is the most effective analog noted for HCT116 cell lysate (% inhibition at 10 μM = 80.7, 22.2 and 42.8, for 17h, ibuprofen and indomethacin; i.e. 3.6- and 1.9-fold the standards, respectively). Compounds 17m and 17a also show considerable biological properties (% inhibition = 77.4 and 74.0, respectively). Other synthesized agents with promising activity but with lower efficacies are 17i, 17k, 17l, 17p, and 17r (% inhibition = 72.6‒70.2).

**Table 5:** TNF-α inhibitory properties of the tested agents at 10 μM.

Compd	HCT116		PaCa2		MCF7	
	*p*g/mL (± SEM)	% inhibition	*p*g/mL (± SEM)	% inhibition	*p*g/mL (± SEM)	% inhibition
17a	24.86 ± 0.8	74.0	24.8 ± 1.5	77.8	18.25 ± 1.1	78.1
17b	94.64 ± 2.2	1.1	105.7 ± 3.9	5.5	82.25 ± 2.6	1.2
17c	31.72 ± 1.2	66.9	33.8 ± 2.2	69.8	23.16 ± 0.7	72.2
17d	94.80 ± 2.5	1.0	103.7 ± 4.2	7.2	83.2 ± 4.5	0.1
17e	94.27 ± 0.7	1.5	97.6 ± 1.6	12.7	81.87 ± 3.0	1.7
17f	85.46 ± 1.3	10.7	109.8 ± 4.7	7.2	82.45 ± 4.0	1.0
17g	81.75 ± 2.1	14.6	97.4 ± 3.6	12.9	83.0 ± 3.2	0.3
17h	18.45 ± 1.1	80.7	26.5 ± 0.7	76.3	21.65 ± 3.1	74.0
17i	28.56 ± 0.9	70.2	22.8 ± 1.6	79.6	31.27 ± 1.3	62.4
17j	81.78 ± 1.4	14.6	98.5 ± 3.1	11.9	82.87 ± 4.5	0.5
17k	26.48 ± 1.5	72.3	24.3 ± 1.4	78.3	22.85 ± 1.8	72.6
17l	27.34 ± 1.1	71.4	37.63 ± 0.8	66.3	33.67 ± 1.7	59.6
17m	21.65 ± 0.9	77.4	32.83 ± 1.3	70.6	26.24 ± 2.2	68.5
17n	86.47 ± 2.4	9.7	96.3 ± 3.0	13.9	83.17 ± 4.1	0.1
17o	86.34 ± 0.9	9.8	95.6 ± 2.2	14.5	81.85 ± 3.1	1.7
17p	26.21 ± 1.3	72.6	25.8 ± 1.8	76.9	28.56 ± 2.9	65.7
17q	84.26 ± 0.8	12.0	91.8 ± 2.7	17.9	80.67 ± 2.8	3.1
17r	27.45 ± 1.4	71.3	27.5 ± 0.5	75.4	25.46 ± 0.9	69.4
17s	86.57 ± 2.0	9.6	90.6 ± 2.8	19.0	81.64 ± 3.7	1.9
17t	81.64 ± 2.1	14.7	95.8 ± 2.5	14.3	82.91 ± 3.8	0.4
Ibuprofen	74.5 ± 1.1	22.2	64.8 ± 0.6	42.0	76.8 ± 2.0	7.8
Indomethacin	54.8 ± 0.7	42.8	75.3 ± 2.1	32.6	64.2 ± 1.6	22.9
Control	95.74 ± 0.5	–	111.8 ± 2.0	–	83.26 ± 1.1	–

Compound 17i (R = 4-ClC_6_H_4_, R′ = F) is the most effective agent upon testing with PaCa2 cell lysate (% inhibition = 79.6, 42.0 and 32.6 for 17i, ibuprofen and indomethacin; i.e. 1.9- and 2.4-fold the standards, respectively). Compounds 17a, 17 h, 17k, and 17p also reveal high TNF-α inhibitory properties (% inhibition = 78.3‒76.3).

The synthesized agent 17a (R = Ph, R′ = H) is the most promising agent observed upon assessment with MCF7 cell lysate (% inhibition = 78.1, 7.8 and 22.9 for 17a, ibuprofen and indomethacin, respectively). Compounds 17h, and 17b also exhibit promising activities (% inhibition = 74.0 and 72.2, respectively).

### Chick chorioallantoic membrane (CAM) studies

**Fig. 10:**
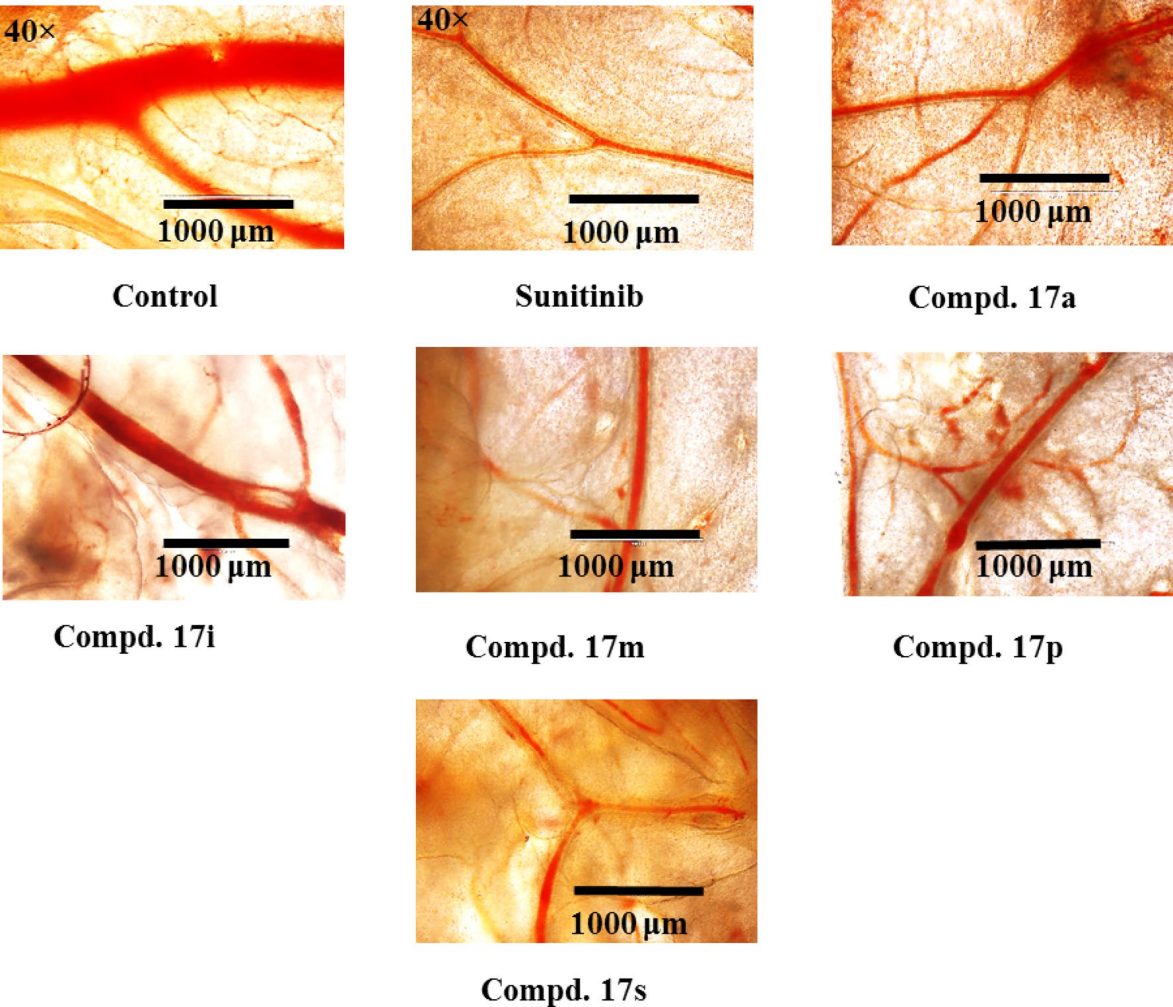
Images of light microscopy for anti-angiogenic evaluation of the tested compounds using CAM assay. A total amount of 20 μL (end concentration of 660 μM) of sunitinib and tested compounds were pipetted on glass slide and left to dry. The glass slide was placed on the surface of the CAM blood vessel network and left in an incubator at 37 °C for 3 days. The CAM blood vessel network was carefully cut and imaged under a light microscope (40 × magnification).

**Fig. 11:**
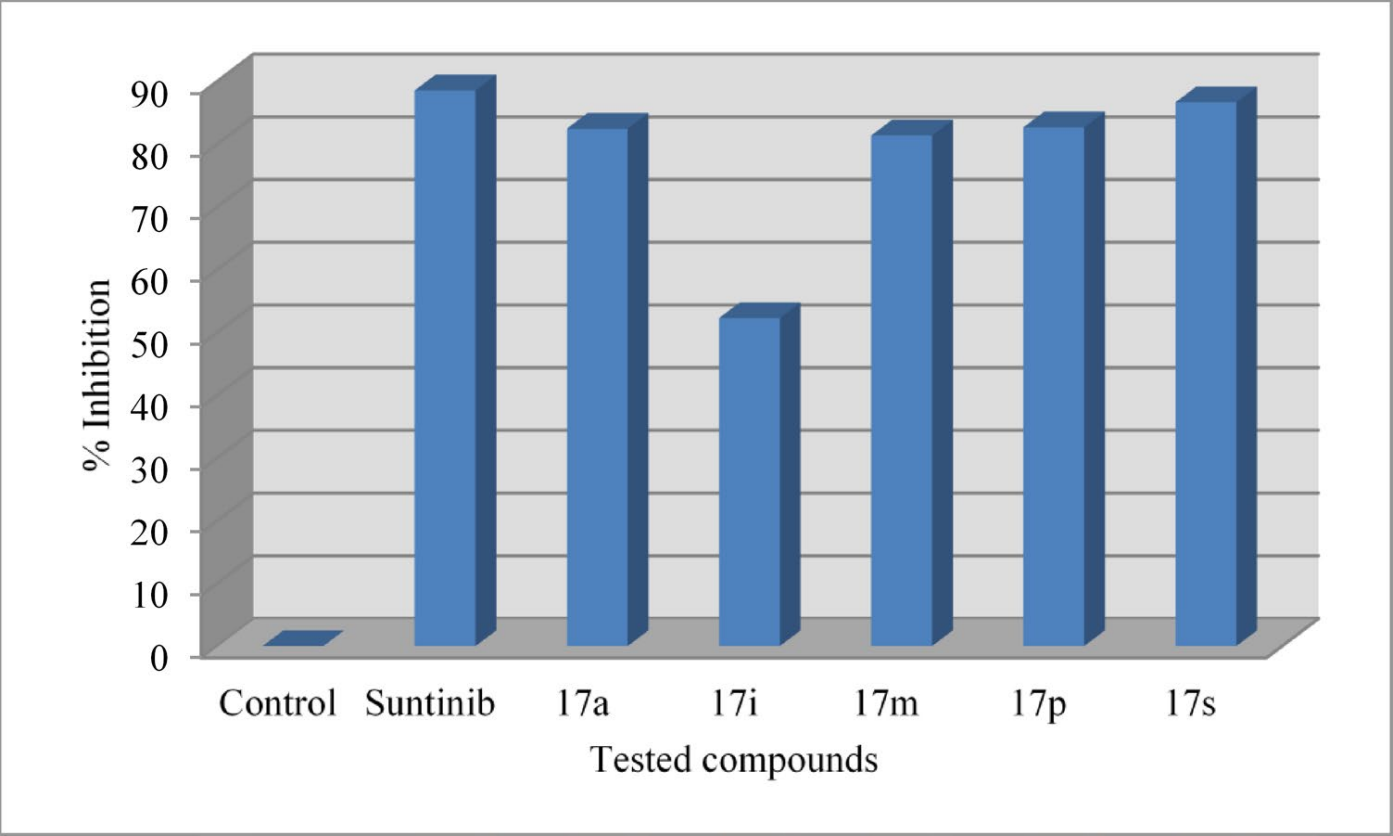
% inhibition of angiogenic effect of the tested compounds (17a, 17i, 17m, 17p, and 17s), and sunitinib (reference drug) using CAM assay.

CAM assay is an established xenograft technique for investigating the angiogenic effect due to the high resemblance of the human vascularized epithelium basement membrane with that of the chorioallantoic layer containing mainly type IV collagen^84–86^. Some of the promising anti-VEGFR-2 agents (17a, 17i, 17m, 17p, and 17s) were subjected for CAM testing studies and compared with that of sunitinib^23^ (a potent drug with VEGFR-2 inhibitory effect) (Table 6, Figs. 10 and 11). The results show that synthesized agent 17s (R = 2-thienyl, R′ = Cl) exhibits inhibitory effect in the CAM test (% inhibition = 86.7) close to that of the standard drug sunitinib (% inhibition = 88.6) which is consistent with its anti-VEGFR-2 properties relative to the standard drug tested (% anti-VEGFR-2 inhibition = 83.1/87.9, 81.6/81.2 and 91.9/86.1 for 17 s/sunitinib against HCT116, MCF7, and PaCa2 cancer cell lines, respectively). Promising anti-angiogenic properties (% inhibition = 82.7‒81.4) from the CAM test were also observed for compounds 17p (R = 4-H_3_COC_6_H_4_, R′ = H), 17a (R = Ph, R′ = H), and 17m (R = 4-H_3_CC_6_H_4_, R′ = H), consistent with the anti-VEGFR-2 properties (% anti-VEGFR-2 inhibition = 90.0/88.0/86.0, 81.3/80.1/83.5, 93.3/94.1/92.2 for HCT116/MCF7/ PaCa cancer cells, respectively). It should also be noted that the small differences between the antiproliferation, anti-VEGFR-2, and anti-angiogenic properties (Tables 1, 3, and 4) are attributable to the conditions applied for each procedure/protocol utilizing either human cell lines, kit enzyme, or xenograft model.

**Table 6:** % inhibitory properties of the blood vessels by the tested compound in CAM test.

Compd	Diameter of blood vessel (μm ± SEM)	% Inhibition
Control	459.7 ± 13.8	–
Sunitinib	52.3 ± 1.7	88.6
17a	80.7 ± 3.7	82.5
17i	219.3 ± 5.6	52.3
17m	85.7 ± 2.6	81.4
17p	79.3 ± 3.3	82.7
17s	61.3 ± 4.3	86.7

### QSAR studies

QSAR (quantitative SAR) is a computational chemical methodology useful not only for predicting biological properties quantitatively but also for determining the controlling functions and/or properties of biological active entities. Many medicinal chemical studies, including CODESSA-Pro software have described the successful utilization of this methodology ^87,88^.

#### HCT116

A robust four descriptor QSAR model (*R*^2^ = 0.954, *R*^2^cvOO = 0.901, *R*^2^cvMO = 0.911) was obtained for the anti-HCT116 training set compounds (17a‒e, g‒s, representing 90% of the synthesized agents) (Supplementary Tables S2‒S5, Fig. S30). Compounds 17f and 17t (IC_50_ = 3.716 and 7.342 μM, of potent and mild anti-HCT116 efficacies, respectively) were used as external test set analogs for validating the observed model. The QSAR model covers a wide range of anti-HCT116 properties [log IC_50_ (observed: 0.488551‒1.07041, predicted: 0.490357‒1.07339) μM] including potent and mild effective agents. Complementary information content (*t* “criterion value” = 7.792) is a topological descriptor with low coefficient value = 0.005. However, due to the high descriptor value of some respective analogs, a potential effect is noted for the predicted anti-HCT116 properties as shown in compounds 17d/17s (descriptor value = 220.206/150.022, estimated IC_50_ = 9.892/5.803 μM). Complementary information content can be calculated by equations (S1,S2)^89^.

The bond order for atom O is a semi-empirical descriptor (*t* = 5.685) with the highest coefficient value (13.0248) among all the model’s descriptors. This can justify the estimated anti-HCT116 values of compounds 17b/17h (descriptor value = 0.145/0.136, estimated anti-HCT116 IC_50_ = 8.233/3.302 μM).

Repulsion energy between two different atoms (C-N) is a semi-empirical descriptor with coefficient value = 0.353. Compound with high mathematical descriptor value gives rise to low anti-HCT116 predicted properties as noted in compounds 17d/17m (descriptor value = 168.125/167.772, estimated anti-HCT116 IC_50_ = 9.892/3.932 μM). The nuclear repulsion energy between two different atoms can be calculated by equation (S3)^89^.

Molecular volume is a geometrical descriptor with indication for the ability of a specific agent to be fitted in the protein/receptor active site so, revealing a potential bio-activity. It possesses a coefficient value with a negative sign (–0.010). So, analog(s) with high mathematical value can predicate a potent estimated anti-HCT116 effect as noted in compounds 17a/17o (descriptor value = 531.584/576.648, estimated anti-HCT116 IC_50_ = 7.039/3.728 μM). Molecular volume of an agent can be calculated by equation (S4)^89^.

##### PaCa2

A training set containing 17a,c‒g,i‒t (18 compounds out of 20 synthesized analogs of potential anti-PaCa2 properties) was used for optimizing a QSAR model. A validated 4-descriptor QSAR model was obtained (*R*^2^ = 0.959, *R*^2^cvOO = 0.927, *R*^2^cvMO = 0.930) covering a considerable range of anti-PaCa2 properties [1/IC_50_ (observed: 0.064‒0.1904, predicted: 0.067‒0.190) μM). Two of the synthesized analogs 17b and 17h were used as external test set compounds representing mild and promising anti-PaCa2 agents (Supplementary Tables S6‒S9, Fig. S31).

Rotational entropy is a thermodynamic descriptor (coefficient = 0.078). Analog(s) with high mathematical value can estimate high anti-PaCa2 properties due to 1/IC_50_ model assigned as noted in compounds 17 l/17q (descriptor value = 39.663/37.669, estimated IC_50_ = 5.952/13.873 μM). The rotational entropy can be calculated by equation (S5)^89^.

Resonance energy between two different atoms is a semi-empirical descriptor with coefficient value = 0.293, explaining the anti-PaCa2 properties of analogs 17a/17o (descriptor value = 17.469/17.782, estimated IC_50_ = 13.639/6.358 μM). Equation (S6) can calculate the resonance energy between two different atoms^89^.

Total interaction energy between two different atoms is also a semi-empirical descriptor with coefficient value = 0.115, evidencing the estimated anti-PaCa2 properties of compounds 17d/17t (descriptor value = 12.388/12.969, estimated IC_50_ = 15.024/9.106 μM). The descriptor values can be calculated by equation (S7)^89^.

Shadow plane XY is a geometrical descriptor with a negative sign coefficient value (‒0.003). Compounds with high mathematical value can estimate low anti-PaCa2 properties due to 1/IC_50_ modeling. This is noted in compounds 17i/17s (descriptor value = 139.24/102.38, estimated IC_50_ = 13.581/7.735 μM). Equation (S8) can calculate the shadow area of a specific molecule^89^.

##### Internal and external validations

The estimated antitumor properties relative to the noted experimental activities are the main internal validation elements evidencing the robust QSAR models obtained. Statistical values including leave-one-out and leave-many-out coefficients relative to the main coefficient value of the observed models (*R*^2^ = 0.954/0.959, *R*^2^cvOO = 0.901/0.927, *R*^2^cvMO = 0.911/0.930 for HCT116/PaCa2 models, respectively) are also important evidence for the goodness of the QSAR models. The standard deviation (*s*^2^) compare to Fisher criteria (*F*) are also important evidences for the robust QSAR models (*s*^2^ = 0.002/9.373e-005, *F* = 67.611/75.400 for HCT116/PaCa2 models, respectively).

External validation of the QSAR models was undertaken considering a potent and a mild antitumor agent (17f/17t, 17b/17h, for HCT116 and PaCa2 models, respectively). The estimated biological properties relative to the observed ones (estimate IC_50_: 3.063/7.067, observed IC_50_: 3.716/7.342 μM for 17f/17t as anti-HCT116, and estimated IC_50_: 11.096/8.191, observed IC_50_: 12.230/6.354 for 17b/17h as anti-PaCa2) are evidence for the goodness of these models which can be used for optimizing more potent analogs.

## Conclusion

Spiroindolin-2-ones with phosphonate function 17a‒t (20 analogs, 96‒72% yield) were constructed in regioselective mode following microwave synthetic methodology for azomethine cycloaddition with the appropriate 3,5-bis(ylidene)-4-piperidone-1-phosphonate 14a‒g. Different analytical techniques evidenced the structure, including single crystal X-ray studies of 17d. All the synthesized agents exhibited anti-HCT116 properties (MTT technique) with higher efficacies than the clinically approved drug, 5-fluorouracil. Compound 17h (R = 4-ClC_6_H_4_, R′ = H) is the most promising agent synthesized with potency 6.6- and 3.1-folds that of the standard drugs, 5-fluorouracil and sunitinib, respectively. Additionally, promising anti-PaCa2 properties were noted for the synthesized agents 17a‒t with higher potencies or close to that of sunitinib (clinically usable drug against pancreatic cancer). Compound 17f (R = 4-FC_6_H_4_, R′ = Cl) is the most promising anti-PaCa2 agent observed (3.2-fold that of sunitinib). It was observed that compound 17j (R = 4-ClC_6_H_4_, R′ = Cl) possesses anti-MCF7 potency close to that of 5-fluorouracil. Compound 17h (R = 4-ClC_6_H_4_, R′ = H; IC_50_ = 4.149 μM) is the most anti-A549 effective analog relative to doxorubicin. Some of the synthesized agents (17d, 17k, 17p, and 17s) revealed 3D-multilayer HCT116 cancer spheroid inhibitory properties. Needless to say that, the 3D-multicellular spheroid model is an important technique bridging the biological ability of a specific agent revealing a promising antiproliferation effect in the in vitro 2D-single layer culture with the in vivo animal model. Notable VEGFR-2 inhibitory properties were revealed by some of the synthesized analogs. The synthesized analogs also exhibited considerable COX-1/-2 inhibitory properties in addition to TNF-α relative to NSAIDs (ibuprofen and indomethacin, the clinically usable drugs). CAM testing supported the anti-VEGFR-2 observations and anti-angiogenic properties. Internal and external validated QSAR models explored the functions necessary for the antitumor properties.

## Experimental

### Chemical synthesis

Melting points were determined on a capillary point apparatus (Stuart SMP3) equipped with a digital thermometer. IR spectra (KBr) were recorded on a Shimadzu FT-IR 8400S spectrophotometer. Reactions were monitored using thin layer chromatography (TLC) on 0.2 mm silica gel F254 plates (Merck) utilizing various solvents for elution. The chemical structures of the synthesized compounds were characterized by nuclear magnetic resonance spectra (^1^H-NMR, ^13^C-NMR) and determined on a Bruker NMR spectrometer (500 MHz, 125 MHz for ^1^H and ^13^C, respectively). ^13^C-NMR spectra are fully decoupled. Chemical shifts were reported in parts per million (ppm) using the deuterated solvent peak or tetramethylsilane as an internal standard. The microwave oven used is a Milestone Italy (model: StartSynth, Reactor: Pack2B Basic Single Vessel Kit).

### Synthesis of dispiro[indoline-3,2′-pyrrolidine-3′,3′′-piperidin]-1′′-yl)phosphonates 17a‒t (general procedure)

A mixture of equimolar amounts of the appropriate diethyl [3,5-di((*E*)-ylidene)-4-oxopiperidin-1-yl]phosphonates 14a‒g (1.25 mmoL) and the corresponding isatins 15a‒d with sarcosine 16 in ethanol (10 mL) was heated in the microwave reactor at 60 °C (60 Watt) for 90 min. (hold time). After the completion of the reaction (TLC), the reaction mixture was allowed to cool at room temperature, and the solvent was evaporated under reduced pressure. The separated solid upon triturating the residual material with methanol (5 mL) was collected and crystallized from a suitable solvent affording the corresponding 17a‒t.

### Diethyl (*E*)-(5′′-benzylidene-1′-methyl-2,4′′-dioxo-4′-phenyldispiro[indoline-3,2′-pyrrolidine-3′,3′′-piperidin]-1′′-yl)phosphonate (17a)

Obtained from the reaction of 14a, 15a and 16, as pale yellow microcrystals from ethanol (92%), mp 181‒182°C^43^, and yield 92% (0.67 g).

### Diethyl (*E*)-(5′′-benzylidene-5-fluoro-1′-methyl-2,4′′-dioxo-4′-phenyldispiro[indoline-3,2′-pyrrolidine-3′,3′′-piperidin]-1′′-yl)phosphonate (17b)

Obtained from the reaction of 14a, 15b, and 16, as yellow microcrystals from methanol (80%), mp 212‒214 °C and, yield 82% (0.62 g). IR: *ν*_max_/cm^−1^ 3198, 1717, 1682, 1593, 1489, 1466, 1242, 1196, 1161, 1026. ^1^H-NMR (DMSO-*d*_*6*_) *δ* (ppm): 0.84 (t, *J* = 6.0 Hz, 3H, CH_3_), 0.99 (t, *J* = 6.0 Hz, 3H, CH_3_), 1.97 (s, 3H, NCH_3_), 2.16 (d, *J* = 13.4 Hz, 1H, upfield H of piperidinyl H_2_C-2′′), 3.31 (t, *J* = 8.5 Hz, 1H, upfield H of pyrrolidinyl H_2_C-5′), 3.44–3.54 (m, 2H, upfield H of upfield H of piperidinyl H_2_C-6′′ + downfield H of pyrrolidinyl H_2_C-5′), 3.67–3.89 (m, 6H, downfield H of piperidinyl H_2_C-2′′ + downfield H of piperidinyl H_2_C-6′′ + 2 OCH_2_), 4.65 (t, *J* = 9.2 Hz, 1H, pyrrolidinyl HC-4′), 6.63–6.68 (m, 2H, arom. H), 6.98 (t, *J* = 9.1 Hz, 1H, arom. H), 7.13 (d, *J* = 7.4 Hz, 2H, arom. H), 7.26 (t, *J* = 7.5 Hz, 1H, arom. H), 7.33–7.45 (m, 8H, 7 arom. H + olefinic CH), 10.55 (s, 1H, NH). ^13^C-NMR (DMSO-*d*_*6*_) *δ* (ppm): 15.51, 15.56, 15.75, 15.79 (CH_3_), 33.9 (NCH_3_), 45.4 (pyrrolidinyl HC-4′), 46.3 (piperidinyl H_2_C-6′′), 47.6 (piperidinyl H_2_C-2′′), 57.3 (pyrrolidinyl H_2_C-5′), 61.87, 61.91, 61.96, 62.27, 62.34 [spiro-C-3′ (C-3′′) + OCH_2_], 75.4 [spiro-C-3 (C-2′)], 109.87, 109.93, 114.1, 114.3, 115.2, 115.4, 127.0, 127.2, 127.3, 128.3, 128.6, 129.3, 129.5, 129.8, 131.7, 131.8, 134.0, 137.95, 137.98, 139.9, 156.6, 158.5 (arom. C + olefinic C), 175.2, 197.2 (C = O). Anal. Calcd. for C_33_H_35_FN_3_O_5_P (603.63): C, 65.66; H, 5.84; N, 6.96. Found: C, 65.52; H, 5.80; N, 7.03.

### Diethyl (*E*)-(5′′-benzylidene-5-chloro-1′-methyl-2,4′′-dioxo-4′-phenyldispiro[indoline-3,2′-pyrrolidine-3′,3′′-piperidin]-1′′-yl)phosphonate (17c)

Obtained from the reaction of 14a, 15c and 16, as buff microcrystals from ethanol (94%), mp 220‒221°C^43^, and yield 94% (0.72 g).

### Diethyl (*E*)-(5′′-benzylidene-1′,5-dimethyl-2,4′′-dioxo-4′-phenyldispiro[indoline-3,2′-pyrrolidine-3′,3′′-piperidin]-1′′-yl)phosphonate (17d)

Obtained from the reaction of 14a, 15d, and 16, as yellow microcrystals from methanol (80%), mp 210‒212 °C, and yield 91% (0.68 g). IR: *ν*_max_/cm^−1^ 3194, 1709, 1682, 1620, 1589, 1493, 1447, 1246, 1165, 1026. ^1^H-NMR (DMSO-*d*_*6*_) *δ* (ppm): 0.86 (t, *J* = 8.8 Hz, 3H, CH_3_), 0.98 (t, *J* = 8.9 Hz, 3H, CH_3_), 1.94 (s, 3H, NCH_3_), 2.12 (br s, 1H, upfield H of piperidinyl H_2_C-2′′), 2.17 (s, 3H, ArCH_3_), 3.27–3.34 (m, 2H, upfield H of pyrrolidinyl H_2_C-5′ + upfield H of upfield H of piperidinyl H_2_C-6′′), 3.49–3.54 (m, 1H, downfield H of pyrrolidinyl H_2_C-5′), 3.64–3.86 (m, 6H, downfield H of piperidinyl H_2_C-2′′ + downfield H of piperidinyl H_2_C-6′′ + 2 OCH_2_), 4.69 (t, *J* = 9.2 Hz, 1H, pyrrolidinyl HC-4′), 6.56–6.59 (m, 1H, arom. H), 6.70 (br s, 1H, arom. H), 6.93–6.94 (m, 1H, arom. H), 7.10 (br s, 2H, arom. H), 7.25–7.44 (m, 9H, 8 arom. H + olefinic CH), 10.39 (s, 1H, NH). ^13^C-NMR (DMSO-*d*_*6*_) *δ* (ppm): 15.58, 15.63, 15.76, 15.80 (CH_3_), 20.7 (ArCH_3_), 33.9 (NCH_3_), 45.3 (pyrrolidinyl HC-4′), 46.0 (piperidinyl H_2_C-6′′), 47.5 (piperidinyl H_2_C-2′′), 57.2 (pyrrolidinyl H_2_C-5′), 61.82, 61.86, 61.93 [spiro-C-3′ (C-3′′) + OCH_2_], 75.1 [spiro-C-3 (C-2′)], 125.4, 126.9, 127.5, 128.2, 128.5, 129.08, 129.16, 129.45, 129.54, 129.9, 131.8, 131.9, 134.2, 137.4, 138.2, 141.2 (arom. C + olefinic C), 175.1, 197.0 (C = O). Anal. Calcd. for C_34_H_38_N_3_O_5_P (599.67): C, 68.10; H, 6.39; N, 7.01. Found: C, 68.32; H, 6.22; N, 6.93.

### Diethyl (*E*)-[5′′-(4-fluorobenzylidene)-4′-(4-fluorophenyl)-1′-methyl-2,4′′-dioxodispiro[indoline-3,2′-pyrrolidine-3′,3′′-piperidin]-1′′-yl]phosphonate (17e)

Obtained from the reaction of 14b, 15a and 16, as pale yellow microcrystals from methanol, mp 193‒194°C^43^, and yield 95% (0.73 g).

### Diethyl (*E*)-[5-chloro-5′′-(4-fluorobenzylidene)-4′-(4-fluorophenyl)-1′-methyl-2,4′′-dioxodispiro[indoline-3,2′-pyrrolidine-3′,3′′-piperidin]-1′′-yl]phosphonate (17f)

Obtained from the reaction of **14b**, **15c** and **16**, as pale yellow microcrystals from n-butanol, mp 234‒235°C^43^, and yield 92% (0.76 g).

### Diethyl (*E*)-(5′′-(4-fluorobenzylidene)-4′-(4-fluorophenyl)-1′,5-dimethyl-2,4′′-dioxodispiro[indoline-3,2′-pyrrolidine-3′,3′′-piperidin]-1′′-yl)phosphonate (17g)

Obtained from the reaction of 14b, 15d, and 16, as pale yellow microcrystals from benzene, mp 216‒217 °C, and yield 78% (0.62 g). IR: *ν*_max_/cm^−1^ 3190, 1709, 1686, 1601, 1508, 1470, 1304, 1234, 1161, 1026. ^1^H-NMR (DMSO-*d*_*6*_) *δ* (ppm): 0.88 (t, *J* = 8.3 Hz, 3H, CH_3_), 1.00 (t, *J* = 7.2 Hz, 3H, CH_3_), 1.93 (s, 3H, NCH_3_), 2.16 (s, 3H, ArCH_3_), 2.19 (br s, 1H, upfield H of piperidinyl H_2_C-2′′), 3.30–3.32 (m, 2H, upfield H of pyrrolidinyl H_2_C-5′ + upfield H of upfield H of piperidinyl H_2_C-6′′), 3.49–3.57 (m, 1H, downfield H of pyrrolidinyl H_2_C-5′), 3.66–3.80 (m, 6H, downfield H of piperidinyl H_2_C-2′′ + downfield H of piperidinyl H_2_C-6′′ + 2 OCH_2_), 4.67 (t, *J* = 9.2 Hz, 1H, pyrrolidinyl HC-4′), 6.58 (d, *J* = 7.9 Hz, 1H, arom. H), 6.68 (s, 1H, arom. H), 6.93 (d, *J* = 7.9 Hz, 1H, arom. H), 7.14–7.18 (m, 4H, arom. H), 7.25 (t, *J* = 9.0 Hz, 2H, arom. H), 7.43–7.51 (m, 3H, arom. H + olefinic CH), 10.42 (s, 1H, NH). ^13^C-NMR (DMSO-*d*_*6*_) *δ* (ppm): 15.58, 15.63, 15.75, 15.80 (CH_3_), 20.6 (ArCH_3_), 33.8 (NCH_3_), 45.3 (pyrrolidinyl HC-4′), 45.4 (piperidinyl H_2_C-6′′), 47.4 (piperidinyl H_2_C-2′′), 57.6 (pyrrolidinyl H_2_C-5′), 61.6, 61.7, 61.84, 61.86, 61.89, 61.91 [spiro-C-3′ (C-3′′) + OCH_2_], 75.3 [spiro-C-3 (C-2′)], 108.9, 114.8, 115.0, 115.6, 115.8, 125.3, 127.4, 129.1, 129.5, 130.67, 130.70, 131.65, 131.73, 131.79, 131.9, 132.0, 134.38, 134.41, 136.2, 141.2, 160.2, 161.2, 162.1, 163.2 (arom. C + olefinic C), 175.2, 196.9 (C = O). Anal. Calcd. for C_34_H_36_F_2_N_3_O_5_P (635.65): C, 64.25; H, 5.71; N, 6.61. Found: C, 64.46; H, 5.85; N, 6.80.

### Diethyl (*E*)-[5′′-(4-chlorobenzylidene)-4′-(4-chlorophenyl)-1′-methyl-2,4′′-dioxodispiro[indoline-3,2′-pyrrolidine-3′,3′′-piperidin]-1′′-yl]phosphonate (17h)

Obtained from the reaction of 14c, 15a and 16, as pale yellow microcrystals from benzene ‒ light petroleum as 1:2 v/v, mp 143‒145°C^43^, and yield 91% (0.74 g).

### Diethyl (*E*)-(5′′-(4-chlorobenzylidene)-4′-(4-chlorophenyl)-5-fluoro-1′-methyl-2,4′′-dioxodispiro[indoline-3,2′-pyrrolidine-3′,3′′-piperidin]-1′′-yl)phosphonate (17i)

Obtained from the reaction of 14c, 15b, and 16, as yellow microcrystals from methanol, mp 208‒210 °C, and yield 93% (0.78 g). IR: *ν*_max_/cm^−1^ 3190, 1709, 1678, 1605, 1489, 1466, 1304, 1238, 1192, 1026. ^1^H-NMR (DMSO-*d*_*6*_) *δ* (ppm): 0.86 (t, *J* = 8.2 Hz, 3H, CH_3_), 1.02 (br s, 3H, CH_3_), 1.95 (s, 3H, NCH_3_), 2.23 (d, *J* = 13.5 Hz, 1H, upfield H of piperidinyl H_2_C-2′′), 3.33 (t, *J* = 8.7 Hz, 1H, upfield H of pyrrolidinyl H_2_C-5′), 3.43 (d, *J* = 15.5, Hz, 1H, upfield H of upfield H of piperidinyl H_2_C-6′′), 3.49–3.56 (m, 1H, downfield H of pyrrolidinyl H_2_C-5′), 3.69–3.83 (m, 6H, downfield H of piperidinyl H_2_C-2′′ + downfield H of piperidinyl H_2_C-6′′ + 2 OCH_2_), 4.61 (t, *J* = 9.4 Hz, 1H, pyrrolidinyl HC-4′), 6.61–6.68 (m, 2H, arom. H), 6.98 (t, *J* = 8.7 Hz, 1H, arom. H), 7.16 (d, *J* = 7.8 Hz, 2H, arom. H), 7.38–7.49 (m, 7H, 6 arom. H + olefinic CH), 10.59 (s, 1H, NH). ^13^C-NMR (DMSO-*d*_*6*_) *δ* (ppm): 15.55, 15.60, 15.76, 15.81 (CH_3_), 33.8 (NCH_3_), 45.5 (pyrrolidinyl HC-4′), 45.7 (piperidinyl H_2_C-6′′), 47.5 (piperidinyl H_2_C-2′′), 57.5 (pyrrolidinyl H_2_C-5′), 61.95, 61.99, 62.0, 62.1, 62.2 [spiro-C-3′ (C-3′′) + OCH_2_], 75.5 [spiro-C-3 (C-2′)], 110.0, 110.1, 114.1, 114.3, 115.3, 115.5, 126.98, 127.04, 128.2, 128.7, 128.8, 131.3, 131.7, 131.8, 132.1, 132.2, 132.3, 132.8, 134.1, 136.5, 137.0, 139.9, 156.6, 158.5 (arom. C + olefinic C), 175.2, 196.9 (C = O). Anal. Calcd. for C_33_H_33_Cl_2_FN_3_O_5_P (672.52): C, 58.94; H, 4.95; N, 6.25. Found: C, 59.06; H, 5.11; N, 6.34.

### Diethyl (*E*)-[5-chloro-5′′-(4-chlorobenzylidene)-4′-(4-chlorophenyl)-1′-methyl-2,4′′-dioxodispiro[indoline-3,2′-pyrrolidine-3′,3′′-piperidin]-1′′-yl]phosphonate (17j)

Obtained from the reaction of 14c, 15c and 16, as pale yellow microcrystals from methanol, mp 229‒230°C^43^, and yield 89% (0.76 g).

### Diethyl (*E*)-(5′′-(4-chlorobenzylidene)-4′-(4-chlorophenyl)-1′,5-dimethyl-2,4′′-dioxodispiro[indoline-3,2′-pyrrolidine-3′,3′′-piperidin]-1′′-yl)phosphonate (17k)

Obtained from the reaction of 14c, 15d, and 16, as yellow microcrystals from methanol, mp 208‒209 °C, and yield 95% (0.79 g). IR: *ν*_max_/cm^−1^ 3186, 1705, 1686, 1605, 1493, 1470, 1304, 1238, 1026. ^1^H-NMR (DMSO-*d*_*6*_) *δ* (ppm): 0.87 (t, *J* = 7.2 Hz, 3H, CH_3_), 1.00 (t, *J* = 8.6 Hz, 3H, CH_3_), 1.92 (s, 3H, NCH_3_), 2.16 (s, 3H, ArCH_3_), 2.21 (br s, 1H, upfield H of piperidinyl H_2_C-2′′), 3.30–3.33 (m, 2H, upfield H of pyrrolidinyl H_2_C-5′ + upfield H of upfield H of piperidinyl H_2_C-6′′), 3.49–3.54 (m, 1H, downfield H of pyrrolidinyl H_2_C-5′), 3.65–3.76 (m, 6H, downfield H of piperidinyl H_2_C-2′′ + downfield H of piperidinyl H_2_C-6′′ + 2 OCH_2_), 4.64 (t, *J* = 10.0 Hz, 1H, pyrrolidinyl HC-4′), 6.57 (d, *J* = 5.0 Hz, 1H, arom. H), 6.67 (s, 1H, arom. H), 6.93 (d, *J* = 7.8 Hz, 1H, arom. H), 7.14 (d, *J* = 8.3 Hz, 2H, arom. H), 7.39–7.49 (m, 7H, 6 arom. H + olefinic CH), 10.47 (s, 1H, NH). ^13^C-NMR (DMSO-*d*_*6*_) *δ* (ppm): 15.66, 15.71, 15.8, 15.9 (CH_3_), 20.7 (ArCH_3_), 33.9 (NCH_3_), 45.4 (pyrrolidinyl HC-4′), 45.5 (piperidinyl H_2_C-6′′), 47.5 (piperidinyl H_2_C-2′′), 57.5 (pyrrolidinyl H_2_C-5′), 61.7, 61.8, 61.94, 61.96, 62.00 [spiro-C-3′ (C-3′′) + OCH_2_], 75.3 [spiro-C-3 (C-2′)], 109.0, 125.2, 127.5, 128.2, 128.8, 129.2, 129.6, 131.4, 131.6, 131.9, 132.4, 132.5, 133.0, 134.0, 136.1, 137.3, 141.2 (arom. C + olefinic C), 175.2, 196.8 (C = O). Anal. Calcd. for C_34_H_36_Cl_2_N_3_O_5_P (668.55): C, 61.08; H, 5.43; N, 6.29. Found: C, 60.92; H, 5.35; N, 6.46.

### Diethyl (*E*)-[5′′-(4-bromobenzylidene)-4′-(4-bromophenyl)-1′-methyl-2,4′′-dioxodispiro[indoline-3,2′-pyrrolidine-3′,3′′-piperidin]-1′′-yl]phosphonate (17l)

Obtained from the reaction of 14d, 15a and 16, as pale yellow microcrystals from methanol, mp 137‒139°C^43^, and yield 85% (0.79 g).

### Diethyl (*E*)-[1′-methyl-5′′-(4-methylbenzylidene)-2,4′′-dioxo-4′-(*p*-tolyl)dispiro[indoline-3,2′-pyrrolidine-3′,3′′-piperidin]-1′′-yl]phosphonate (17m)

Obtained from the reaction of 14e, 15a and 16, as pale yellow microcrystals from methanol, mp 226‒227°C^43^, and yield 96% (0.73 g).

### Diethyl (*E*)-(5-fluoro-1′-methyl-5′′-(4-methylbenzylidene)-2,4′′-dioxo-4′-(p-tolyl)dispiro[indoline-3,2′-pyrrolidine-3′,3′′-piperidin]-1′′-yl)phosphonate (17n)

Obtained from the reaction of 14e, 15b, and 16, as yellow microcrystals from methanol, mp 179‒181 °C, and yield 93% (0.73 g). IR: *ν*_max_/cm^−1^ 3194, 1713, 1674, 1593, 1512, 1489, 1470, 1300, 1234, 1196, 1026. ^1^H-NMR (DMSO-*d*_*6*_) *δ* (ppm): 0.87 (br s, 3H, CH_3_), 1.01 (t, *J* = 8.3 Hz, 3H, CH_3_), 1.95 (s, 3H, NCH_3_), 2.16 (d, *J* = 13.3 Hz, 1H, upfield H of piperidinyl H_2_C-2′′), 2.28 (s, 3H, ArCH_3_), 2.30 (s, 3H, ArCH_3_), 3.44 (d, *J* = 15.2 Hz, 1H, upfield H of upfield H of piperidinyl H_2_C-6′′), 3.50–3.55 (m, 1H, upfield H of pyrrolidinyl H_2_C-5′), 3.66–3.86 (m, 7H, downfield H of pyrrolidinyl H_2_C-5′ + downfield H of piperidinyl H_2_C-2′′ + downfield H of piperidinyl H_2_C-6′′ + 2 OCH_2_), 4.60 (t, *J* = 9.3 Hz, 1H, pyrrolidinyl HC-4′), 6.61–6.66 (m, 2H, arom. H), 6.96 (t, *J* = 9.3 Hz, 1H, arom. H), 7.04 (d, *J* = 7.7 Hz, 2H, arom. H), 7.14 (d, *J* = 7.6 Hz, 2H, arom. H), 7.22 (d, *J* = 7.3 Hz, 2H, arom. H), 7.31 (d, *J* = 7.5 Hz, 2H, arom. H), 7.39 (s, 1H, olefinic CH), 10.53 (s, 1H, NH). ^13^C-NMR (DMSO-*d*_*6*_) *δ* (ppm): 15.6, 15.78, 15.83 (CH_3_), 20.6, 20.9 (ArCH_3_), 33.9 (NCH_3_), 45.5 (pyrrolidinyl HC-4′), 45.9 (piperidinyl H_2_C-6′′), 47.5 (piperidinyl H_2_C-2′′), 57.4 (pyrrolidinyl H_2_C-5′), 62.0, 62.2 [spiro-C-3′ (C-3′′) + OCH_2_], 75.4 [spiro-C-3 (C-2′)], 109.9, 128.9, 129.3, 129.7, 129.8, 130.5, 130.8, 131.2, 134.9, 136.1, 138.0, 139.4, 139.9 (arom. C + olefinic C), 175.2, 197.2 (C = O). Anal. Calcd. for C_35_H_39_FN_3_O_5_P (631.69): C, 66.55; H, 6.22; N, 6.65. Found: C, 66.77; H, 6.31; N, 6.76.

### Diethyl (*E*)-[5-chloro-1′-methyl-5′′-(4-methylbenzylidene)-2,4′′-dioxo-4′-(*p*-tolyl)dispiro[indoline-3,2′-pyrrolidine-3′,3′′-piperidin]-1′′-yl]phosphonate (17o)

Obtained from the reaction of 14e, 15c and 16, as pale yellow microcrystals from ethyl acetate, mp 227‒228°C^43^, and yield 93% (0.75 g).

### Diethyl (*E*)-[5′′-(4-methoxybenzylidene)-4′-(4-methoxyphenyl)-1′-methyl-2,4′′-dioxodispiro[indoline-3,2′-pyrrolidine-3′,3′′-piperidin]-1′′-yl]phosphonate (17p)

Obtained from the reaction of 14f., 15a and 16, as pale yellow microcrystals from methanol, mp 196‒198°C^43^, and yield 88% (0.75 g).

### Diethyl (*E*)-[1′-methyl-2,4′′-dioxo-4′-(thiophen-2-yl)-5′′-(thiophen-2-ylmethylene)dispiro[indoline-3,2′-pyrrolidine-3′,3′′-piperidin]-1′′-yl]phosphonate (17q)

Obtained from the reaction of 14g, 15a and 16, as pale yellow microcrystals from methanol, mp 206‒208°C^43^, and yield 84% (0.62 g).

### Diethyl (*E*)-(5-fluoro-1′-methyl-2,4′′-dioxo-4′-(thiophen-2-yl)-5′′-(thiophen-2-ylmethylene)dispiro[indoline-3,2′-pyrrolidine-3′,3′′-piperidin]-1′′-yl)phosphonate (17r)

Obtained from the reaction of 14e, 15b, and 16, as yellow microcrystals from methanol (80%), mp 216‒218 °C, and yield 77% (0.59 g). IR: *ν*_max_/cm^−1^ 3194, 1713, 1674, 1574, 1489, 1462, 1300, 1246, 1196, 1026. ^1^H-NMR (DMSO-*d*_*6*_) *δ* (ppm): 1.22 (t, *J* = 7.1 Hz, 3H, CH_3_), 1.25 (t, *J* = 7.3 Hz, 3H, CH_3_), 2.09 (s, 3H, NCH_3_), 2.58 (d, *J* = 13.5 Hz, 1H, upfield H of piperidinyl H_2_C-2′′), 3.53–3.59 (m, 2H, upfield H of pyrrolidinyl H_2_C-5′ + upfield H of upfield H of piperidinyl H_2_C-6′′), 3.78–4.13 (m, 7H, downfield H of pyrrolidinyl H_2_C-5′ + downfield H of piperidinyl H_2_C-2′′ + downfield H of piperidinyl H_2_C-6′′ + 2 OCH_2_), 5.00 (t, *J* = 9.2 Hz, 1H, pyrrolidinyl HC-4′), 6.70 (d, *J* = 8.6 Hz, 1H, arom. H), 6.81–6.83 (m, 1H, arom. H), 7.08 (t, *J* = 10.2 Hz, 1H, arom. H), 7.16 (br s, 1H, arom. H), 7.26 (br s, 1H, arom. H), 7.39 (br s, 1H, arom. H), 7.55 (br s, 1H, arom. H), 7.70–8.08 (m, 3H, 2 arom. H + olefinic CH), 10.70 (s, 1H, NH). ^13^C-NMR (DMSO-*d*_*6*_) *δ* (ppm): 15.85, 15.90, 15.94 (CH_3_), 33.7 (NCH_3_), 40.9 (pyrrolidinyl HC-4′), 45.6 (piperidinyl H_2_C-6′′), 46.0 (piperidinyl H_2_C-2′′), 58.4 (pyrrolidinyl H_2_C-5′), 61.4, 61.5, 61.96, 62.01, 62.06, 62.10 [spiro-C-3′ (C-3′′) + OCH_2_], 75.3 [spiro-C-3 (C-2′)], 109.89, 109.95, 113.7, 113.9, 115.2, 115.4, 125.0, 126.7, 126.9, 127.0, 127.2, 128.5, 130.4, 133.2, 135.2, 135.3, 137.0, 140.0, 140.7, 156.4, 158.3 (arom. C + olefinic C), 174.9, 195.6 (C = O). Anal. Calcd. for C_29_H_31_FN_3_O_5_PS_2_ (615.68): C, 56.58; H, 5.08; N, 6.83. Found: C, 56.44; H, 5.19; N, 6.67.

### Diethyl (*E*)-[5-chloro-1′-methyl-2,4′′-dioxo-4′-(thiophen-2-yl)-5′′-(thiophen-2-ylmethylene)dispiro[indoline-3,2′-pyrrolidine-3′,3′′-piperidin]-1′′-yl]phosphonate (17s)

Obtained from the reaction of 14g, 15c and 16, as pale yellow microcrystals from methanol, mp 223‒225°C^43^, and yield 80% (0.63 g).

### Diethyl (*E*)-(1′,5-dimethyl-2,4′′-dioxo-4′-(thiophen-2-yl)-5′′-(thiophen-2-ylmethylene)dispiro[indoline-3,2′-pyrrolidine-3′,3′′-piperidin]-1′′-yl)phosphonate (17t)

Obtained from the reaction of 14g, 15d, and 16, as yellow microcrystals from methanol, mp 202‒204 °C, and yield 72% (0.55 g). IR: *ν*_max_/cm^−1^ 3090, 1709, 1670, 1585, 1489, 1466, 1300, 1234, 1030. ^1^H-NMR (DMSO-*d*_*6*_) *δ* (ppm): 1.05–1.10 (m, 6H, 2 CH_3_), 1.92 (s, 3H, NCH_3_), 2.00 (s, 3H, ArCH_3_), 2.40 (d, *J* = 13.1 Hz, 1H, upfield H of piperidinyl H_2_C-2′′), 3.36 (d, *J* = 9.5 Hz, 2H, upfield H of pyrrolidinyl H_2_C-5′ + upfield H of upfield H of piperidinyl H_2_C-6′′), 3.65–3.93 (m, 7H, downfield H of pyrrolidinyl H_2_C-5′ + downfield H of piperidinyl H_2_C-2′′ + downfield H of piperidinyl H_2_C-6′′ + 2 OCH_2_), 4.90 (t, *J* = 8.1 Hz, 1H, pyrrolidinyl HC-4′), 6.54 (br s, 1H, arom. H), 6.61 (br s, 1H, arom. H), 6.86 (br s, 1H, arom. H), 7.01 (br s, 1H, arom. H), 7.11 (br s, 1H, arom. H), 7.23 (br s, 1H, arom. H), 7.40 (br s, 1H, arom. H), 7.51–7.89 (m, 3H, 2 arom. H + olefinic CH), 10.40 (s, 1H, NH). ^13^C-NMR (DMSO-*d*_*6*_) *δ* (ppm): 15.9 (CH_3_), 20.5 (ArCH_3_), 33.8 (NCH_3_), 40.5 (pyrrolidinyl HC-4′), 45.5 (piperidinyl H_2_C-6′′), 46.1 (piperidinyl H_2_C-2′′), 58.4 (pyrrolidinyl H_2_C-5′), 61.1, 62.0 [spiro-C-3′ (C-3′′) + OCH_2_], 75.1 [spiro-C-3 (C-2′)], 108.8, 124.92, 124.98, 127.0, 127.2, 127.4, 128.4, 129.0, 129.3, 129.9, 132.9, 134.7, 137.2, 141.0, 141.3 (arom. C + olefinic C), 174.9, 195.5 (C = O). Anal. Calcd. for C_30_H_34_N_3_O_5_PS_2_ (611.71): C, 58.91; H, 5.60; N, 6.87. Found: C, 58.74; H, 5.48; N, 6.73.

### Biological, biochemical and computational studies

The biological, biochemical and computational studies were mentioned in the supplementary file. The cell lines used in the current study were kindly gifted by Prof. Stig Linder, Karolinska Institute, Stockholm, Sweden, originally purchased from ATCC.

## Supplementary Information

Below is the link to the electronic supplementary material.

Supplementary Material 1

Supplementary Material 2

## Data Availability

All data generated or analyzed during this study are included in this published article and its supplementary material files. Crystallographic data for the structure reported in this paper **17d** has been deposited at the Cambridge Crystallographic Data Centre (CCDC) in the CSD under reference CCDC 2467603. These data can be obtained free of charge from the CCDC via [https://www.ccdc.cam.ac.uk/structures/](https://www.ccdc.cam.ac.uk/structures/).

## References

[1] Zhang, R., Yao, Y., Gao, H. & Hu, X. Mechanisms of angiogenesis in tumour. *Front. Oncol.* **14**, 1359069. 10.3389/fonc.2024.1359069 (2024). 38590656 10.3389/fonc.2024.1359069 PMC10999665

[2] Carmeliet, P. & Jain, R. K. Molecular mechanisms and clinical applications of angiogenesis. *Nature* **473**, 298–307. 10.1038/nature10144 (2011). 21593862 10.1038/nature10144 PMC4049445

[3] Liu, Z.-L., Chen, H.-H., Zheng, L.-L., Sun, L.-P. & Shi, L. Angiogenic signaling pathways and anti-angiogenic therapy for cancer. *Signal Transduct. Target. Ther.* **8**, 198. 10.1038/s41392-023-01460-1 (2023). 37169756 10.1038/s41392-023-01460-1 PMC10175505

[4] Nakamura, H. et al. Synthesis and biological evaluation of benzamides and benzamidines as selective inhibitors of VEGFR tyrosine kinases. *Bioorg. Med. Chem. Lett.* **16**, 5127–5131. 10.1016/j.bmcl.2006.07.075 (2006). 16893647 10.1016/j.bmcl.2006.07.075

[5] Sorafenib, uses, interactions, mechanism of action, (accessed 29 June 2025); https://go.drugbank.com/drugs/DB00398

[6] Nexavar (sorafenib) FDA approval history, (accessed 29 June 2025); https://www.drugs.com/history/nexavar.html

[7] Regorafenib, uses, interactions, mechanism of action, (accessed 29 June 2025); https://go.drugbank.com/drugs/DB08896

[8] Stivarga (Regorafenib) FDA Approval History, (accessed 29 June 2025); https://www.drugs.com/history/stivarga.html

[9] Lenvatinib mesylate, (accessed 29 June 2025); https://go.drugbank.com/salts/DBSALT001109

[10] Lenvima (lenvatinib) FDA approval history, (accessed 29 June 2025); https://www.drugs.com/history/lenvima.html

[11] Cabozantinib, uses, interactions, mechanism of action, (accessed 29 June 2025); https://go.drugbank.com/drugs/DB08875

[12] Cabometyx (cabozantinib) FDA Approval History, (accessed 29 June 2025); https://www.drugs.com/history/cabometyx.html

[13] Vandetanib, uses, interactions, mechanism of action, (accessed 29 June 2025); https://go.drugbank.com/drugs/DB05294

[14] Caprelsa (Vandetanib) FDA approval history, (accessed 29 June 2025); https://www.drugs.com/history/caprelsa.html

[15] Pazopanib, uses, interactions, mechanism of action, (accessed 29 June 2025); https://go.drugbank.com/drugs/DB06589

[16] Votrient (Pazopanib) FDA Approval History, (accessed 29 June 2025); https://www.drugs.com/history/votrient.html

[17] Axitinib, uses, interactions, mechanism of action, (accessed 29 June 2025); https://go.drugbank.com/drugs/DB06626

[18] Inlyta (Axitinib) FDA approval history, (accessed 29 June 2025); https://www.drugs.com/history/inlyta.html

[19] Sunitinib, uses, interactions, mechanism of action, (accessed 29 June 2025); https://go.drugbank.com/drugs/DB01268

[20] Sutent (sunitinib malate) FDA approval history, (accessed 29 June 2025); https://www.drugs.com/history/sutent.html

[21] Aboshouk, D. R. et al. Design and synthesis of antiproliferative 2-oxoindolin- 3-ylidenes incorporating urea function with potential VEGFR-2 inhibitory properties. *Sci. Rep.* **15**, 618. 10.1038/s41598-024-82005-6 (2025). 39753596 10.1038/s41598-024-82005-6 PMC11699130

[22] Thakur, A. et al. Recent advances and future directions on small molecule VEGFR inhibitors in oncological conditions. *Eur. J. Med. Chem.* **272**, 116472. 10.1016/j.ejmech.2024.116472 (2024). 38728867 10.1016/j.ejmech.2024.116472

[23] Girgis, A. S. et al. 3-Alkenyl-2-oxindoles: Synthesis, antiproliferative and antiviral properties against SARS-CoV-2. *Bioorg. Chem.* **114**, 105131. 10.1016/j.bioorg.2021.105131 (2021). 34243074 10.1016/j.bioorg.2021.105131 PMC8241580

[24] Abuzenadah, A. M. et al. Elucidating antiangiogenic potential of Rauwolfia serpentina: VEGFR-2 targeting-based molecular docking study. *Evid. Based Complement. Alternat. Med.*10.1155/2022/6224666 (2022). 35198035 10.1155/2022/6224666 PMC8860507

[25] Farghaly, T. A., Al-Hasani, W. A. & Abdulwahab, H. G. An updated patent review of VEGFR-2 inhibitors (2017-present). *Expert Opin. Ther. Pat.* **31**, 989–1007. 10.1080/13543776.2021.1935872 (2021). 34043477 10.1080/13543776.2021.1935872

[26] Seliem, I. A. et al. Development of isatin-based Schiff bases targeting VEGFR-2 inhibition: Synthesis, characterization, antiproliferative properties, and QSAR studies. *ChemMedChem* **17**, e202200164. 10.1002/cmdc.202200164 (2022). 35511203 10.1002/cmdc.202200164

[27] Aboshouk, D. R., Youssef, M. A., Bekheit, M. S., Hamed, A. R. & Girgis, A. S. Antineoplastic indole-containing compounds with potential VEGFR inhibitory properties. *RSC Adv.* **14**, 5690–5728. 10.1039/D3RA08962B (2024). 38362086 10.1039/d3ra08962b PMC10866129

[28] Hassan, S. M. et al. Indole compounds in oncology: therapeutic potential and mechanistic insights. *Pharmaceuticals* **17**, 922. 10.3390/ph17070922 (2024). 39065774 10.3390/ph17070922 PMC11280311

[29] Adefovir: Uses, interactions, mechanism of action, (accessed 29 June 2025); https://go.drugbank.com/drugs/DB13868

[30] Cidofovir: Uses, interactions, mechanism of action, (accessed 29 June 2025); https://go.drugbank.com/drugs/DB00369

[31] Zoledronic acid, (accessed 29 June 2025); https://go.drugbank.com/drugs/DB00399

[32] Adefovir dipivoxil: Uses, interactions, mechanism of action, (accessed June 29 2025); https://go.drugbank.com/drugs/DB00718

[33] Tenofovir disoproxil: Uses, interactions, mechanism of action, (accessed 29 June 2025); https://go.drugbank.com/drugs/DB00300

[34] Stella, V. J. & Nti-Addae, K. W. Prodrug strategies to overcome poor water solubility. *Adv. Drug Deliv. Rev.* **59**, 677–694. 10.1016/j.addr.2007.05.013 (2007). 17628203 10.1016/j.addr.2007.05.013

[35] Sauer, R. et al. Water-soluble phosphate prodrugs of 1-propargyl-8-styrylxanthine derivatives, A_2A_-selective adenosine receptor antagonists. *J. Med. Chem.* **43**, 440–448. 10.1021/jm9911480 (2000). 10669571 10.1021/jm9911480

[36] Lockbaum, G. J. et al. HIV-1 protease inhibitors with a P1 phosphonate modification maintain potency against drug-resistant variants by increased interactions with flap residues. *Eur. J. Med. Chem.* **257**, 115501. 10.1016/j.ejmech.2023.115501 (2023). 37244161 10.1016/j.ejmech.2023.115501 PMC10332405

[37] Girgis, A. S. et al. The therapeutic potential of spirooxindoles in cancer: A focus on p53–MDM2 modulation. *Pharmaceuticals* **18**, 274. 10.3390/ph18020274 (2025). 40006086 10.3390/ph18020274 PMC11859340

[38] Fawazy, N. G. et al. Development of spiro-3-indolin-2-one containing compounds of antiproliferative and anti-SARS-CoV-2 properties. *Sci. Rep.* **12**, 13880. 10.1038/s41598-022-17883-9 (2022). 35974029 10.1038/s41598-022-17883-9 PMC9380671

[39] Panda, S. S., Girgis, A. S., Aziz, M. N. & Bekheit, M. S. Spirooxindole: A versatile biologically active heterocyclic scaffold. *Molecules* **28**, 618. 10.3390/molecules28020618 (2023). 36677676 10.3390/molecules28020618 PMC9861573

[40] Girgis, A. S. et al. Synthesis, and QSAR analysis of anti-oncological active spiro-alkaloids. *Org. Biomol. Chem.* **13**, 1741–1753. 10.1039/C4OB02149E (2015). 25502495 10.1039/c4ob02149e

[41] Panda, S. S. et al. Rational design, synthesis and molecular modeling studies of novel anti-oncological alkaloids against melanoma. *Org. Biomol. Chem.* **13**, 6619–6633. 10.1039/C5OB00410A (2015). 25988330 10.1039/c5ob00410a

[42] Choudhury, C., Kumar, V. & Kumar, R. smProdrugs: A repository of small molecule prodrugs. *Eur. J. Med. Chem.* **249**, 115153. 10.1016/j.ejmech.2023.115153 (2023). 36724634 10.1016/j.ejmech.2023.115153

[43] Bekheit, M. S. et al. Spiroindole-containing compounds bearing phosphonate group of potential M^pro^-SARS-CoV-2 inhibitory properties. *Eur. J. Med. Chem.* **258**, 115563. 10.1016/j.ejmech.2023.115563 (2023). 37329713 10.1016/j.ejmech.2023.115563 PMC10257517

[44] Díaz-Carballo, D. et al. Therapeutic potential of antiviral drugs targeting chemorefractory colorectal adenocarcinoma cells overexpressing endogenous retroviral elements. *J. Exp. Clin. Cancer Res.* **34**, 81. 10.1186/s13046-015-0199-5 (2015). 26260344 10.1186/s13046-015-0199-5 PMC4542094

[45] Fawzy, N. G. et al. Novel curcumin inspired antineoplastic 1-sulfonyl-4-piperidones: Design, synthesis and molecular modeling studies. *Anti-Cancer Agents Med. Chem.* **19**, 1069–1078. 10.2174/1871520619666190408131639 (2019). 10.2174/1871520619666190408131639 30961509

[46] Fluorouracil injection Uses, Side Effects & Warnings, (accessed June 29 2025); https://www.drugs.com/mtm/fluorouracil-injection.html

[47] Fluorouracil topical Uses, Side Effects & Warnings, (accessed 29 June 2025); https://www.drugs.com/mtm/fluorouracil-topical.html

[48] Doxorubicin: Uses, Interactions, Mechanism of Action, (accessed 29 June 2025); https://go.drugbank.com/drugs/DB00997

[49] Roy, A., Raza, M. A. & Ghosh, V. Ajazuddin, diagnostic innovations and therapeutic potential of nanoparticulate delivery for colon cancer. *Nano-Struct. Nano-Obj.* **41**, 101426. 10.1016/j.nanoso.2024.101426 (2025).

[50] Gan, A. W., Kang, X., Goh, S. S., Wong, S. H. & Loo, S. C. J. Recent advances in the encapsulation of probiotics as functional foods for colorectal cancer prevention. *J. Funct. Foods* **128**, 106804. 10.1016/j.jff.2025.106804 (2025).

[51] Zhang, J. et al. Metabolic reprogramming of drug resistance in pancreatic cancer: Mechanisms and effects. *Mol Aspects Med.* **103**, 101368. 10.1016/j.mam.2025.101368 (2025). 40398192 10.1016/j.mam.2025.101368

[52] Sharma, R., Komal, K., Kuma, S., Ghosh, R. & Kumar, M. A comprehensive review on revolutionizing pancreatic cancer treatment: Liposomal innovations. *J. Drug Deli. Sci. Technol.* **110**, 107032. 10.1016/j.jddst.2025.107032 (2025).

[53] Zaki-Dizaji, M., Taheri, Z., Heiat, M. & Hushmandi, K. Tumor-educated platelet, a potential liquid biopsy biosource in pancreatic cancer: A review. *Pathol. Res. Pract.* **270**, 155986. 10.1016/j.prp.2025.155986 (2025). 40286788 10.1016/j.prp.2025.155986

[54] Gautam, S. et al. Understanding drug resistance in breast cancer: Mechanisms and emerging therapeutic strategies. *Medicine in Drug Discovery* **26**, 100210. 10.1016/j.medidd.2025.100210 (2025).

[55] Zhou, Z. et al. LAT1 transporter as a target for breast cancer diagnosis and therapy. *Eur. J. Med. Chem.* **283**, 117064. 10.1016/j.ejmech.2024.117064 (2025). 39631100 10.1016/j.ejmech.2024.117064

[56] Selvamuthukumar, K. et al. Recent advances in breath-based volatile compounds analysis by various analytical techniques for screening of lung cancer disease. *Microchem. J.* **214**, 114003. 10.1016/j.microc.2025.114003 (2025).

[57] Feng, Y. et al. Targeting CAFs and extracellular matrix (ECM) in lung cancer: Potential of adjuvants and nanoparticles. *Bioorg. Chem.* **162**, 108586. 10.1016/j.bioorg.2025.108586 (2025). 40398184 10.1016/j.bioorg.2025.108586

[58] Davoodi, F. et al. Theranostic applications of graphene-based materials in lung cancer: A review. *FlatChem* **51**, 100871. 10.1016/j.flatc.2025.100871 (2025).

[59] Lama, R. et al. Development, validation and pilot screening of an in vitro multi-cellular three-dimensional cancer spheroid assay for anti-cancer drug testing. *Bioorg. Med. Chem.* **21**, 922–931. 10.1016/j.bmc.2012.12.007 (2013). 23306053 10.1016/j.bmc.2012.12.007

[60] Mendes, V. I. S., Bartholomeusz, G. A., Ayres, M., Gandhi, V. & Salvador, J. A. R. Synthesis and cytotoxic activity of novel A-ring cleaved ursolic acid derivatives in human non-small cell lung cancer cells. *Eur. J. Med. Chem.* **123**, 317–331. 10.1016/j.ejmech.2016.07.045 (2016). 27484517 10.1016/j.ejmech.2016.07.045 PMC5652311

[61] Ivascu, A. & Kubbies, M. Rapid generation of single-tumor spheroids for high-throughput cell function and toxicity analysis. *J. Biomol. Screen.* **11**, 922–932. 10.1177/1087057106292763 (2006). 16973921 10.1177/1087057106292763

[62] Friedrich, J. et al. A reliable tool to determine cell viability in complex 3-D culture: The acid phosphatase assay. *J. Biomol. Screen.* **12**, 925–937. 10.1177/1087057107306839 (2007). 17942785 10.1177/1087057107306839

[63] Human VEGF R2 ELISA Kit, ELH-VEGFR2, RayBiotech, Peachtree Corners, GA, www.RayBiotech.com.

[64] Brown, L. D. et al. Novel isatin conjugates endowed with analgesic and anti-inflammatory properties: Design, synthesis and biological evaluation. *Future Med. Chem.* **17**, 59–73. 10.1080/17568919.2024.2437981 (2025). 39676545 10.1080/17568919.2024.2437981 PMC11703497

[65] Wang, G. et al. Two new neolignans and an indole alkaloid from the stems of *Nauclea officinalis* and their biological activities. *Fitoterapia* **160**, 105228. 10.1016/j.fitote.2022.105228 (2022). 35667521 10.1016/j.fitote.2022.105228

[66] Kumar, R. S. et al. Functionalized spirooxindole-indolizine hybrids: Stereoselective green synthesis and evaluation of anti-inflammatory effect involving TNF-α and nitrite inhibition. *Eur. J. Med. Chem.* **152**, 417–423. 10.1016/j.ejmech.2018.04.060 (2018). 29751235 10.1016/j.ejmech.2018.04.060

[67] Coussens, L. M. & Werb, Z. Inflammation and cancer. *Nature* **420**, 860–867. 10.1038/nature01322 (2002). 12490959 10.1038/nature01322 PMC2803035

[68] Zhao, H. et al. Inflammation and tumor progression: Signaling pathways and targeted intervention. *Signal Transduct. Target. Ther.* **6**, 263. 10.1038/s41392-021-00658-5 (2021). 34248142 10.1038/s41392-021-00658-5 PMC8273155

[69] Mercogliano, M. F., Bruni, S., Mauro, F., Elizalde, P. V. & Schillaci, R. Harnessing tumor necrosis factor alpha to achieve effective cancer immunotherapy. *Cancers* **13**, 564. 10.3390/cancers13030564 (2021). 33540543 10.3390/cancers13030564 PMC7985780

[70] Hirano, T. IL-6 in inflammation, autoimmunity and cancer. *Int. Immunol.* **33**, 127–148. 10.1093/intimm/dxaa078 (2021). 33337480 10.1093/intimm/dxaa078 PMC7799025

[71] Rašková, M. et al. The role of IL-6 in cancer cell invasiveness and metastasis-overview and therapeutic opportunities. *Cells* **11**, 3698. 10.3390/cells11223698 (2022). 36429126 10.3390/cells11223698 PMC9688109

[72] Bell, C. R. et al. Chemotherapy-induced COX-2 upregulation by cancer cells defines their inflammatory properties and limits the efficacy of chemoimmunotherapy combinations. *Nat. Commun.* **13**, 2063. 10.1038/s41467-022-29606-9 (2022). 35440553 10.1038/s41467-022-29606-9 PMC9018752

[73] Pu, D. et al. Cyclooxygenase-2 inhibitor: A potential combination strategy with immunotherapy in cancer. *Front. Oncol.* **11**, 637504. 10.3389/fonc.2021.637504 (2021). 33718229 10.3389/fonc.2021.637504 PMC7952860

[74] COX-1 inhibitor screening kit, fluorometric (catalog # K548–100), BioVision incorporated USA, www.biovision.com

[75] COX-2 inhibitor screening kit, fluorometric (catalog # K548–100), BioVision incorporated USA, www.biovision.com

[76] Shams, S. F. & Mehrad-Majd, H. TNFα-308G*>*a polymorphism and susceptibility to immune thrombocytopenia purpura (ITP): Evidence from a systematic review and meta-analysis. *Cytokine* **177**, 156538. 10.1016/j.cyto.2024.156538 (2024). 38368694 10.1016/j.cyto.2024.156538

[77] Jang, D. et al. The role of tumor necrosis factor alpha (TNF-α) in autoimmune disease and current TNF-α inhibitors in therapeutics. *Int. J. Mol. Sci.* **22**, 2719. 10.3390/ijms22052719 (2021). 33800290 10.3390/ijms22052719 PMC7962638

[78] Alizadeh, A. A., Morris, M. B., Church, W. B., Yaqoubi, S. & Dastmalchi, S. A mechanistic perspective, clinical applications, and phage-display-assisted discovery of TNFα inhibitors. *Drug Discovery Today* **27**, 503–518. 10.1016/j.drudis.2021.09.024 (2022). 34628042 10.1016/j.drudis.2021.09.024

[79] Zelova, H. & Hošek, J. TNF-a signalling and inflammation: interactions between old acquaintances. *Inflamm. Res.* **62**, 641–651. 10.1007/s00011-013-0633-0 (2013). 23685857 10.1007/s00011-013-0633-0

[80] Bradley, J. R. TNF-mediated inflammatory disease. *J. Pathol.* **214**, 149–160. 10.1002/path.2287 (2008). 18161752 10.1002/path.2287

[81] Zhang, H., Shi, N., Diao, Z., Chen, Y. & Zhang, Y. Therapeutic potential of TNFα inhibitors in chronic inflammatory disorders: Past and future. *Genes Diseases* **8**, 38–47. 10.1016/j.gendis.2020.02.004 (2021). 33569512 10.1016/j.gendis.2020.02.004 PMC7859422

[82] Valesini, G. et al. Biological and clinical effects of anti-TNFα treatment. *Autoimmun. Rev.* **7**, 35–41. 10.1016/j.autrev.2007.03.003 (2007). 17967723 10.1016/j.autrev.2007.03.003

[83] Enzyme-linked Immunosorbent Assay Kit, for Tumor Necrosis Factor Alpha (TNFa), Cloud-Clone Corp., 23603 W, Fernhurst Dr., Unit 2201, Katy, TX 77494, USA.

[84] Liu, M. et al. The histone methyltransferase EZH2 mediates tumor progression on the chick chorioallantoic membrane assay, a novel model of head and neck squamous cell carcinoma. *Transl. Oncol.* **6**, 273–281. 10.1593/tlo.13175 (2013). 23730406 10.1593/tlo.13175 PMC3660795

[85] Busch, C., Krochmann, J. & Drews, U. The chick embryo as an experimental system for melanoma cell invasion. *PLoS ONE* **8**, e53970. 10.1371/journal.pone.0053970 (2013). 23342051 10.1371/journal.pone.0053970 PMC3544663

[86] Schmiech, M. et al. Comparative investigation of frankincense nutraceuticals: Correlation of boswellic and lupeolic acid contents with cytokine release inhibition and toxicity against triple-negative breast cancer cells. *Nutrients* **11**, 2341. 10.3390/nu11102341 (2019). 31581678 10.3390/nu11102341 PMC6836131

[87] Girgis, A. S., Tala, S. R., Oliferenko, P. V., Oliferenko, A. A. & Katritzky, A. R. Computer-assisted rational design, synthesis, and bioassay of nonsteroidal anti-inflammatory agents. *Eur. J. Med. Chem.* **50**, 1–8. 10.1016/j.ejmech.2011.11.034 (2012). 22365409 10.1016/j.ejmech.2011.11.034

[88] Tiwari, A. D. et al. Microwave assisted synthesis and QSAR study of novel NSAID acetaminophen conjugates with amino acid linkers. *Org. Biomol. Chem.* **12**, 7238–7249. 10.1039/C4OB01281J (2014). 25081868 10.1039/c4ob01281j

[89] CODESSA-Pro manual, (accessed 29 June 2025); http://www.codessa-pro.com/manuals/manual.htm

