# Supplementary material for: Antiproliferation, 3D-multicellular spheroid and VEGFR-2 inhibitory properties of spiroindolin-2-ones with phosphonate function
Source: Sci Rep. 2025 Oct 7;15:35018. doi: 10.1038/s41598-025-20712-4 (PMC12504763; doi:10.1038/s41598-025-20712-4)

checkCIF/PLATON report

Structure factors have been supplied for datablock(s) bmk2359

THIS REPORT IS FOR GUIDANCE ONLY. IF USED AS PART OF A REVIEW PROCEDURE FOR PUBLICATION, IT SHOULD NOT REPLACE THE EXPERTISE OF AN EXPERIENCED CRYSTALLOGRAPHIC REFEREE.

No syntax errors found.   CIF dictionary   Interpreting this report

Datablock: bmk2359

```
Bond precision:   C-C = 0.0036 A          Wavelength=0.71073

Cell:            a=10.9380(6)      b=12.3350(7)      c=13.1714(5)
                 alpha=77.766(4)   beta=74.349(4)    gamma=71.855(5)
Temperature:     290 K
```

	Calculated	Reported
Volume	1610.14(15)	1610.14(15)
Space group	P -1	P -1
Hall group	-P 1	-P 1
Moiety formula	2(C34 H38 N3 O5 P), C H4 O	?
Sum formula	C69 H80 N6 O11 P2	C34.50 H40 N3 O5.50 P
Mr	1231.33	615.66
Dx,g cm-3	1.270	1.270
Z	1	2
Mu (mm-1)	0.133	0.133
F000	654.0	654.0
F000’	654.49	
h,k,lmax	15,17,18	14,16,18
Nref	9176	7626
Tmin,Tmax	0.938,0.988	0.445,1.000
Tmin’	0.926	

```
Correction method= # Reported T Limits: Tmin=0.445 Tmax=1.000
AbsCorr = GAUSSIAN

Data completeness= 0.831          Theta(max)= 29.739

R(reflections)= 0.0543( 5252)          wR2(reflections)= 0.1441( 7626)

S = 1.053                 Npar= 429
```

The following ALERTS were generated. Each ALERT has the format
test-name_ALERT_alert-type_alert-level.
Click on the hyperlinks for more details of the test.

Alert level B

```
PLAT772_ALERT_2_B Suspect O-H Bond in CIF: O6       --H35A      ..       1.45 Ang.
PLAT772_ALERT_2_B Suspect O-H Bond in CIF: O6       --H35B      ..       1.45 Ang.
PLAT772_ALERT_2_B Suspect O-H Bond in CIF: O6       --H35C      ..       1.45 Ang.
PLAT910_ALERT_3_B Missing FCF Reflection(s) Below Theta(Min)[Deg]=       3.32 Note
                1  0  0,  -1  1  0,   0  1  0,   1  1  0,  -1 -1  1,   0 -1  1,
               -1  0  1,   0  0  1,   1  0  1,   0  1  1,   1  1  1,   0  0  2,
```

Alert level C

```
PLAT041_ALERT_1_C Calc. and Reported SumFormula    Strings  Differ     Please Check
              Calc: C69 H80 N6 O11 P2
              Rep.: C34.50 H40 N3 O5.50 P
PLAT220_ALERT_2_C NonSolvent   Resd 1  C   Ueq(max)/Ueq(min) Range        3.3 Ratio
PLAT222_ALERT_3_C NonSolvent Resd 1  H   Uiso(max)/Uiso(min) Range        4.1 Ratio
PLAT260_ALERT_2_C Large Average Ueq of Residue Including        O6      0.157 Check
PLAT934_ALERT_3_C Number of (Iobs-Icalc)/Sigma(W) > 10 Outliers ..        1 Check
                3  0  0,
```

Alert level G

```
PLAT002_ALERT_2_G Number of Distance or Angle Restraints on AtSite          8 Note
PLAT003_ALERT_2_G Number of Uiso or U(i,j)  Restrained non-H-Atoms          6 Report
PLAT007_ALERT_5_G Number of Unrefined Donor-H Atoms .............          2 Report
              H1    H6A
PLAT045_ALERT_1_G Calculated and Reported Z Differ by a Factor ...      0.500 Check
PLAT168_ALERT_4_G The CIF-Embedded .res File Contains EXYZ Records          1 Report
PLAT171_ALERT_4_G The CIF-Embedded .res File Contains EADP Records          1 Report
PLAT172_ALERT_4_G The CIF-Embedded .res File Contains DFIX Records          1 Report
PLAT175_ALERT_4_G The CIF-Embedded .res File Contains SAME Records          1 Report
PLAT178_ALERT_4_G The CIF-Embedded .res File Contains SIMU Records          1 Report
PLAT186_ALERT_4_G The CIF-Embedded .res File Contains ISOR Records          1 Report
PLAT188_ALERT_3_G A Non-default SIMU Restraint Value has been used     0.0100 Report
PLAT189_ALERT_3_G A Non-default SAME Restraint Value for First Par     0.0100 Report
PLAT299_ALERT_4_G Atom Site Occupancy Constrained at ............        0.5 Check
              O6      H6A     H35A    H35B    H35C
PLAT301_ALERT_3_G Main Residue  Disorder .............(Resd    1)         7% Note
PLAT302_ALERT_4_G Anion/Solvent/Minor-Residue Disorder (Resd    2)        50% Note
PLAT779_ALERT_4_G Suspect or Irrelevant (Bond) Angle(s) in CIF ...      38.56 Deg.
              C35  -O6   -H35A    1_555   1_555   2_567 ......     #  173 Check
PLAT779_ALERT_4_G Suspect or Irrelevant (Bond) Angle(s) in CIF ...      38.56 Deg.
              C35  -O6   -H35B    1_555   1_555   2_567 ......     #  175 Check
PLAT779_ALERT_4_G Suspect or Irrelevant (Bond) Angle(s) in CIF ...      38.56 Deg.
              C35  -O6   -H35C    1_555   1_555   2_567 ......     #  178 Check
PLAT793_ALERT_4_G Model has Chirality at C8          (Centro SpGr)          R Verify
PLAT793_ALERT_4_G Model has Chirality at C10         (Centro SpGr)          S Verify
PLAT793_ALERT_4_G Model has Chirality at C11         (Centro SpGr)          R Verify
PLAT860_ALERT_3_G Number of Least-Squares Restraints ............        106 Note
PLAT883_ALERT_1_G Absent Datum for _atom_sites_solution_primary ..     Please Do !
PLAT912_ALERT_4_G Missing # of FCF Reflections Above STh/L=  0.600       1485 Note
```

```
PLAT941_ALERT_3_G Average HKL Measurement Multiplicity ..........        2.1 Low
PLAT969_ALERT_5_G The 'Henn et al.' R-Factor-gap value ..........      3.926 Note
              Predicted wR2: Based on SigI**2  3.67 or SHELX Weight 13.68
PLAT978_ALERT_2_G Number C-C Bonds with Positive Residual Density.          5 Info
```

```
   0 ALERT level A = Most likely a serious problem - resolve or explain
   4 ALERT level B = A potentially serious problem, consider carefully
   5 ALERT level C = Check. Ensure it is not caused by an omission or oversight
  27 ALERT level G = General information/check it is not something unexpected

   3 ALERT type 1 CIF construction/syntax error, inconsistent or missing data
   8 ALERT type 2 Indicator that the structure model may be wrong or deficient
   8 ALERT type 3 Indicator that the structure quality may be low
  15 ALERT type 4 Improvement, methodology, query or suggestion
   2 ALERT type 5 Informative message, check
```

It is advisable to attempt to resolve as many as possible of the alerts in all categories. Often the minor alerts point to easily fixed oversights, errors and omissions in your CIF or refinement strategy, so attention to these fine details can be worthwhile. In order to resolve some of the more serious problems it may be necessary to carry out additional measurements or structure refinements. However, the purpose of your study may justify the reported deviations and the more serious of these should normally be commented upon in the discussion or experimental section of a paper or in the "special_details" fields of the CIF. checkCIF was carefully designed to identify outliers and unusual parameters, but every test has its limitations and alerts that are not important in a particular case may appear. Conversely, the absence of alerts does not guarantee there are no aspects of the results needing attention. It is up to the individual to critically assess their own results and, if necessary, seek expert advice.

Publication of your CIF in IUCr journals

A basic structural check has been run on your CIF. These basic checks will be run on all CIFs submitted for publication in IUCr journals (*Acta Crystallographica*, *Journal of Applied Crystallography*, *Journal of Synchrotron Radiation*); however, if you intend to submit to *Acta Crystallographica Section C* or *E* or *IUCrData*, you should make sure that full publication checks are run on the final version of your CIF prior to submission.

Publication of your CIF in other journals

Please refer to the *Notes for Authors* of the relevant journal for any special instructions relating to CIF submission.

PLATON version of 04/06/2025; check.def file version of 30/05/2025

Datablock bmk2359 - ellipsoid plot

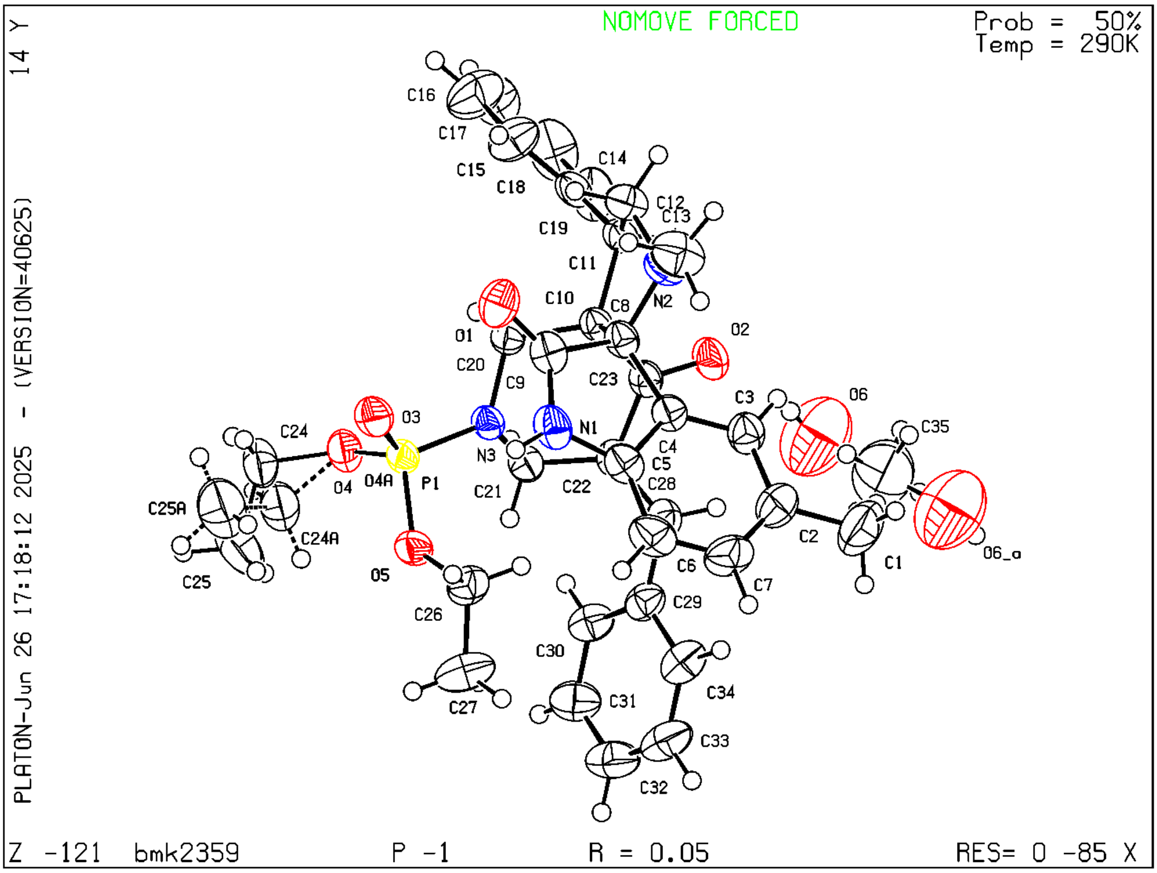

Supplement: Supplementary file 1 — Supplementary Material 1 [file 41598_2025_20712_MOESM1_ESM.pdf]
