# Supplementary material for: Antiproliferation, 3D-multicellular spheroid and VEGFR-2 inhibitory properties of spiroindolin-2-ones with phosphonate function
Source: Sci Rep. 2025 Oct 7;15:35018. doi: 10.1038/s41598-025-20712-4 (PMC12504763; doi:10.1038/s41598-025-20712-4)
Supplement: Supplementary file 2 — Supplementary Material 2 [file 41598_2025_20712_MOESM2_ESM.docx]

**Antiproliferation, 3D-multicellular spheroid and VEGFR-2 inhibitory properties of spiroindolin-2-ones with phosphonate function**

Sara M. Hassan^1^, Alyaa Farid^1^, Mohamed S. Bekheit^2,*^, Siva S. Panda^3,4^, Benson M. Kariuki^5^, Anwar Abdelnaser^6^, Soad Nasr^6^, Walid Fayad^7^, May A. El-Manawaty^7^, Ahmed A. F. Soliman^7^, Adel S. Girgis^2,^*

^1^Biotechnology Department, Faculty of Science, Cairo University, Giza, Egypt

^2^Department of Pesticide Chemistry, National Research Centre, Dokki, Giza 12622, Egypt

^3^Department of Chemistry and Biochemistry, Augusta University, Augusta, GA 30912, USA

^4^Department of Biochemistry and Molecular Biology, Augusta University, Augusta, GA 30912, USA

^5^School of Chemistry, Cardiff University, Main Building, Park Place, Cardiff, CF10 3AT, UK

^6^Institute of Global Health and Human Ecology, School of Sciences and Engineering, The American University in Cairo (AUC), Cairo 11835, Egypt

^7^Drug Bioassay-Cell Culture Laboratory, Pharmacognosy Department, National Research Centre, Dokki, Giza, 12622. Egypt

***** Correspondence authors: (Adel S. Girgis) [girgisas10@yahoo.com](mailto:girgisas10@yahoo.com) or [as.girgis@nrc.sci.eg](mailto:as.girgis@nrc.sci.eg); (Mohamed S. Bekheit) [M_bekheit@yahoo.com](mailto:M_bekheit@yahoo.com) or [mb.hassan@nrc.sci.eg](mailto:mb.hassan@nrc.sci.eg)

**Supplementary material**

**Table title**

**Table S1**. Crystal and structure refinement data for **17d**.

**Table S2.** Descriptors of the QSAR model for the training set compounds with properties against HCT116 (colon cancer) cell line.

**Table S3.** Observed and estimated antitumor properties (IC_50_, μM) for the training set compounds against HCT116 (colon) cancer cell line according to the BMLR-QSAR model.

**Table S4.** Molecular descriptor values of the QSAR model for the training set compounds with properties against HCT116 (colon) cancer cell line.

**Table S5.** Observed and estimated antitumor properties (IC_50_, μM) against HCT116 (colon) cancer cell line and molecular descriptor values of the QSAR model for the test set compounds.

**Table S6.** Descriptors of the QSAR model for the training set compounds with properties against PaCa2 (pancreatic cancer) cell line.

**Table S7.** Observed and estimated antitumor properties (IC_50_, μM) for the training set compounds against PaCa2 (pancreatic) cancer cell line according to the BMLR-QSAR model.

**Table S8.** Molecular descriptor values of the QSAR model for the training set compounds with properties against PaCa2 (pancreatic) cancer cell line.

**Table S9.** Observed and estimated antitumor properties (IC_50_, μM) against PaCa2 (pancreatic) cancer cell line and molecular descriptor values of the QSAR model for the test set compounds.

**Figure captions**

**Fig. S1.** IR spectrum of compound **17b** (KBr pellet).

**Fig. S2.** ^1^H-NMR spectrum of compound **17b** in DMSO-*d6*.

**Fig. S3.** ^13^C-NMR spectrum of compound **17b** in DMSO-*d6*.

**Fig. S4.** IR spectrum of compound **17d** (KBr pellet).

**Fig. S5.** ^1^H-NMR spectrum of compound **17d** in DMSO-*d6*.

**Fig. S6.** ^13^C-NMR spectrum of compound **17d** in DMSO-*d6*.

**Fig. S7.** IR spectrum of compound **17g** (KBr pellet).

**Fig. S8.** ^1^H-NMR spectrum of compound **17g** in DMSO-*d6*.

**Fig. S9.** ^13^C-NMR spectrum of compound **17g** in DMSO-*d6*.

**Fig. S10.** IR spectrum of compound **17i** (KBr pellet).

**Fig. S11.** ^1^H-NMR spectrum of compound **17i** in DMSO-*d6*.

**Fig. S12.** ^13^C-NMR spectrum of compound **17i** in DMSO-*d6*.

**Fig. S13.** IR spectrum of compound **17k** (KBr pellet).

**Fig. S14.** ^1^H-NMR spectrum of compound **17k** in DMSO-*d6*.

**Fig. S15.** ^13^C-NMR spectrum of compound **17k** in DMSO-*d6*.

**Fig. S16.** IR spectrum of compound **17n** (KBr pellet).

**Fig. S17.** ^1^H-NMR spectrum of compound **17n** in DMSO-*d6*.

**Fig. S18.** ^13^C-NMR spectrum of compound **17n** in DMSO-*d6*.

**Fig. S19.** IR spectrum of compound **17r** (KBr pellet).

**Fig. S20.** ^1^H-NMR spectrum of compound **17r** in DMSO-*d6*.

**Fig. S21.** ^13^C-NMR spectrum of compound **17r** in DMSO-*d6*.

**Fig. S22.** IR spectrum of compound **17t** (KBr pellet).

**Fig. S23.** ^1^H-NMR spectrum of compound **17t** in DMSO-*d6*.

**Fig. S24.** ^13^C-NMR spectrum of compound **17t** in DMSO-*d6*.

**Fig. S25.** Dose response curves of **17a‒t** against HCT116 (colon) cancer cell line.

**Fig. S26.** Dose response curves of **17a‒t** against PaCa2 (pancreatic) cancer cell line.

**Fig. S27.** Dose response curves of **17a‒t** against MCF7 (breast) cancer cell line.

**Fig. S28.** Dose response curves of **17a‒t** against A549 (lung) cancer cell line.

**Fig. S29.** Dose response curves of **17a‒t** against RPE1 normal/non-cancer cell line.

**Fig. S30.** QSAR plot representing the observed versus predicted training set compounds of anti-HCT116 properties [log(IC_50_) μM).

**Fig. S31.** QSAR plot representing the observed versus predicted training set compounds of anti-PaCa2 properties (1/IC_50_, μM).

**Crystal structure determination**

Single-crystal X-ray diffraction data for **17d** were collected on an Agilent SuperNova Dual Atlas diffractometer, equipped with a mirror monochromator and using either radiation. The data were processed using CrysAlisPro^1^ and the crystal structures were solved using SHELXT^2^ and refined using SHELXL^3^. In the structure, one CH_3_CH_2_O- of the diethylphosphonate group is disordered and was modeled with two components and they refined with occupancies of 0.824(6) and 0.176(6). The methanol solvent is disordered and was refined with the methyl carbon atom located on an inversion center. Non-hydrogen atoms were refined with anisotropic displacement parameters. In the final cycles of refinement, hydrogen atom geometry was idealized, and a riding model was used with U_iso_ set at 1.2 or 1.5 times the value of U_eq_ for the atom to which the hydrogen atoms are bonded. The crystal structure of **17d** has been deposited in the CSD under reference CCDC 2467603.

**Biological studies**

All the biological studies conducted obey the standards and approved by the Research Ethics Committee, National Research Centre, Egypt (associated with project ID: 13060103).

**2D-monolayer antiproliferation properties**

The cell lines used in the current study were kindly gifted by Prof. Stig Linder, Karolinska Institute, Stockholm, Sweden, originally purchased from ATCC. The synthesized compounds **17a‒t** were screened for their 2D-monolayer antiproliferation properties against HCT116 (colon), PaCa2 (pancreatic), MCF7 (breast), and A549 (lung) cancer cell lines by the standard mitochondrial dependent reduction of yellow MTT [3-(4,5-dimethylthiazol-2-yl)-2,5-diphenyl-tetrazolium bromide] to purple formazan technique^4,5^. Sunitinib, 5-fluorouracil, and doxorubicin were considered as standard references/drugs. Cells were suspended in DMEM medium for MCF7, PaCa-2 and McCoy’s 5A for HCT116, and A549 in addition to 1% antibiotic–antimycotic mixture (10000 μg mL^-1^ potassium penicillin, 10000 μg mL^-1^ streptomycin sulfate and 25 μg mL^-1^ amphotericin B), 10% fetal bovine serum and 1% L-glutamine at 37 °C, under 5% CO_2_ and 95% humidity. Cells were seeded at concentration of 30000 cells per well in fresh complete growth medium in 96-well tissue culture microtiter plates for 24 h. Media was aspirated, fresh complete medium was added and cells were incubated with different concentrations of the tested compound to give a final concentration of [50, 25, 12.5 and 6.25 μM “in addition to 3.125, 1.5625, 0.78125, and 0.390625 μM in case of high potent analogs”). 0.5% DMSO was used as a negative control. Triplicate wells were prepared for each individual dose. After 72 h of incubation, medium was aspirated, 40 μL MTT salt (2.5 mg mL^-1^) was added to each well and incubated for further 4 h at 37 °C. To stop the reaction and dissolve the formed crystals, 150 μL of 10% sodium dodecyl sulfate (SDS) in deionized water was added to each well and incubated overnight at 37 °C. The absorbance was then measured at 570 nm and a reference wavelength of 595 nm.

Data were collected as mean values for experiments performed in triplicates for each individual dose which had been measured by MTT assay. Control experiments did not exhibit significant change compared to the DMSO vehicle. The cell surviving fraction was calculated according to the following equation.

*Surviving fraction* = $\frac{Opticaldensity\left( O.D. \right)oftreatedcells}{O.D.ofcontrolcells}$

The agents synthesized were also tested against RPE1 (normal human immortalized retinal pigment epithelial cell line) cell (in DMEM-F12 medium) to determine the toxicity/selectivity towards normal cells relative to the cancer cell lines utilized.

The IC_50_ (concentration required to produce 50% inhibition of cell growth compared to the control experiment) was determined using Graph-Pad PRISM version-5 software. Statistical calculations for determination of the mean and standard error mean (SEM) values were determined by SPSS 16 software. The observed anti-proliferative properties are presented in Table 1 (Supplementary Figs. S S25‒S29).

**3D-multicellular spheroid**

Spheroids were prepared by the standard technique^6,7^. Briefly, cells were trypsinized, counted, and re-suspended in medium. A cell suspension containing 10,000 cells was added to each well of poly-HEMA-coated 96-well plates. Plates were centrifuged at 1000 RCF for 10 min, and then incubated at 37 °C in CO_2_ incubator for 5 days, media was changed daily. At the end of the experiment cytotoxicity was determined using the acid phosphatase method^7^. After washing of spheroids twice with 250 μL PBS buffer, 100 μL of 0.1 M sodium citrate, 0,1% Triton X-100, p-nitrophenyl phosphate (Pierce Biotechnology Inc, Rockford, IL) were added to each well and incubated for 1.5 h at 37 °C. After incubation, 10 μL 1N NaOH stop solution was added to each well and absorbance was read at 405 nm. Cytotoxicity was calculated according to the following equation:

[1-(av(x))/(av(c))]*100

Where, av: average, x: absorbance of sample, c: absorbance of control.

**VEGFR2 inhibitory properties**

The VEGFR-2 properties of the synthesized agents (**17a‒t**) and sunitinib (reference standard/drug) were determined at 10 μM by the standard technique obeying the manufacturer’s instruction (RayBiotech, Peachtree Corners)^8,9^.

**COX-1/2 inhibitory properties**

Inhibitory properties of COX-1 and -2 for the synthesized compound **17a‒t** and NSAIDs (ibuprofen, and indomethacin, standard reference/drug) were determined by the standard techniques obeying the manufacturer’s instructions at 10 μM^10,11^. The results were expressed as mean of 3 replicates ± SEM (Table 4).

**TNF-α inhibitory properties**

The TNF-α inhibitory properties of the synthesized compound **17a‒t** and NSAIDs (ibuprofen, and indomethacin) at 10 μM were determined by the enzyme-linked immunosorbent assay obeying the manufacturer’s instructions^12^. The results were expressed as mean of 3 replicates ± SEM (Table 5).

**Chick chorioallantoic membrane (CAM) studies**

Fertilized white chicken eggs were incubated in a 37 °C incubator with constant humidity. On day 8, a small squared window was opened allowing the detachment of the growing CAM and shell. The opening was covered with an elastic bandage. A stock concentration of 1 mM of **17a**, **17i**, **17m**, **17b**, and **17s** in DMSO was diluted with 1 mL-PBS (Phosphate-buffered saline ) and 0.5 mL absolute ethanol (2:1 respectively) to the desired final concentration, 660 *µ*M (sunitinib as positive control). On the following day, 20 µL of tested compounds (final concentration of 660 µM) were dried on a round 2.5 cm glass slide and placed on top of the vascularized CAM. The CAM was then sealed with a bandage and placed back in the incubator. CAMs were examined daily until day 12 (3 days), the vascularized membranes were then cut around the glass slide and fixed in 5% formalin. Images were taken with an Evos microscope at 40x and 100x magnification^9^. Main blood vessel in the microscopic images of controls and samples were quantified by imageJ and analyzed for significant differences (*n* = 4). The results were expressed in Table 6, Figs. 6,7.

**Computational studies (QSAR studies)**

The geometry of the tested agents was initially optimized by ChemBioOffice 2014 (ChemBio 3D Ultra 2014). 2D-QSAR studies were undertaken to utilize the comprehensive descriptors for structural and statistical analysis (CODESSA-Pro) software^13,14^. The optimized structures of the training set compounds (**17a‒e**,**g‒s**, and **17a**,**c‒g**,**i‒t** for anti-HCT116, and anti-PaCa2, respectively) were uploaded to CODESSA-Pro that includes MOPAC capability for the final geometry optimization. CODESSA-Pro calculated 852, 890 molecular descriptors for the anti-HCT116 and anti-PaCa2 compounds, respectively, including constitutional, topological, geometrical, charge-related, semi-empirical, molecular-type, atomic-type and bond-type descriptors (and thermodynamic in case of anti-PaCa2 agents) for the exported bio-active agents. Different mathematical transformations [including property (IC_50_), 1/property, log(property) and 1/log(property)] of the experimentally observed activity of the training set compounds were utilized searching for the best QSAR model. The best multi-linear regression (BMLR) technique was utilized which is a stepwise search for the best *n* parameter regression equations (where *n* stands for the number of descriptors used), based on the highest *R^2^* (squared correlation coefficient), *R^2^*cvOO (squared cross-validation “leave-one-out, LOO” coefficient), *R^2^*cvMO (squared cross-validation “leave-many-out, LMO” coefficient), *F* (Fisher statistical significance criteria) values, and *s* (standard deviation). The QSAR models were generated (obeying the thumb rule, which determines a reasonable ratio between the data points and the number of QSAR descriptor) (Supplementary Tables S1‒S8, Figs. S30,S31). The estimated properties of the test sat analogs (**17f**,**t** and **17b**,**h** for anti-HCT116, and anti-PaCa2, respectively) were calculated based on the observed BMLR-QSAR models.

The descriptors mentioned in the QSAR models can be calculated by the following equations^15^.

$${}^{k}{CIC}=\log_{2} n-{}^{k}{IC}\ldots\ldots(S1)$$

$${}^{k}{IC}=- \sum_{i-1}^{k} \frac{n_{i}}{n} \log_{2} \frac{n_{i}}{n}\ldots\ldots(S2)$$

Where, $n_{i}$ is the number of atoms in the *i*^th^ class, *n* is the total number of atoms in the molecule, and *k* is the number of atomic layers in the coordination sphere around a given atom that are accounted for.

$$E_{nn}\left( AB \right)= \frac{Z_{A}Z_{B}}{R_{AB}}\ldots\ldots(S3)$$

Where, *A* and *B* are two different atomic species; $Z_{A}$ and $Z_{B}$ are the charges of atomic nuclei *A* and *B*, respectively; and $R_{iB}$ is the distance between the atomic nuclei *A* and *B*.

$$V_{M}= \sum_{i} V_{VW}^{(i)}-V_{ov}\ldots\ldots(S4)$$

Where, $V_{VW}^{(i)}$ is the van der Waals volume of the i^th^ constituent atom of a molecule, and $V_{ov}$ is the volume of overlapping van der Waals atomic envelopes.

$$S_{rot}=Nk\ln\left[ \frac{\pi^{\frac{1}{2}}}{\sigma}\prod_{j-1}^{3} \left( \frac{8\pi^{2}I_{j}kT}{h^{2}} \right)^{\frac{1}{2}} \right]\ldots\ldots(S5)$$

Where, $I_{j}$ is the principal moments of inertia of the molecule, $\sigma$ is the symmetry number of the molecule, *h* is the Planck's constant, *k* is the Boltzmann's constant, and *T* is the absolute temperature (K).

$$E_{R}\left( AB \right)= \sum_{\mu\epsilon A} \sum_{\nu\epsilon B} P_{\mu\nu}\beta_{\mu\nu}\ldots\ldots(S6)$$

Where, *A* and *B* ate two different atomic species, $P_{\mu\nu}$ is the density matrix elements over atomic basis $\left\{ \mu\nu\right\}$, and $\beta_{\mu\nu}$ is the resonance integrals on atomic basis $\left\{ \mu\nu\right\}$.

$$E_{tot}\left( AB \right)= E_{C}\left( AB \right)+ E_{exc}\left( AB \right)\ldots\ldots(S7)$$

Where, *A* and *B* are two different atoms, $E_{C}(AB)$ is the electrostatic interaction energy between two atomic species, and $E_{exc}(AB)$ is the electronic exchange energy between two atomic species.

$$S_{k}= \frac{1}{2}\oint_{(C)} \left( \nu d\rho-\rho d\nu\right)\ldots\ldots(S8)$$

Where C is the contour of the projection of the molecule on the plane defined by two principal axes of the molecule (*k* = *XY, XZ* or *YZ*), $\nu-x or y$, and $\rho-y or z$ .

**References**

1. Rigaku, O. D. Yarnton, CrysAlisPro, England, 2022-24.
2. Sheldrick, G. M. SHELXT – Integrated space-group and crystal-structure determination. *Acta Crystallogr., Sect. A* **71**, 3‒8. <https://doi.org/10.1107/S2053273314026370> (2015).
3. Sheldrick, G. M. Crystal structure refinement with SHELXL. *Acta Crystallogr., Sect. C* **71**, 3‒8. <https://doi.org/10.1107/S2053229614024218> (2015).
4. Fawazy, N. G., Panda, S. S., Mostafa, A., Kariuki, B. M., Bekheit, M. S., Moatasim, Y., Kutkat, O., Fayad, W., El-Manawaty, M. A., Soliman, A. A. F., El-Shiekh, R. A., Srour, A. M., Barghash, R. F., Girgis, A. S. Development of spiro-3-indolin-2-one containing compounds of antiproliferative and anti-SARS-CoV-2 properties. *Sci. Rep.* **12**, 13880. <https://doi.org/10.1038/s41598-022-17883-9> (2022).
5. Fawzy, N. G., Panda, S. S., Fayad, W., El-Manawaty, M. A., Srour, A. M., Girgis, A. S. Novel curcumin inspired antineoplastic 1-sulfonyl-4-piperidones: design, synthesis and molecular modeling studies. *Anti-Cancer Agents in Med. Chem.* **19**, 1069‒1078. <https://doi:10.2174/1871520619666190408131639> (2019).
6. Ivascu, A., Kubbies, M. Rapid generation of single-tumor spheroids for high-throughput cell function and toxicity analysis. *J. Biomol, Screen.* **11**, 922‒932. DOI:10.1177/1087057106292763 (2006).
7. Friedrich, J. Eder, W., Castaneda, J., Doss, M., Huber, E., Ebner, R., Kunz-Schughart, L. A. A reliable tool to determine cell viability in complex 3-D culture: The acid phosphatase assay. *J. Biomol. Screen.* **12**, 925‒937. DOI:10.1177/1087057107306839 (2007).
8. Human VEGF R2 ELISA Kit, ELH-VEGFR2, RayBiotech, Peachtree Corners, GA, [www.RayBiotech.com](http://www.RayBiotech.com)
9. Girgis, A. S., Panda, S. S., Srour, A. M., Abdelnaser, A., Nasr, S., Moatasim, Y., Kutkat, O., El Taweel, A., Kandeil, A., Mostafa, A., Ali, M. A., Fawzy, N. G., Bekheit, M. S., Shalaby, E. M., Gigli, L., Fayad, W., Soliman, A. A. F. 3-Alkenyl-2-oxindoles: Synthesis, antiproliferative and antiviral properties against SARS-CoV-2. *Bioorg. Chem.* **114**, 105131. <https://doi.org/10.1016/j.bioorg.2021.105131> (2021).
10. COX-1 inhibitor screening kit, fluorometric (catalog # K548-100), BioVision incorporated USA, [www.biovision.com](http://www.biovision.com)
11. COX-2 inhibitor screening kit, fluorometric (catalog # K548-100), BioVision incorporated USA, [www.biovision.com](http://www.biovision.com)
12. Enzyme-linked Immunosorbent Assay Kit, for Tumor Necrosis Factor Alpha (TNFa), Cloud-Clone Corp., 23603 W, Fernhurst Dr., Unit 2201, Katy, TX 77494, USA.
13. Girgis, A. S., Tala, S. R., Oliferenko, P. V., Oliferenko, A. A., Katritzky, A. R. Computer-assisted rational design, synthesis, and bioassay of nonsteroidal anti-inflammatory agents. *Eur. J. Med. Chem.* **50**, 1–8, <https://doi.org/10.1016/j.ejmech.2011.11.034> (2012).
14. Tiwari, A. D., Panda, S. S., Girgis, A. S., Sahu, S., George, R. F., Srour, A. M., La Starza, B., Asiri, A. M., Hall,

 C. D. Katritzky, A. R. Microwave assisted synthesis and QSAR study of novel NSAID acetaminophen conjugates with amino acid linkers. *Org. Biomol. Chem.* **12**, 7238–7249. <https://doi.org/10.1039/C4OB01281J> (2014).
15. CODESSA-Pro manual, <http://www.codessa-pro.com/manuals/manual.htm> (access on Jun. 29, 2025).

**Table S1**. Crystal and structure refinement data for **17d**.

| Empirical formula | C_34_H_38_N_3_O_5_P, 0.5(CH_4_O) |
| --- | --- |
| Formula weight | 615.66 |
| Temperature | 290(2) K |
| Wavelength | 0.71073 Å |
| Crystal system | Triclinic |
| Space group | P -1 |
| a | 10.9380(6) Å |
| b | 12.3350(7) Å |
| c | 13.1714(5) Å |
| α | 77.766(4)° |
| β | 74.349(4)° |
| γ | 71.855(5)° |
| Volume | 1610.14(15) Å3 |
| Z | 2 |
| Density (calculated) | 1.270 Mg/m3 |
| Absorption coefficient | 0.133 mm-1 |
| F(000) | 654 |
| Crystal size | 0.580 x 0.400 x 0.090 mm3 |
| Theta range for data collection | 3.320 to 29.739° |
| Index ranges | -14<=h<=14, -16<=k<=16, -17<=l<=18 |
| Reflections collected | 15832 |
| Independent reflections | 7626 [R(int) = 0.0263] |
| Completeness to theta = 25.242° | 99.8 % |
| Refinement method | Full-matrix least-squares on F2 |
| Data / restraints / parameters | 7626 / 106 / 429 |
| Goodness-of-fit on F2 | 1.053 |
| Final R indices [I>2sigma(I)] | R1 = 0.0543, wR2 = 0.1223 |
| R indices (all data) | R1 = 0.0868, wR2 = 0.1441 |
| Largest diff. peak and hole | 0.215 and -0.358 e.Å-3 |

**Table S2.** Descriptors of the QSAR model for the training set compounds with properties against HCT116 (colon cancer) cell line.

| Entry | ID | Coefficient | *s* | *t* | Descriptor |
| --- | --- | --- | --- | --- | --- |
| 1 | 0 | –55.8306 | 12.092 | -4.617 | Intercept |
| 2 | *D*_1_ | 0.00482382 | 0.001 | 7.792 | Complementary information content (order 1) |
| 3 | *D*_2_ | 13.0248 | 2.291 | 5.685 | Min. (>0.1) bond order for atom O |
| 4 | *D*_3_ | 0.352914 | 0.072 | 4.921 | Max. n-n repulsion for bond C-N |
| 5 | *D*_4_ | –0.0100059 | 0.001 | -15.200 | Molecular volume |
| *N* = 18, *n* = 4, *R*^2^ = 0.954, *R*^2^cvOO = 0.901, *R*^2^cvMO = 0.911, *F* = 67.611, *s*^2^ = 0.002  IC_50_ = –55.8306 + (0.00482382 x *D*_1_) + (13.0248 x *D*_2_) + (0.352914 x *D*_3_) – (0.0100059 x *D*_4_) | | | | | |

**Table S3.** Observed and estimated antitumor properties (IC_50_, μM) for the training set compounds against HCT116 (colon) cancer cell line according to the BMLR-QSAR model.

| Entry | Compd. | Log(observed property) | Observed property | Log(estimated property) | Estimated property | Error*^a^* |
| --- | --- | --- | --- | --- | --- | --- |
| 1 | **17a** | 0.901513 | 7.971 | 0.847511 | 7.039 | 0.932 |
| 2 | **17b** | 0.86976 | 7.409 | 0.915547 | 8.233 | -0.824 |
| 3 | **17c** | 0.800511 | 6.317 | 0.827253 | 6.718 | -0.401 |
| 4 | **17d** | 0.967454 | 9.278 | 0.995294 | 9.892 | -0.614 |
| 5 | **17e** | 0.844415 | 6.989 | 0.792893 | 6.207 | 0.782 |
| 6 | **17g** | 0.699491 | 5.006 | 0.684739 | 4.839 | 0.167 |
| 7 | **17h** | 0.488551 | 3.08 | 0.518839 | 3.302 | -0.222 |
| 8 | **17i** | 0.61721 | 4.142 | 0.612876 | 4.101 | 0.041 |
| 9 | **17j** | 0.542452 | 3.487 | 0.568824 | 3.705 | -0.218 |
| 10 | **17k** | 0.516006 | 3.281 | 0.490357 | 3.093 | 0.188 |
| 11 | **17l** | 0.619302 | 4.162 | 0.617933 | 4.149 | 0.013 |
| 12 | **17m** | 0.536306 | 3.438 | 0.59465 | 3.932 | -0.494 |
| 13 | **17n** | 0.707144 | 5.095 | 0.721135 | 5.262 | -0.167 |
| 14 | **17o** | 0.574726 | 3.756 | 0.571531 | 3.728 | 0.028 |
| 15 | **17p** | 0.741309 | 5.512 | 0.671089 | 4.689 | 0.823 |
| 16 | **17q** | 1.07041 | 11.76 | 1.07339 | 11.841 | -0.081 |
| 17 | **17r** | 0.884795 | 7.67 | 0.847518 | 7.039 | 0.631 |
| 18 | **17s** | 0.733679 | 5.416 | 0.763646 | 5.803 | -0.387 |

*^a^* Error is the difference between the observed and estimated properties (IC_50_, μM).

**Table S4.** Molecular descriptor values of the QSAR model for the training set compounds with properties against HCT116 (colon) cancer cell line.

| Entry | Compd. | Descriptors*^a^* | | | |
| --- | --- | --- | --- | --- | --- |
|  |  | *D*_1_ | *D*_2_ | *D*_3_ | *D*_4_ |
| 1 | **17a** | 210.3449 | 0.13644 | 167.7612 | 531.584 |
| 2 | **17b** | 199.8473 | 0.14453 | 167.8567 | 533.624 |
| 3 | **17c** | 199.8473 | 0.14312 | 167.8932 | 541.908 |
| 4 | **17d** | 220.2055 | 0.1441 | 168.125 | 544.376 |
| 5 | **17e** | 193.5638 | 0.12988 | 168.2593 | 537.988 |
| 6 | **17g** | 203.8701 | 0.14341 | 167.8185 | 555.828 |
| 7 | **17h** | 193.5638 | 0.13562 | 167.8595 | 558.748 |
| 8 | **17i** | 183.5119 | 0.14384 | 168.1507 | 565.476 |
| 9 | **17j** | 189.0216 | 0.13751 | 168.2469 | 567.688 |
| 10 | **17k** | 203.8701 | 0.14076 | 167.9287 | 575.696 |
| 11 | **17l** | 193.5638 | 0.14575 | 168.003 | 567.096 |
| 12 | **17m** | 233.3367 | 0.13602 | 167.7715 | 567.76 |
| 13 | **17n** | 223.2847 | 0.13943 | 168.0322 | 563.912 |
| 14 | **17o** | 223.2847 | 0.1366 | 168.0739 | 576.648 |
| 15 | **17p** | 231.3367 | 0.1447 | 167.9397 | 576.384 |
| 16 | **17q** | 158.102 | 0.14214 | 168.1533 | 505.08 |
| 17 | **17r** | 150.0224 | 0.14149 | 167.917 | 514.572 |
| 18 | **17s** | 150.0224 | 0.13273 | 168.1878 | 521.1 |

*^a^* *D*_1_ = Complementary information content (order 1), *D*_2_ = Min. (>0.1) bond order for atom O, *D*_3_ = Max. n-n repulsion for bond C-N, *D*_4_ = Molecular volume.

**Table S5.** Observed and estimated antitumor properties (IC_50_, μM) against HCT116 (colon) cancer cell line and molecular descriptor values of the QSAR model for the test set compounds.

| Entry | Compd. | Descriptors*^a^* | | | | Observed property | Estimated property | Error*^b^* |
| --- | --- | --- | --- | --- | --- | --- | --- | --- |
|  |  | *D*_1_ | *D*_2_ | *D*_3_ | *D*_4_ |  |  |  |
| 1 | **17f** | 183.5119 | 0.13053 | 168.123 | 559.832 | 3.716 | 3.063 | 0.653 |
| 2 | **17t** | 169.6257 | 0.1434 | 167.834 | 523.416 | 7.342 | 7.067 | 0.275 |

*^a^* *D*_1_ = Complementary information content (order 1), *D*_2_ = Min. (>0.1) bond order for atom O, *D*_3_ = Max. n-n repulsion for bond C-N, *D*_4_ = Molecular volume.

*^b^* Error is the difference between the observed and estimated properties (IC_50_, μM).

**Table S6.** Descriptors of the QSAR model for the training set compounds with properties against PaCa2 (pancreatic cancer) cell line.

| Entry | ID | Coefficient | *s* | *t* | Descriptor |
| --- | --- | --- | --- | --- | --- |
| 1 | 0 | ‒9.14776 | 0.564 | -16.219 | Intercept |
| 2 | *D*_1_ | 0.0779238 | 0.006 | 13.137 | Rot. entropy (300K) |
| 3 | *D*_2_ | 0.293368 | 0.024 | 12.017 | Max. resonance energy for bond C-N |
| 4 | *D*_3_ | 0.115233 | 0.013 | 9.193 | Min. total interaction for bond C-C |
| 5 | *D*_4_ | ‒0.00279113 | 0.0002 | -9.397 | Shadow plane XY |
| *N* = 18, *n* = 4, *R*^2^ = 0.959, *R*^2^cvOO = 0.927, *R*^2^cvMO = 0.930, *F* = 75.400, *s*^2^ = 9.373e-005  IC_50_ = ‒9.14776 + (0.0779238 x *D*_1_) + (0.293368 x *D*_2_) + (0.115233 x *D*_3_) – (132.851 x *D*_4_) | | | | | |

**Table S7.** Observed and estimated antitumor properties (IC_50_, μM) for the training set compounds against PaCa2 (pancreatic) cancer cell line according to the BMLR-QSAR model.

| Entry | Compd. | 1/(observed property) | Observed property | 1/(estimated property) | Estimated property | Error*^a^* |
| --- | --- | --- | --- | --- | --- | --- |
| 1 | **17a** | 0.077399 | 12.92 | 0.073322 | 13.639 | -0.719 |
| 2 | **17c** | 0.109314 | 9.148 | 0.109214 | 9.156 | -0.008 |
| 3 | **17d** | 0.064226 | 15.57 | 0.066559 | 15.024 | 0.546 |
| 4 | **17e** | 0.105496 | 9.479 | 0.097047 | 10.304 | -0.825 |
| 5 | **17f** | 0.190404 | 5.252 | 0.189525 | 5.276 | -0.024 |
| 6 | **17g** | 0.16415 | 6.092 | 0.155736 | 6.421 | -0.329 |
| 7 | **17i** | 0.074349 | 13.45 | 0.073632 | 13.581 | -0.131 |
| 8 | **17j** | 0.1443 | 6.93 | 0.157772 | 6.338 | 0.592 |
| 9 | **17k** | 0.150173 | 6.659 | 0.137856 | 7.254 | -0.595 |
| 10 | **17l** | 0.162575 | 6.151 | 0.168008 | 5.952 | 0.199 |
| 11 | **17m** | 0.178412 | 5.605 | 0.171591 | 5.828 | -0.223 |
| 12 | **17n** | 0.065189 | 15.34 | 0.081996 | 12.196 | 3.144 |
| 13 | **17o** | 0.159413 | 6.273 | 0.157271 | 6.358 | -0.085 |
| 14 | **17p** | 0.136687 | 7.316 | 0.123437 | 8.101 | -0.785 |
| 15 | **17q** | 0.07485 | 13.36 | 0.072084 | 13.873 | -0.513 |
| 16 | **17r** | 0.120598 | 8.292 | 0.129278 | 7.735 | 0.557 |
| 17 | **17s** | 0.163026 | 6.134 | 0.171964 | 5.815 | 0.319 |
| 18 | **17t** | 0.105552 | 9.474 | 0.109823 | 9.106 | 0.368 |

*^a^* Error is the difference between the observed and estimated properties (IC_50_, μM).

**Table S8.** Molecular descriptor values of the QSAR model for the training set compounds with properties against PaCa2 (pancreatic) cancer cell line.

| Entry | Compd. | Descriptors*^a^* | | | |
| --- | --- | --- | --- | --- | --- |
|  |  | *D*_1_ | *D*_2_ | *D*_3_ | *D*_4_ |
| 1 | **17a** | 37.812 | 17.4688 | 12.9876 | 124.24 |
| 2 | **17c** | 38.072 | 17.6314 | 12.6967 | 123.72 |
| 3 | **17d** | 37.92 | 17.5966 | 12.3881 | 118.36 |
| 4 | **17e** | 38.219 | 17.5894 | 12.385 | 114.9 |
| 5 | **17f** | 38.574 | 17.7284 | 12.5219 | 111.94 |
| 6 | **17g** | 38.437 | 17.6951 | 12.8387 | 129.8 |
| 7 | **17i** | 38.84 | 17.5649 | 12.4138 | 139.24 |
| 8 | **17j** | 38.851 | 17.7333 | 12.5368 | 132.18 |
| 9 | **17k** | 38.853 | 17.5062 | 12.6913 | 121.88 |
| 10 | **17l** | 39.663 | 17.4602 | 12.906 | 137.72 |
| 11 | **17m** | 38.179 | 17.7046 | 12.9328 | 121.8 |
| 12 | **17n** | 38.33 | 17.5807 | 12.652 | 133.5 |
| 13 | **17o** | 38.506 | 17.7822 | 12.6897 | 134.18 |
| 14 | **17p** | 38.729 | 17.6682 | 12.6014 | 136.9 |
| 15 | **17q** | 37.669 | 17.6261 | 12.7196 | 126.16 |
| 16 | **17r** | 37.925 | 17.5547 | 12.6486 | 102.38 |
| 17 | **17s** | 38.261 | 17.748 | 12.8602 | 125.52 |
| 18 | **17t** | 37.974 | 17.4963 | 12.9693 | 117.82 |

*^a^* *D*_1_ = Rot. entropy (300K), *D*_2_ = Max. resonance energy for bond C-N, *D*_3_ = Min. total interaction for bond C-C, *D*_4_ = Shadow plane XY.

**Table S9.** Observed and estimated antitumor properties (IC_50_, μM) against PaCa2 (pancreatic) cancer cell line and molecular descriptor values of the QSAR model for the test set compounds.

| Entry | Compd. | Descriptors*^a^* | | | | Observed property | Estimated property | Error*^b^* |
| --- | --- | --- | --- | --- | --- | --- | --- | --- |
|  |  | *D*_1_ | *D*_2_ | *D*_3_ | *D*_4_ |  |  |  |
| 1 | **17b** | 37.96 | 17.6523 | 12.5439 | 123.32 | 12.23 | 11.096 | 1.134 |
| 2 | **17h** | 38.759 | 17.5751 | 12.6499 | 130.44 | 6.354 | 8.191 | -1.837 |

*^a^* *D*_1_ = Rot. entropy (300K), *D*_2_ = Max. resonance energy for bond C-N, *D*_3_ = Min. total interaction for bond C-C, *D*_4_ = Shadow plane XY.

*^b^* Error is the difference between the observed and estimated properties (IC_50_, μM).


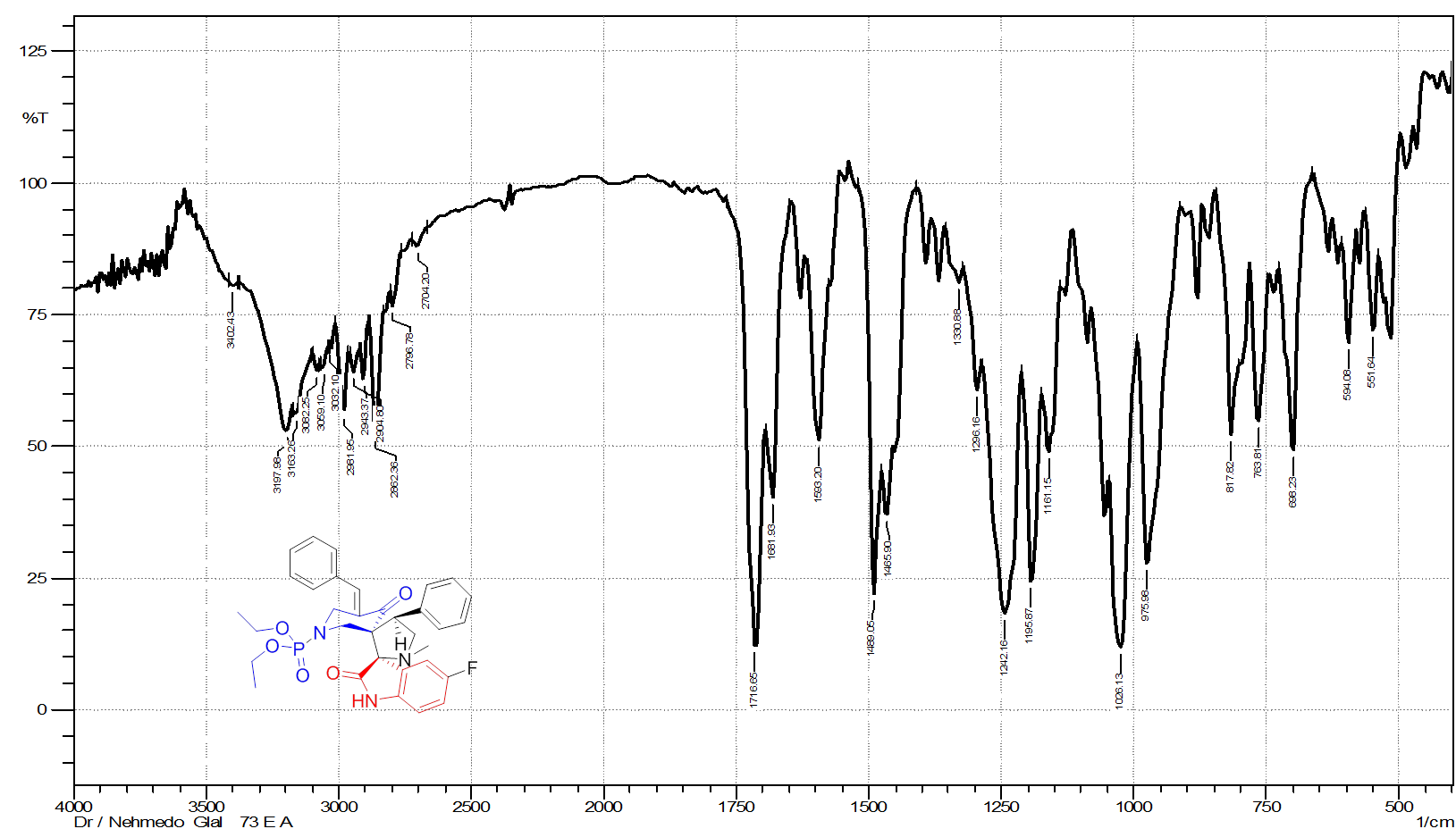


**Fig. S1.** IR spectrum of compound **17b** (KBr pellet).


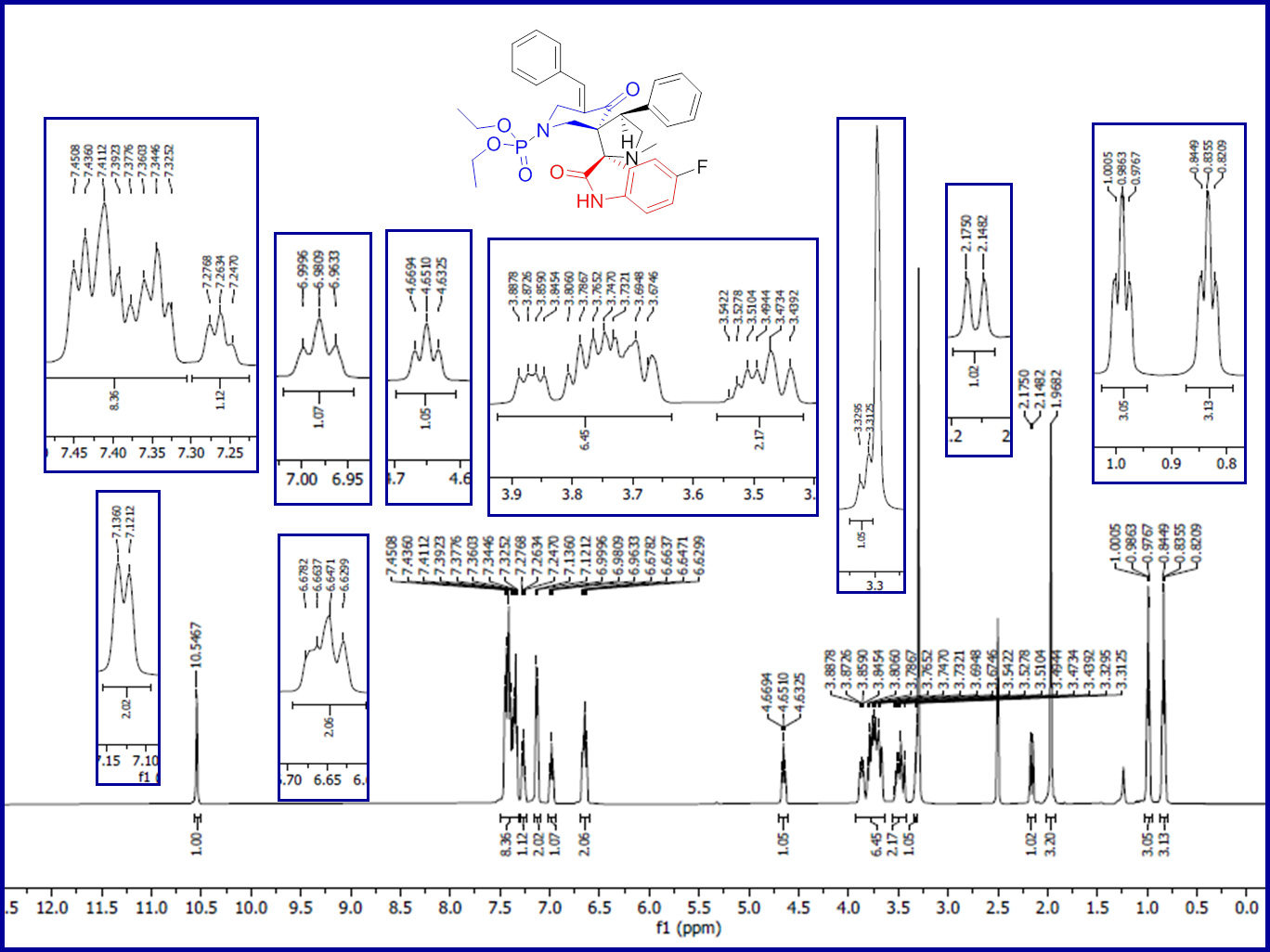


**Fig. S2.** ^1^H-NMR spectrum of compound **17b** in DMSO-*d6*.


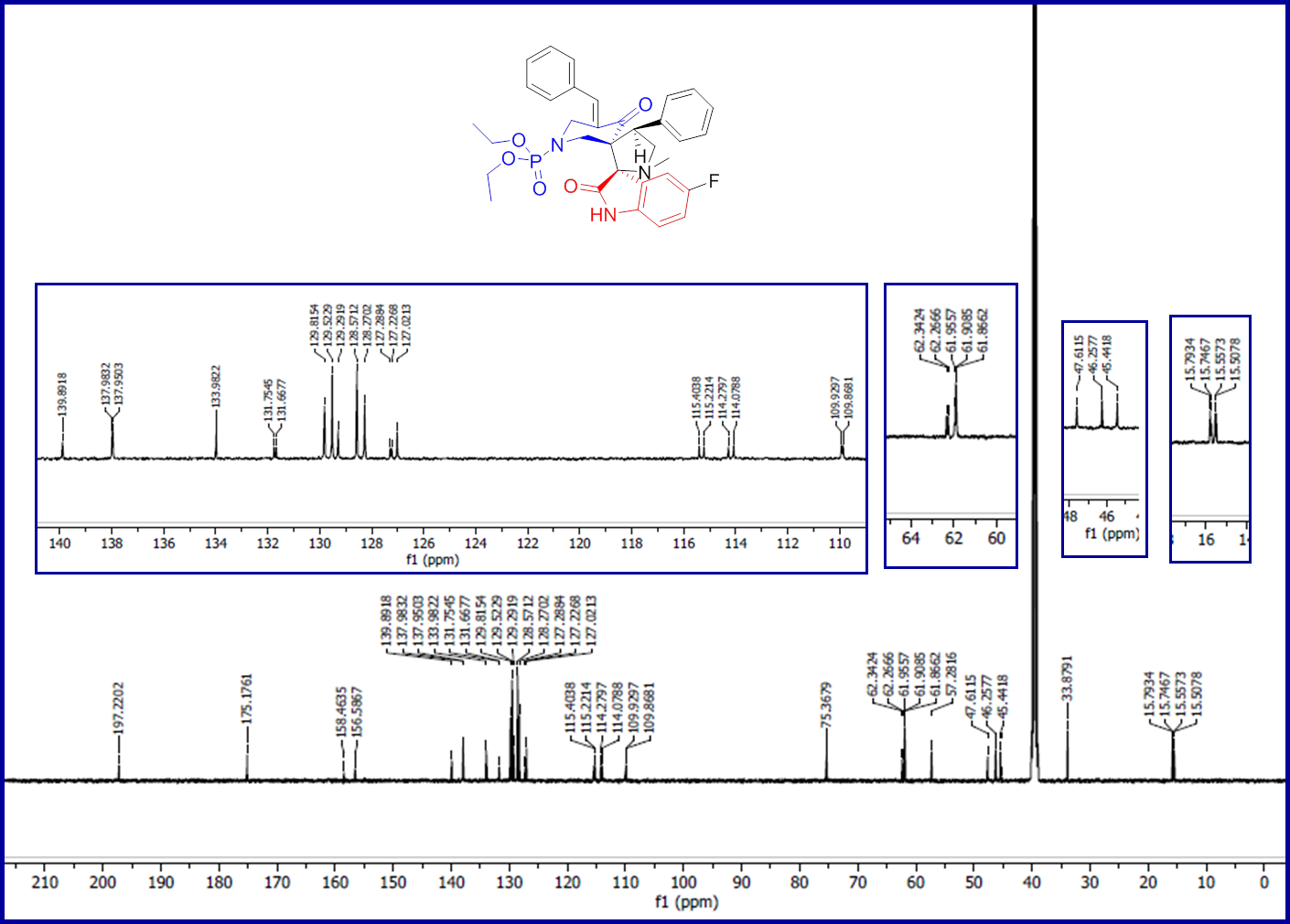


**Fig. S3.** ^13^C-NMR spectrum of compound **17b** in DMSO-*d6*.


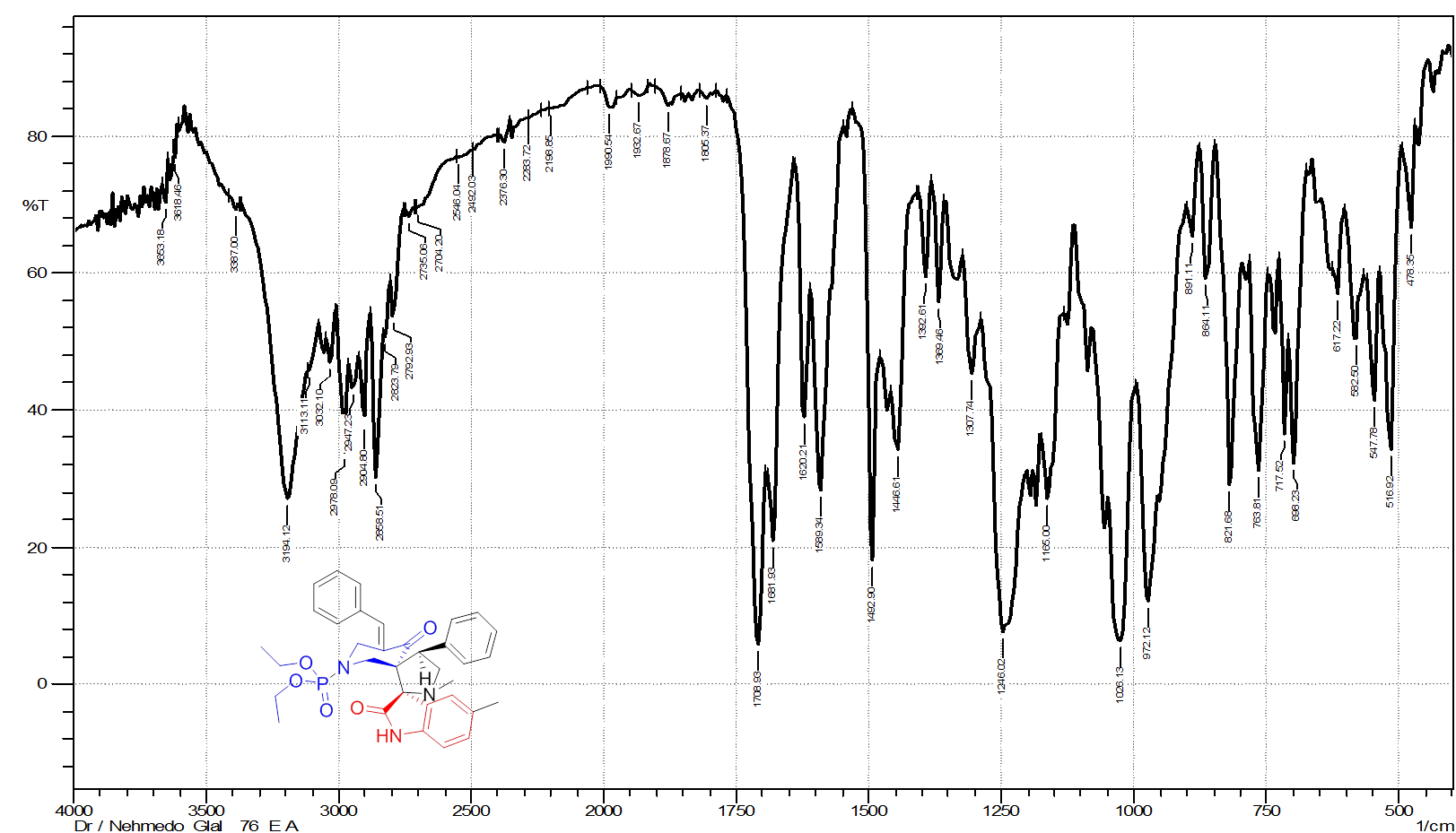


**Fig. S4.** IR spectrum of compound **17d** (KBr pellet).


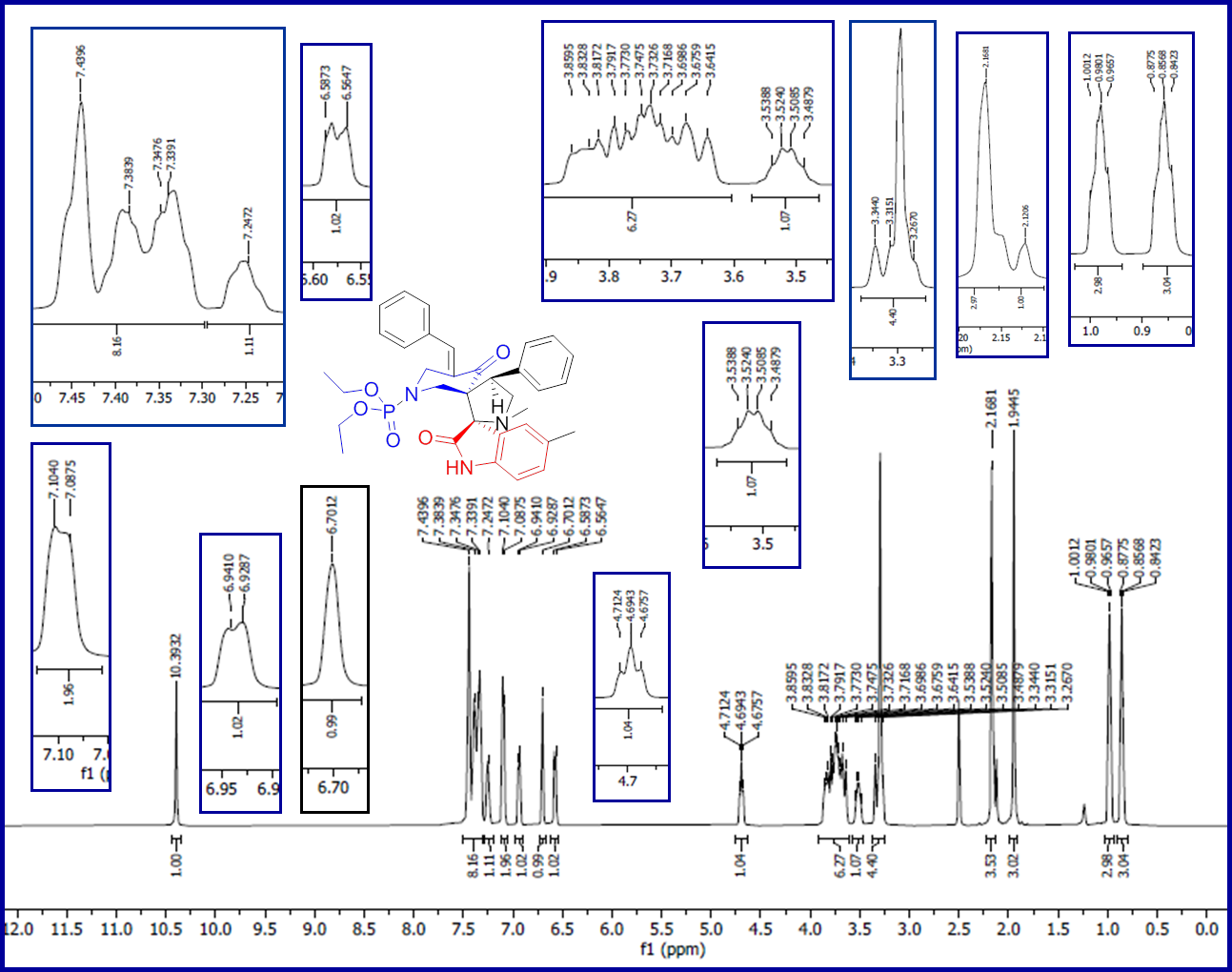


**Fig. S5.** ^1^H-NMR spectrum of compound **17d** in DMSO-*d6*.


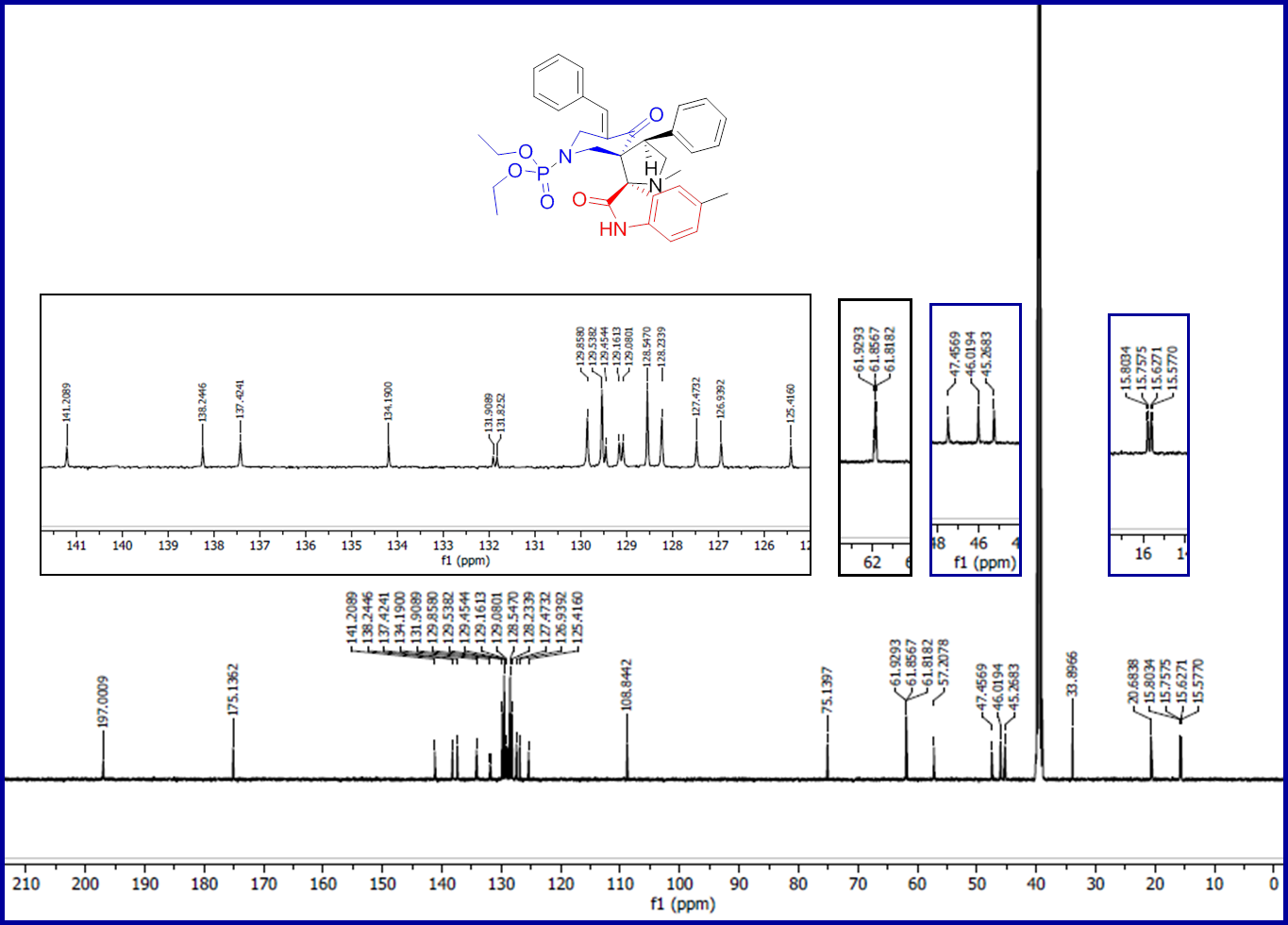


**Fig. S6.** ^13^C-NMR spectrum of compound **17d** in DMSO-*d6*.


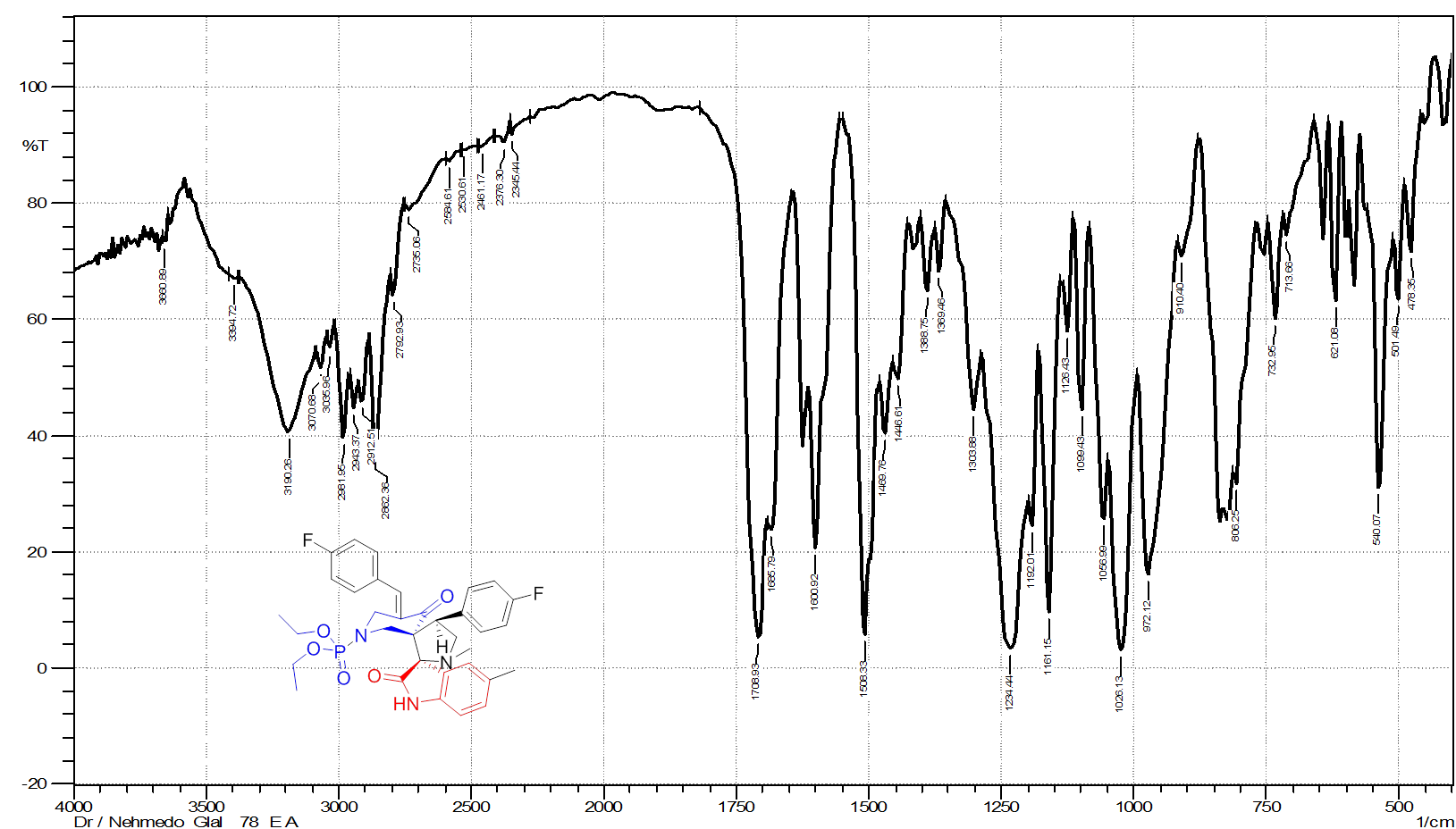


**Fig. S7.** IR spectrum of compound **17g** (KBr pellet).


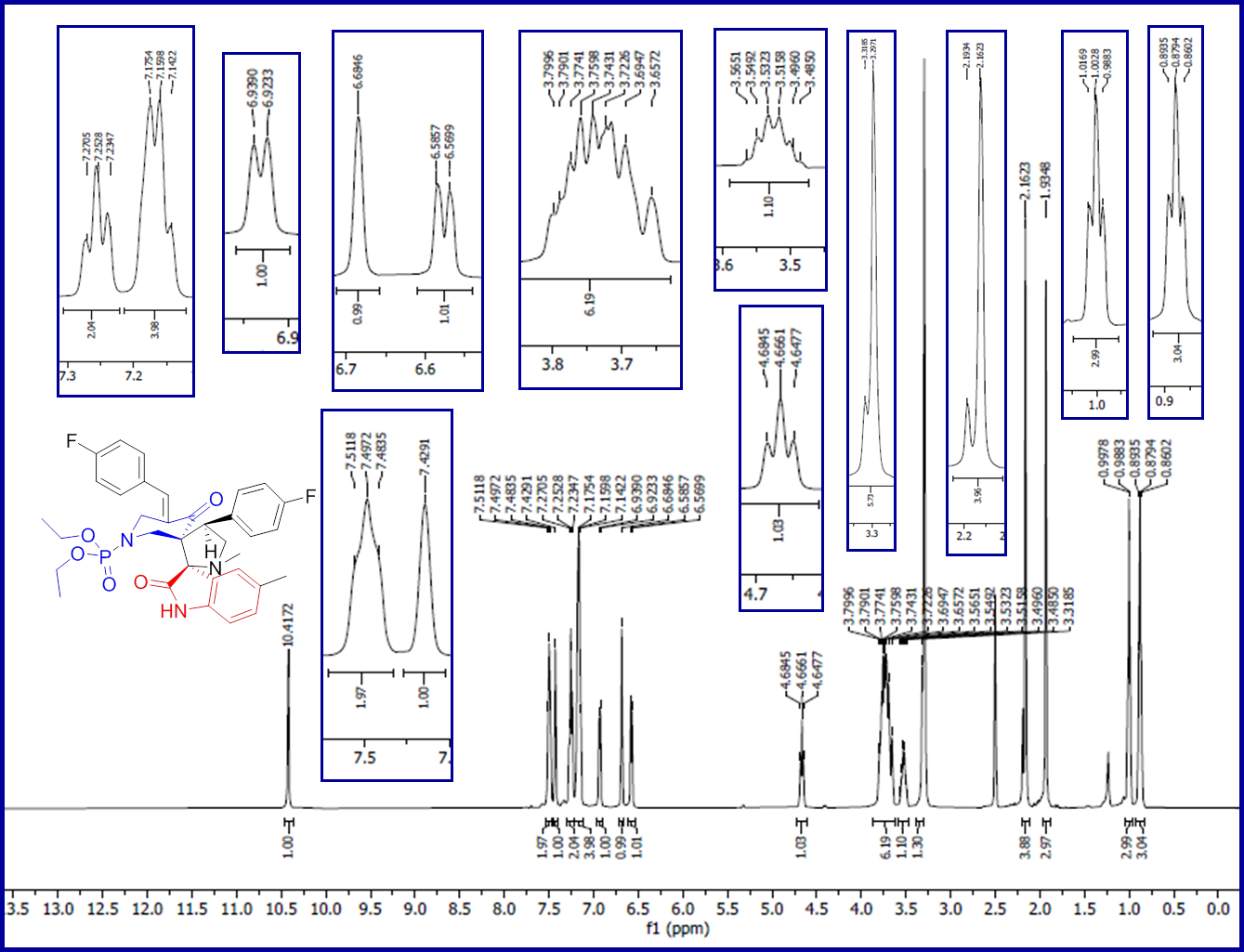


**Fig. S8.** ^1^H-NMR spectrum of compound **17g** in DMSO-*d6*.


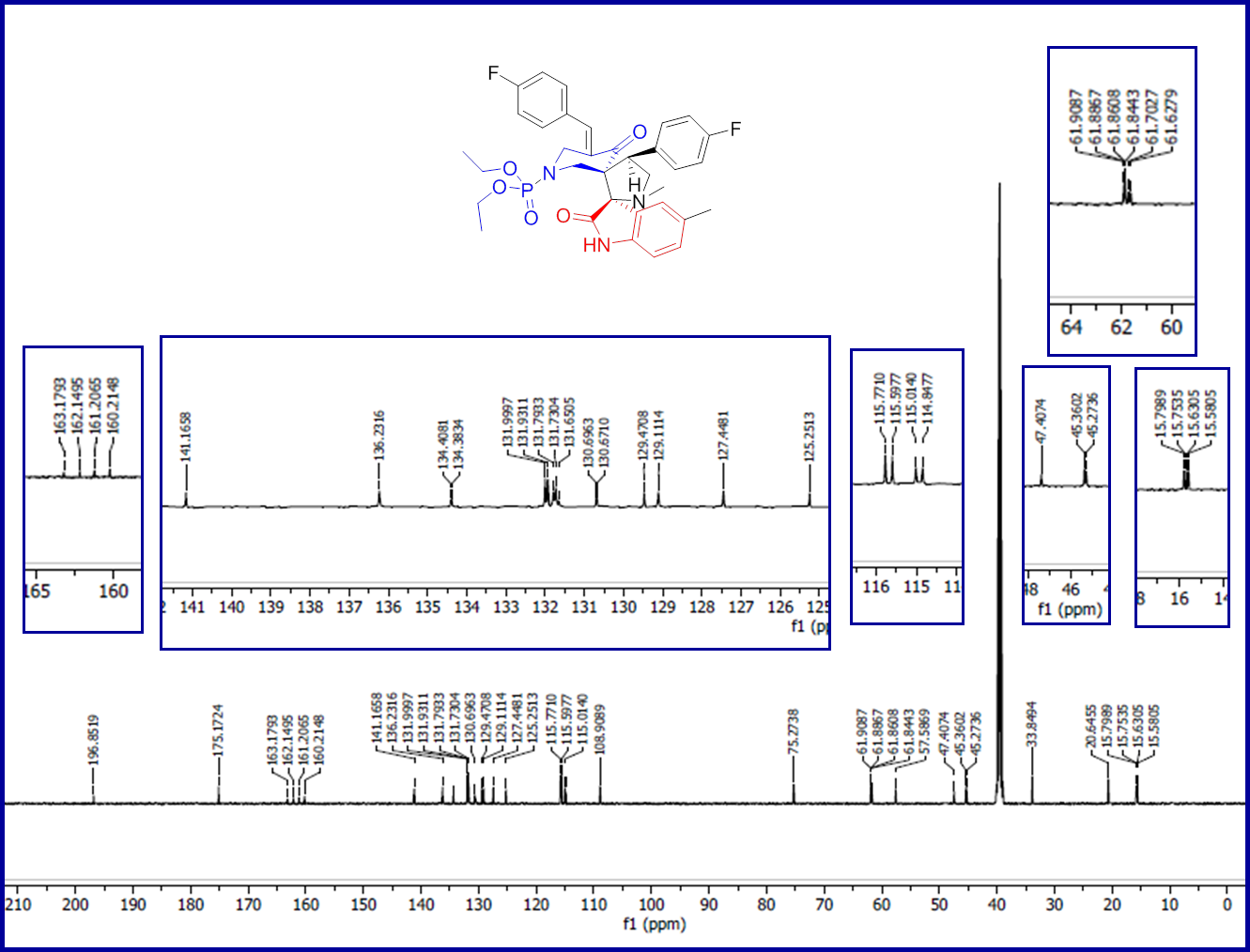


**Fig. S9.** ^13^C-NMR spectrum of compound **17g** in DMSO-*d6*.


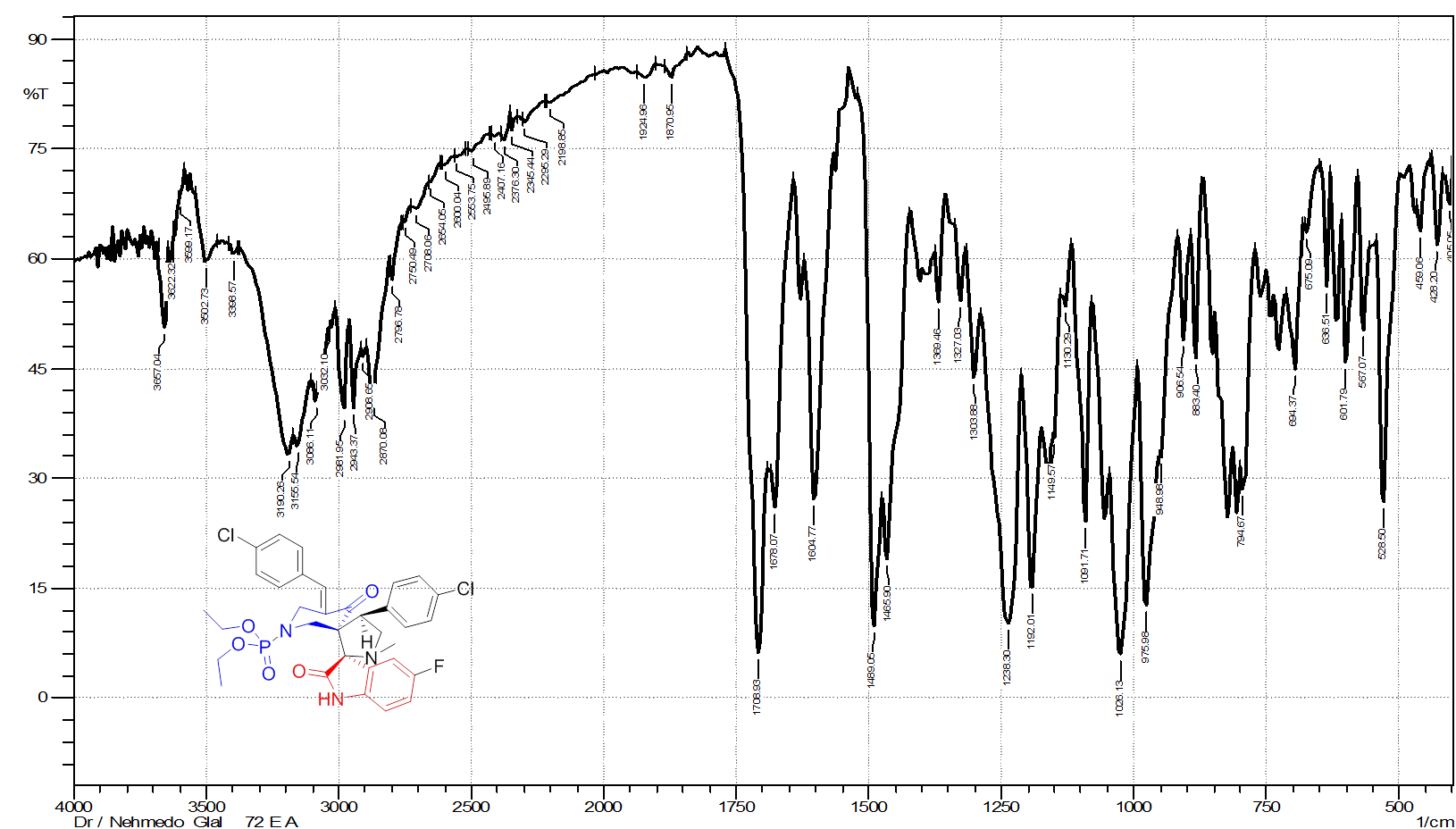


**Fig. S10.** IR spectrum of compound **17i** (KBr pellet).


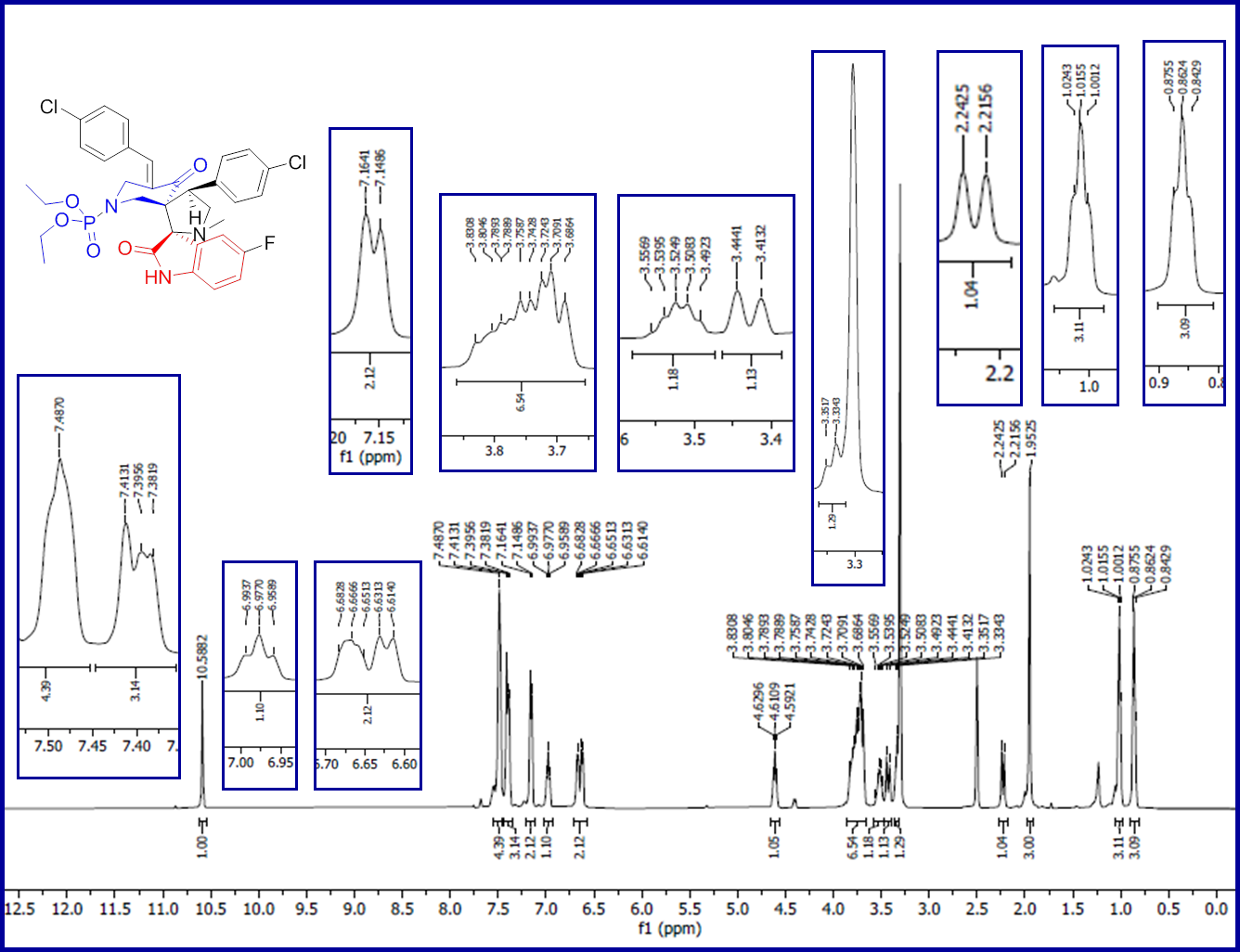


**Fig. S11.** ^1^H-NMR spectrum of compound **17i** in DMSO-*d6*.


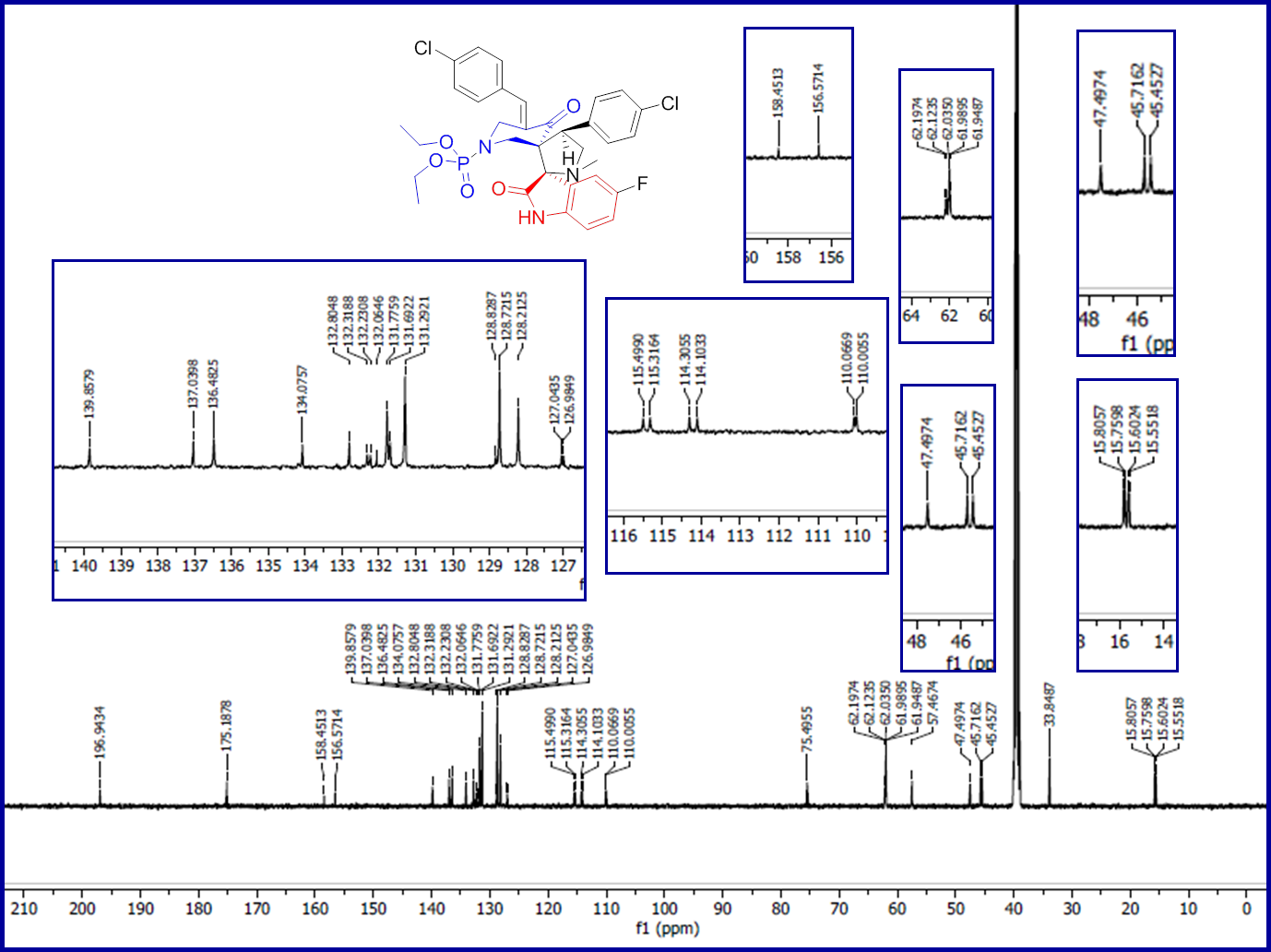


**Fig. S12.** ^13^C-NMR spectrum of compound **17i** in DMSO-*d6*.


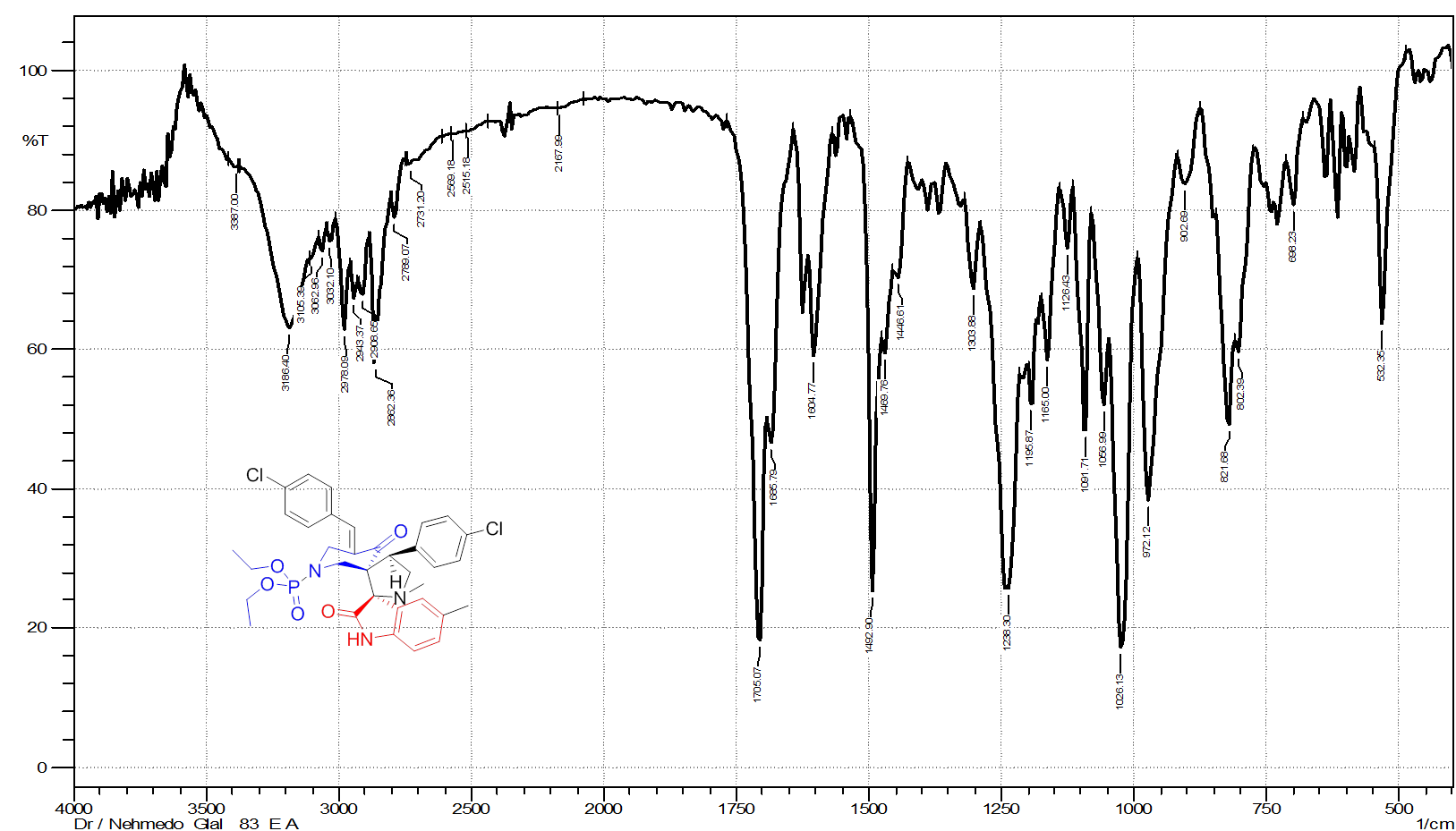


**Fig. S13.** IR spectrum of compound **17k** (KBr pellet).


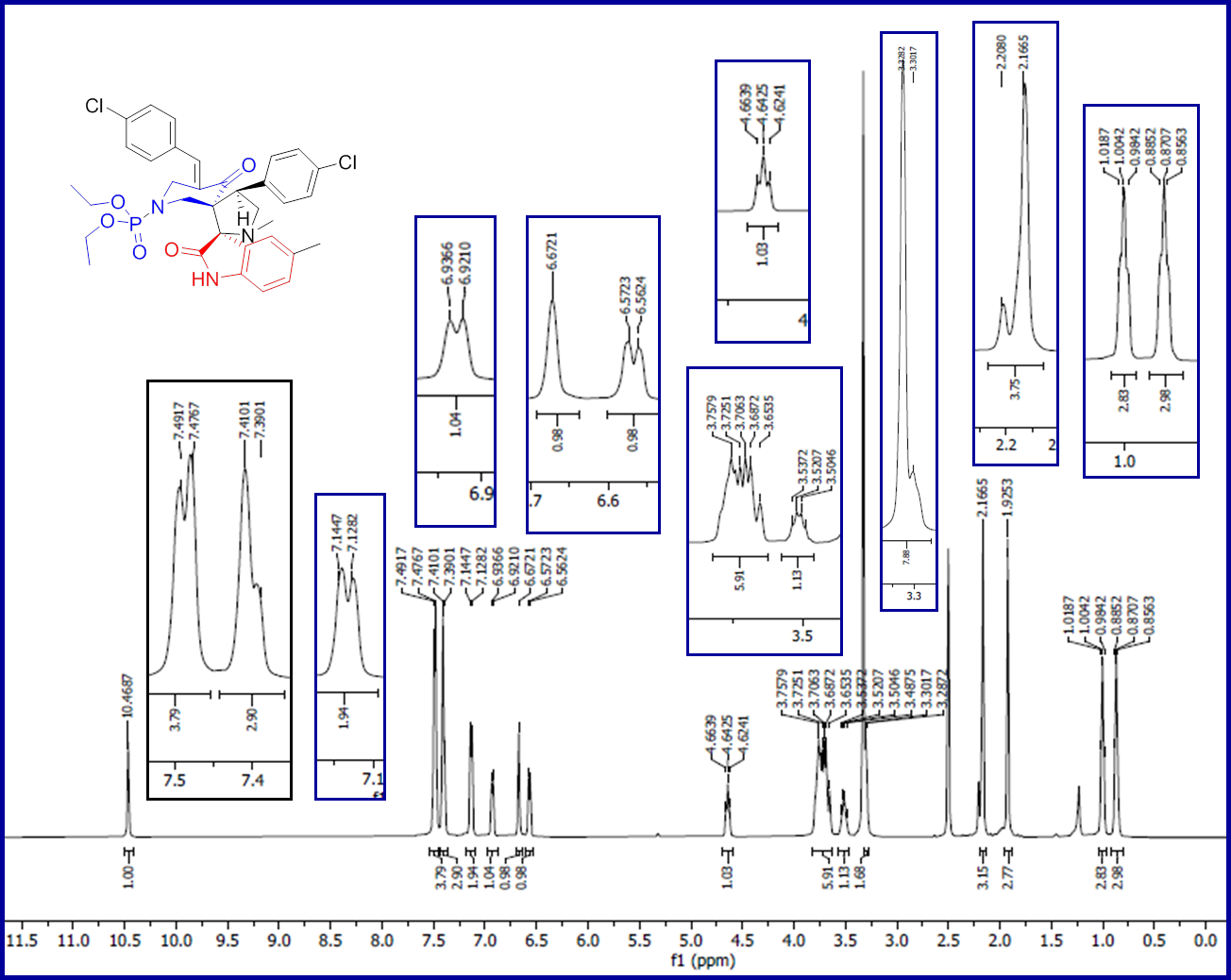


**Fig. S14.** ^1^H-NMR spectrum of compound **17k** in DMSO-*d6*.


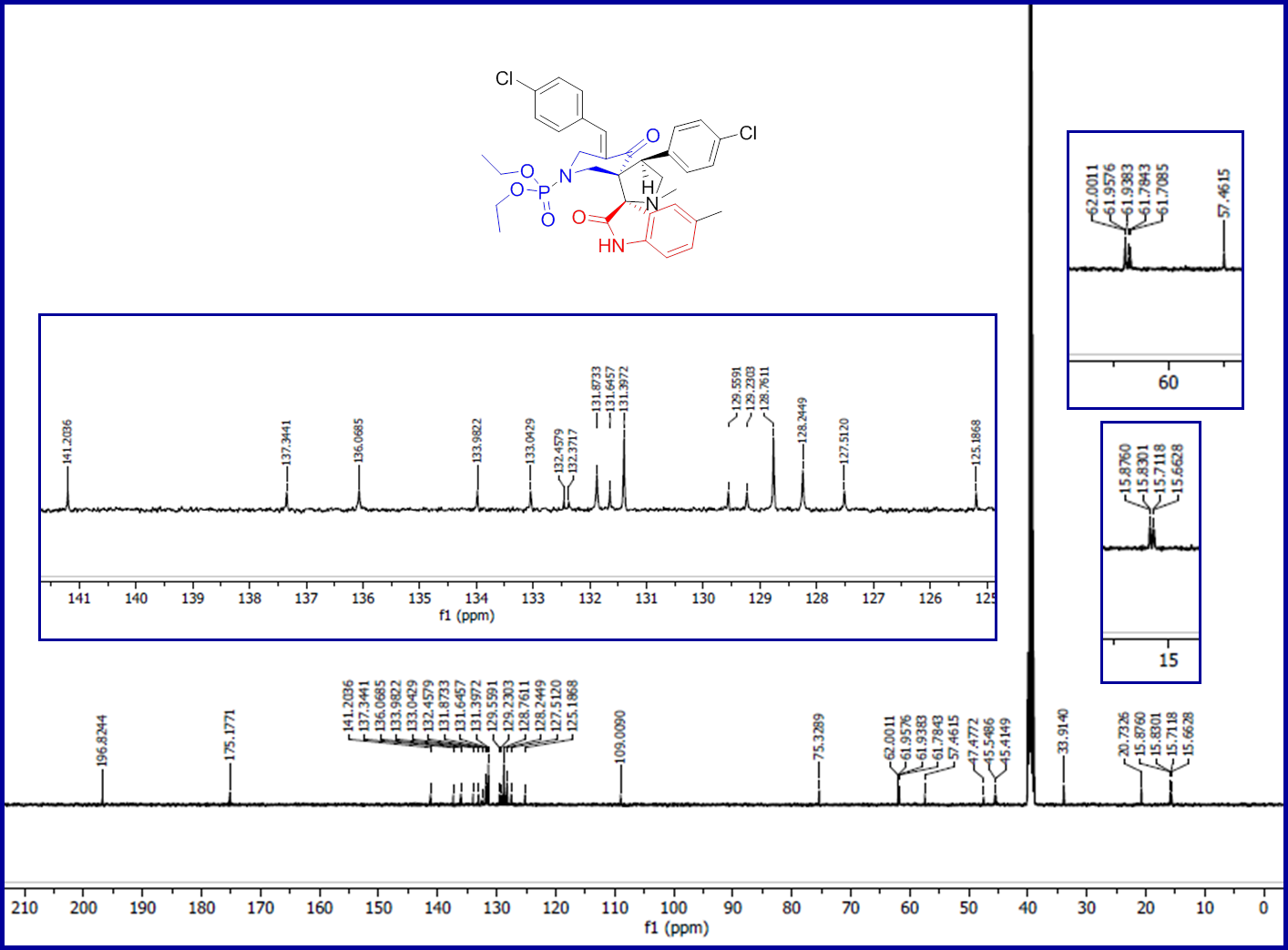


**Fig. S15.** ^13^C-NMR spectrum of compound **17k** in DMSO-*d6*.


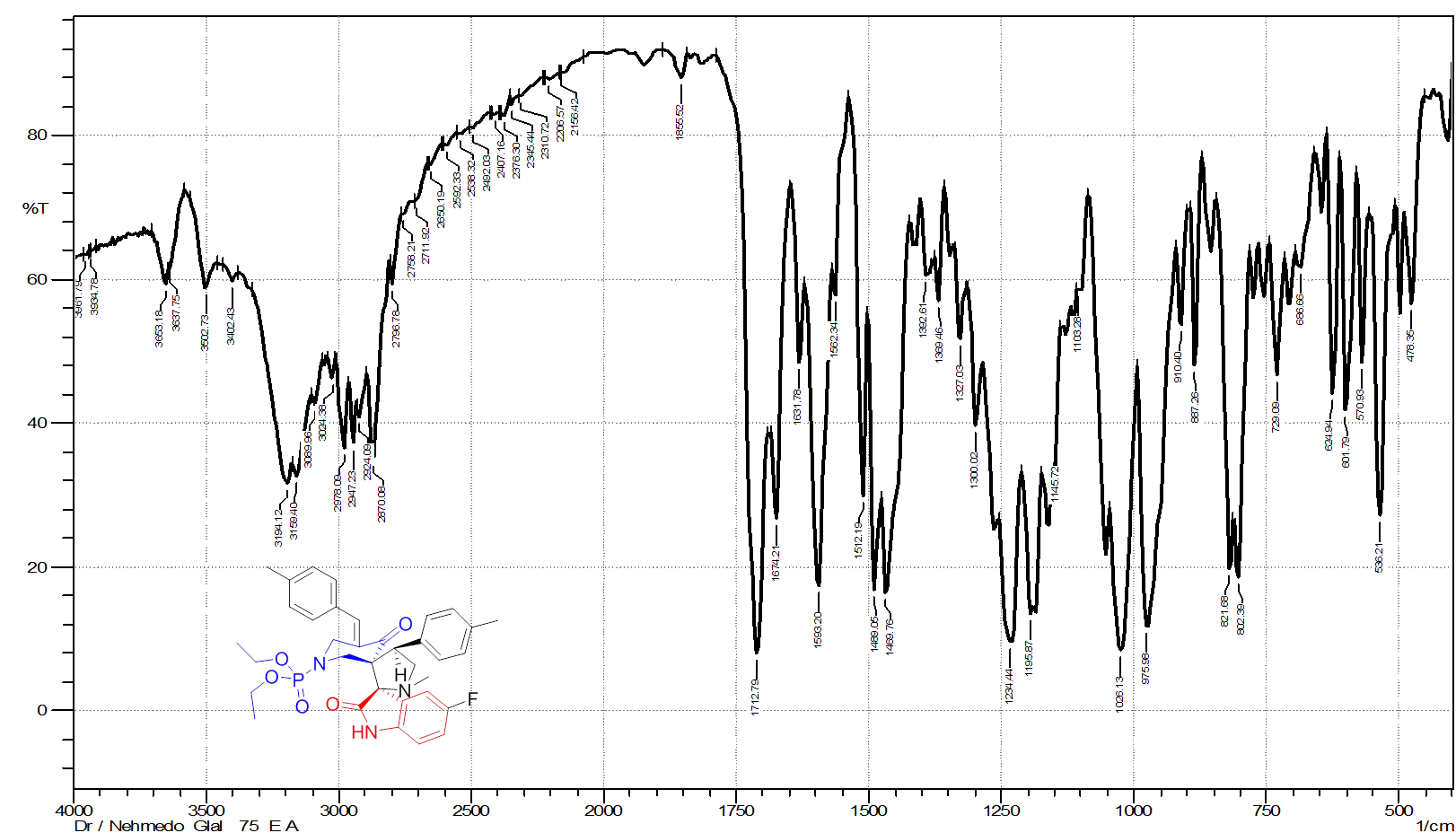


**Fig. S16.** IR spectrum of compound **17n** (KBr pellet).


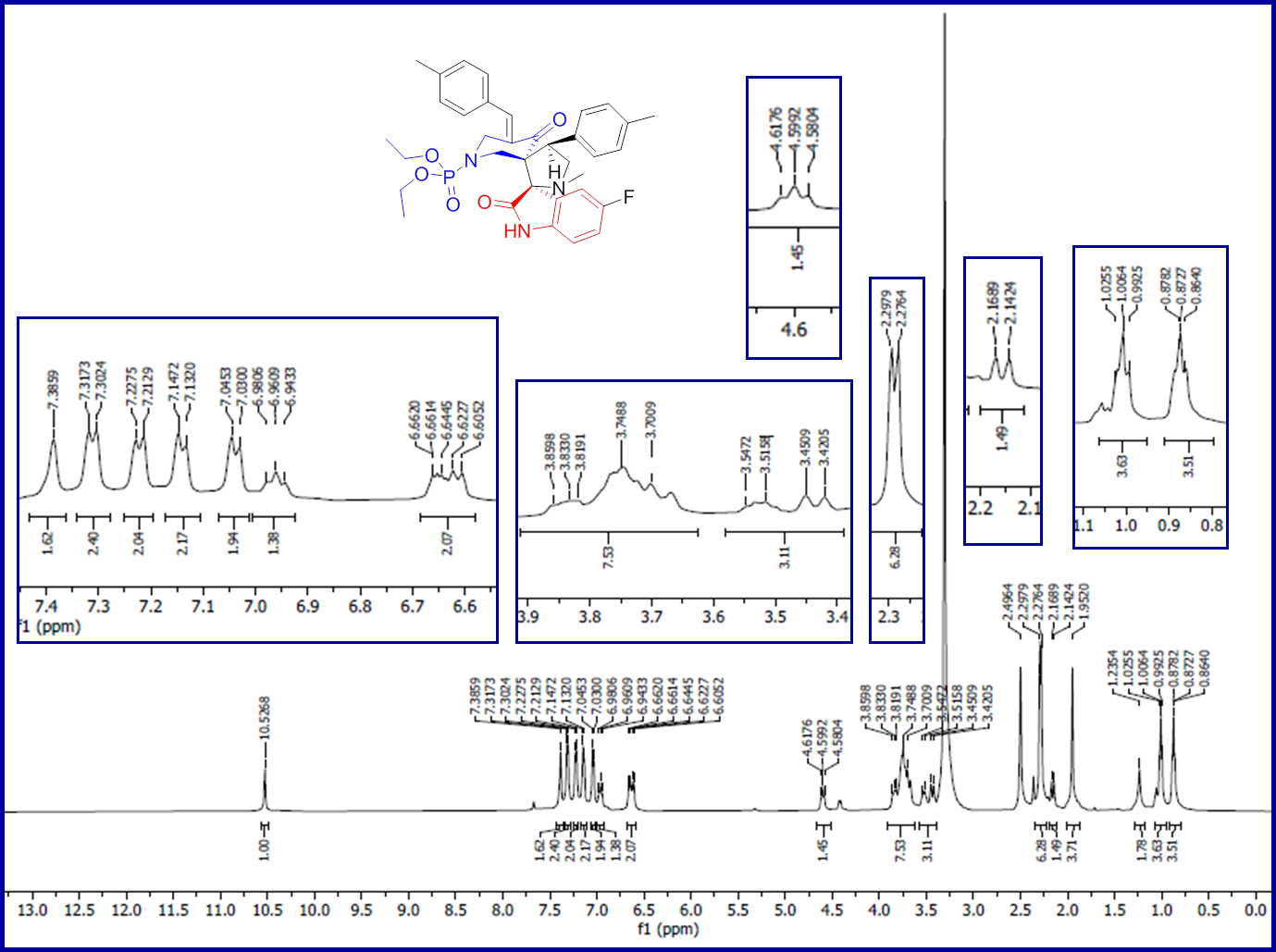


**Fig. S17.** ^1^H-NMR spectrum of compound **17n** in DMSO-*d6*.


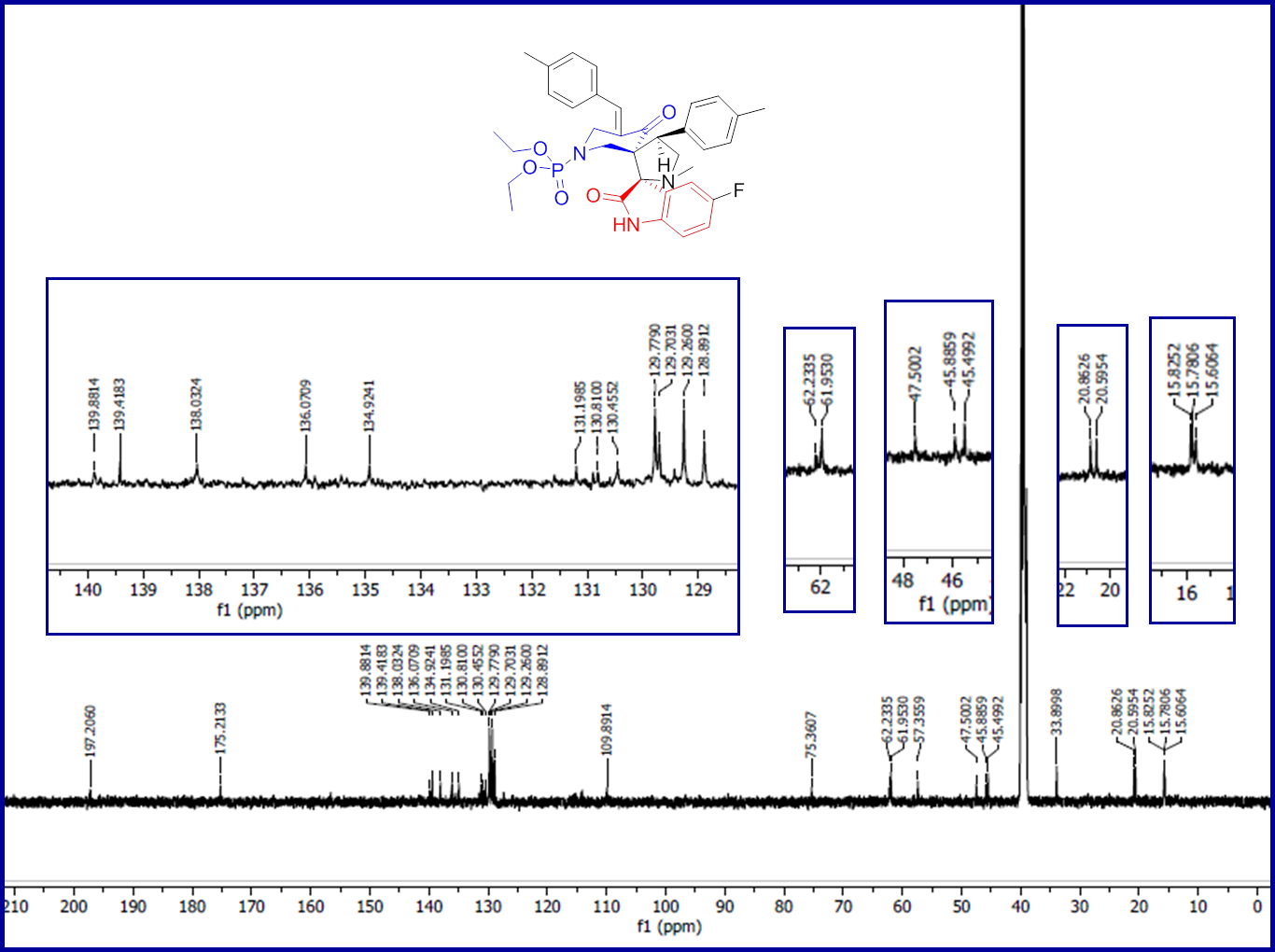


**Fig. S18.** ^13^C-NMR spectrum of compound **17n** in DMSO-*d6*.


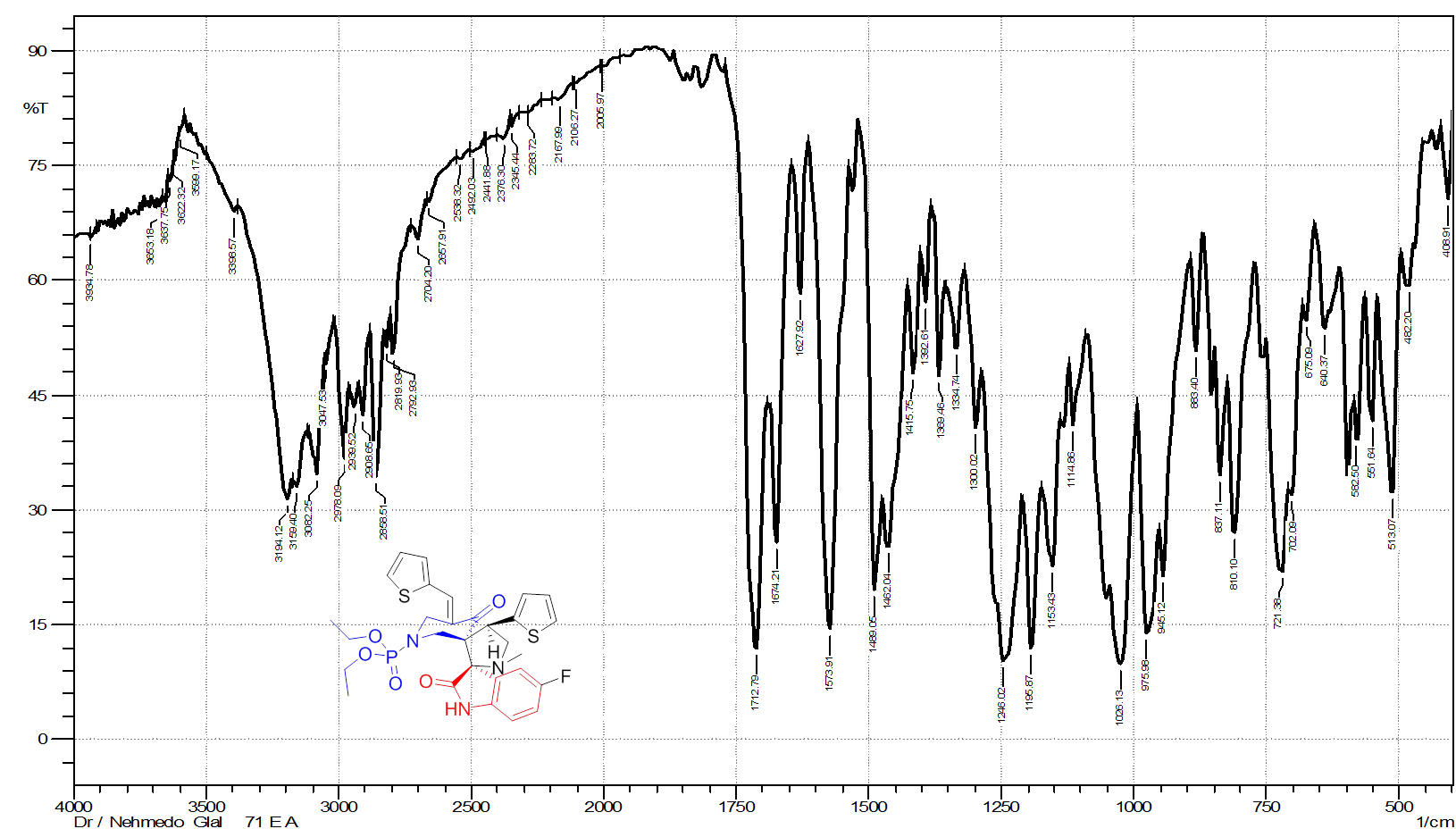


**Fig. S19.** IR spectrum of compound **17r** (KBr pellet).


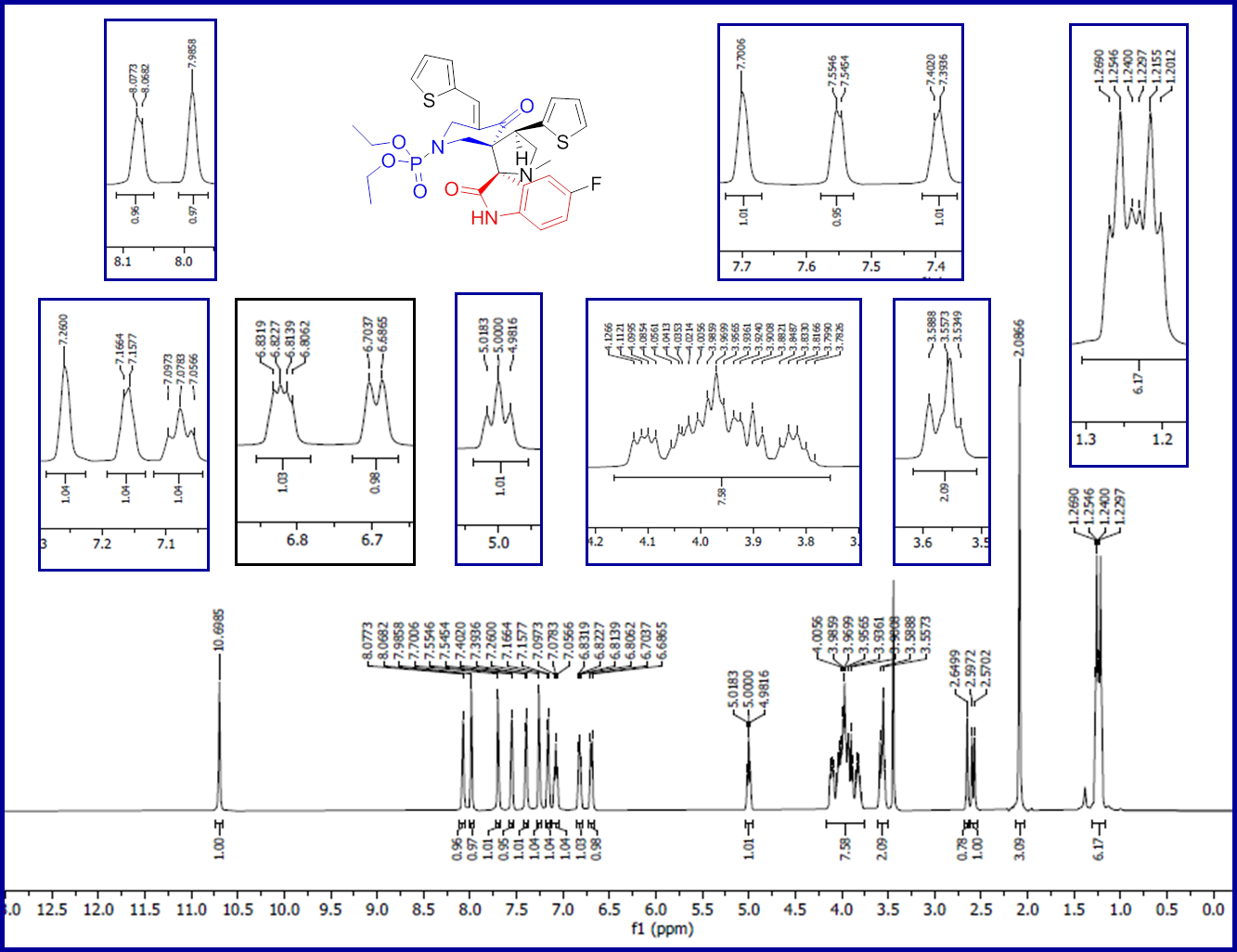


**Fig. S20.** ^1^H-NMR spectrum of compound **17r** in DMSO-*d6*.


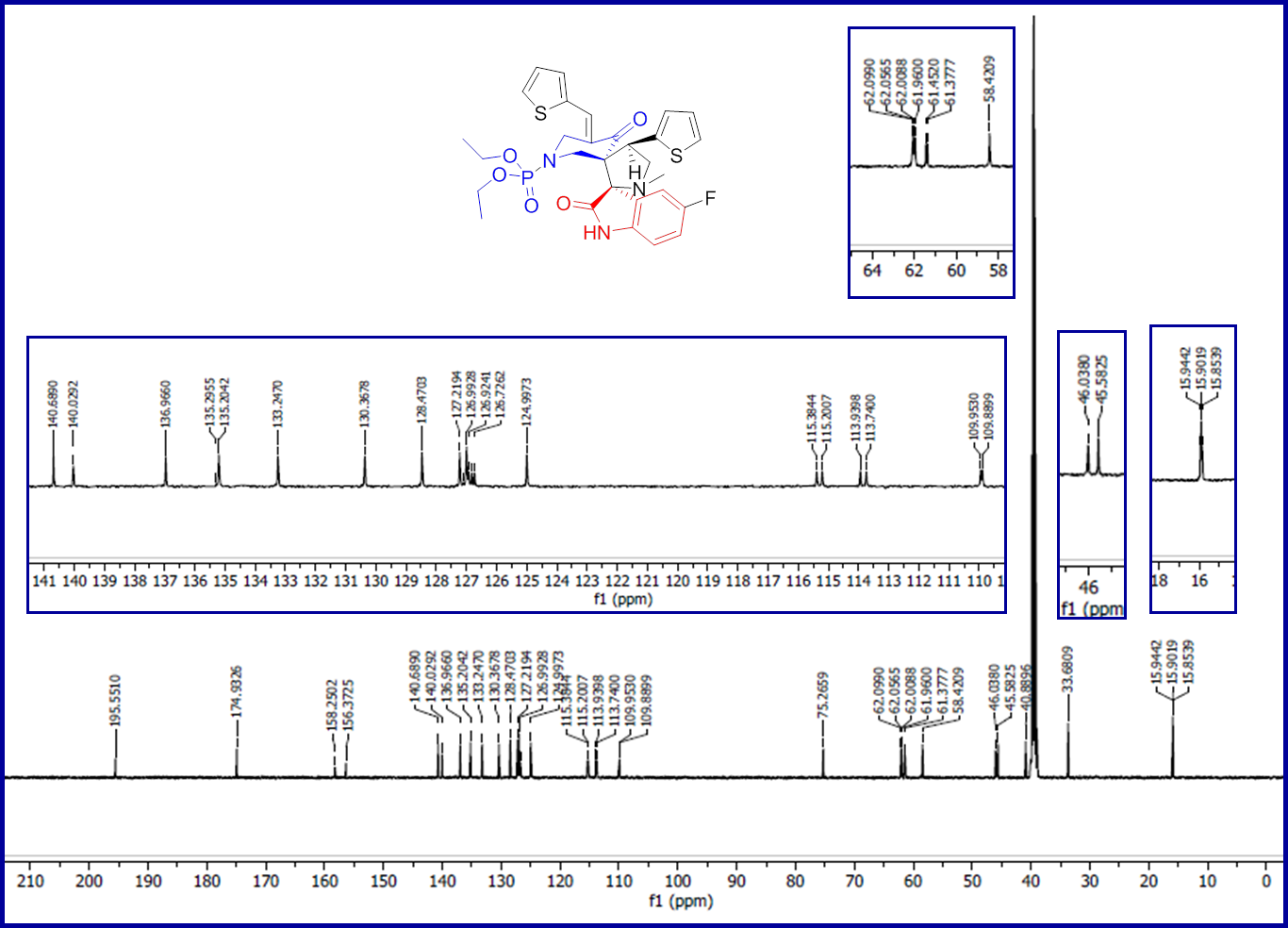


**Fig. S21.** ^13^C-NMR spectrum of compound **17r** in DMSO-*d6*.


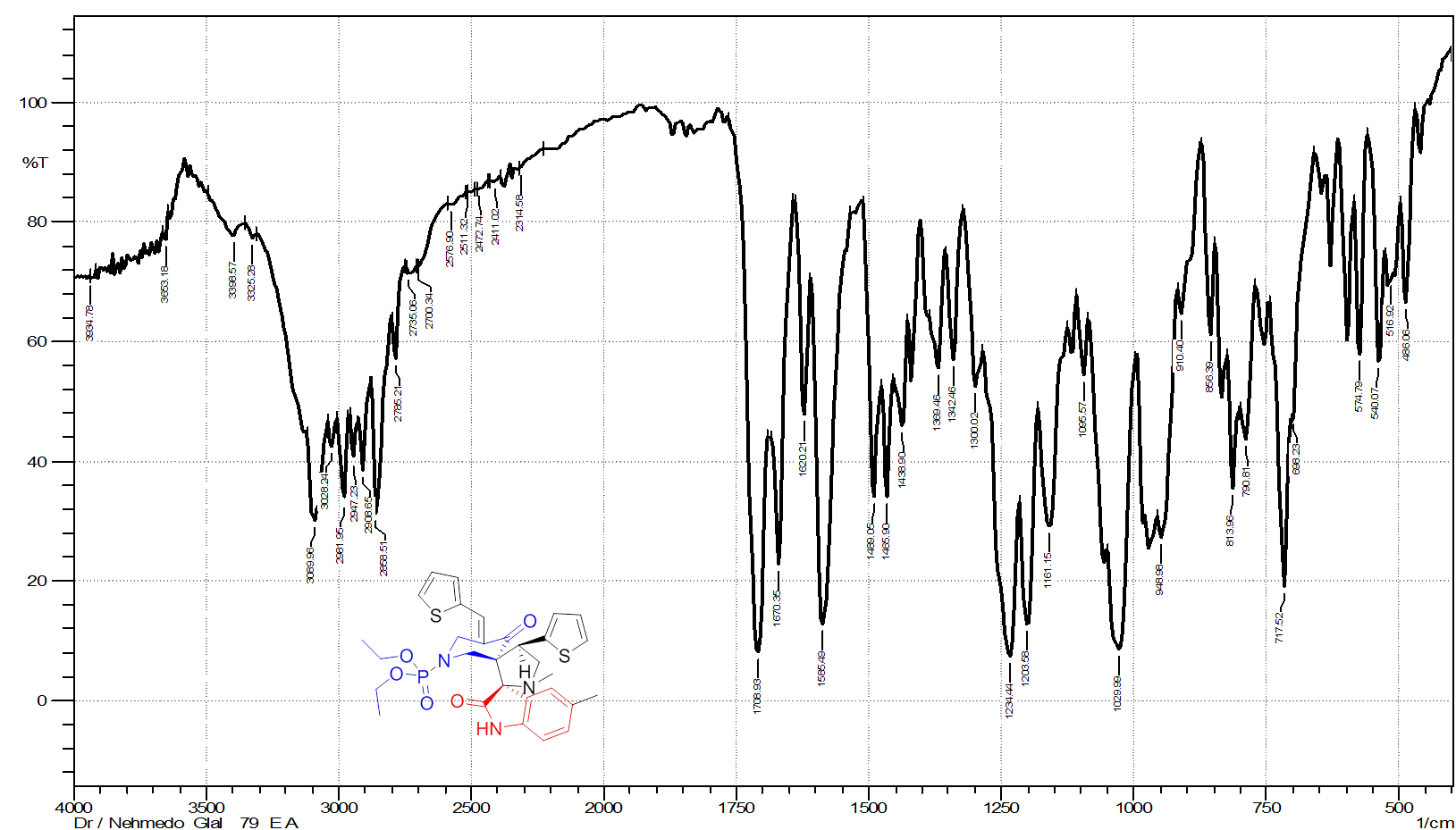


**Fig. S22.** IR spectrum of compound **17t** (KBr pellet).


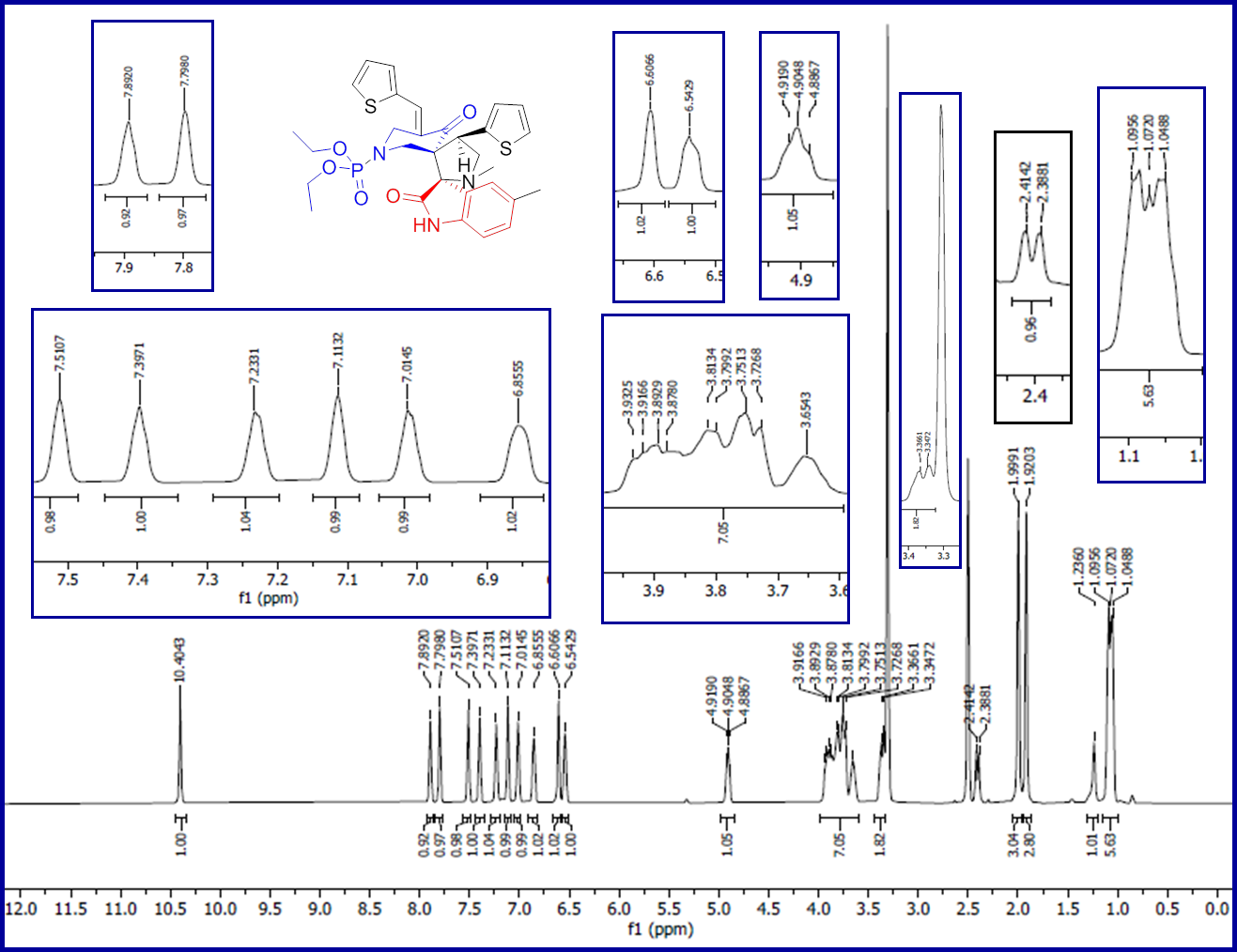


**Fig. S23.** ^1^H-NMR spectrum of compound **17t** in DMSO-*d6*.


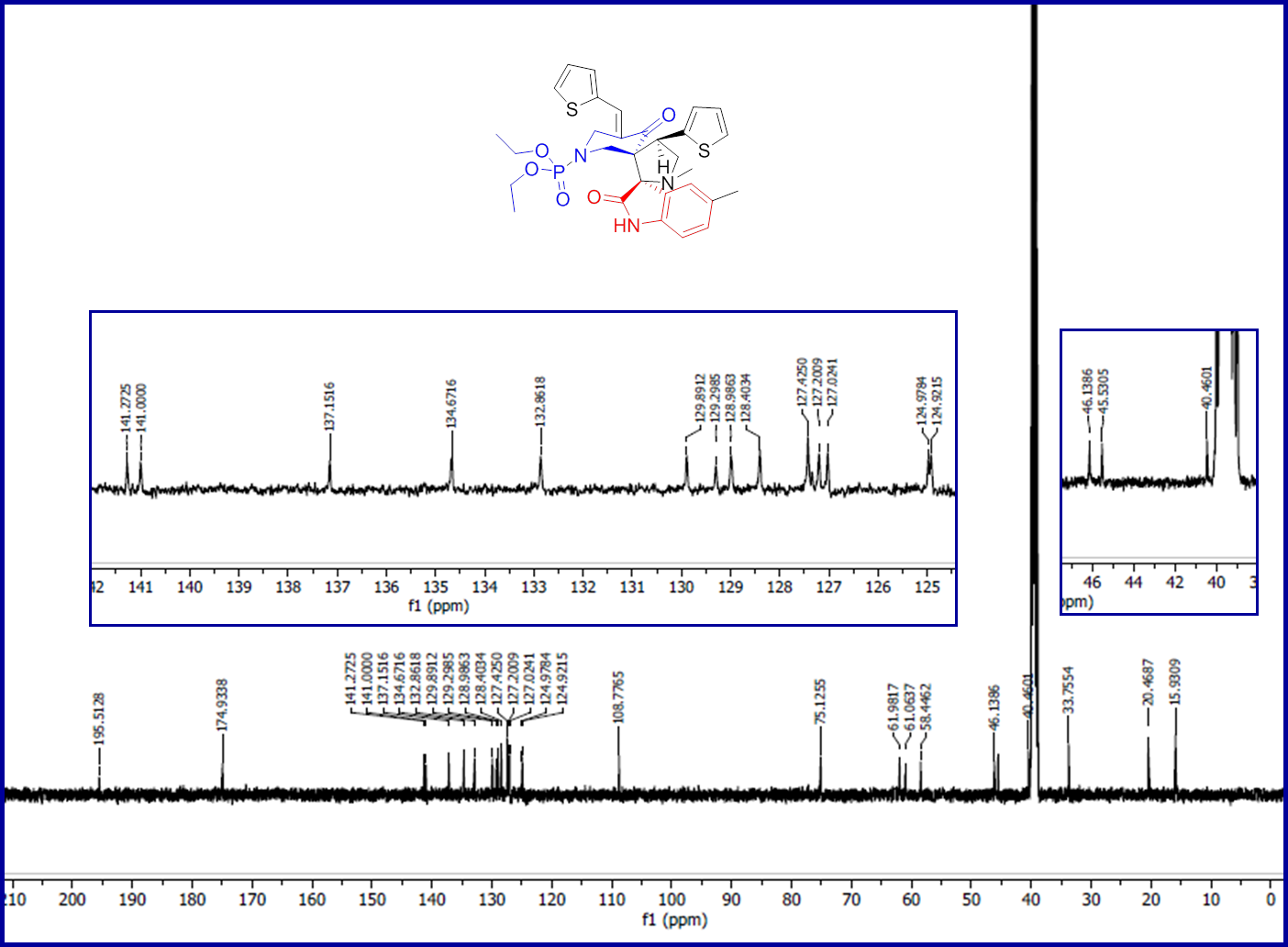


**Fig. S24.** ^13^C-NMR spectrum of compound **17t** in DMSO-*d6*.


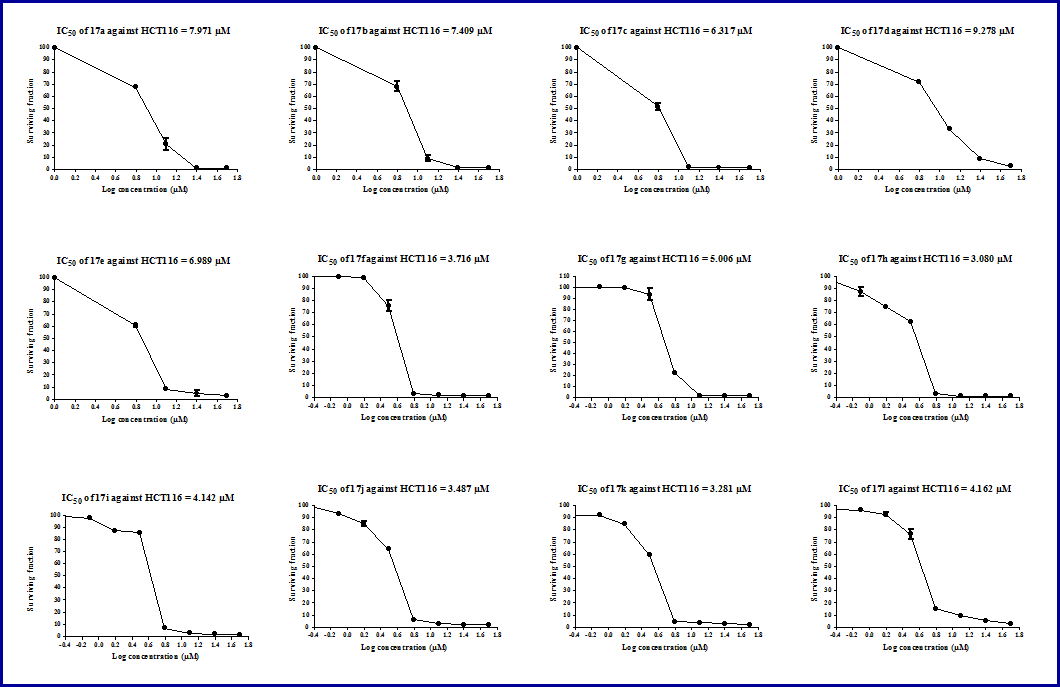


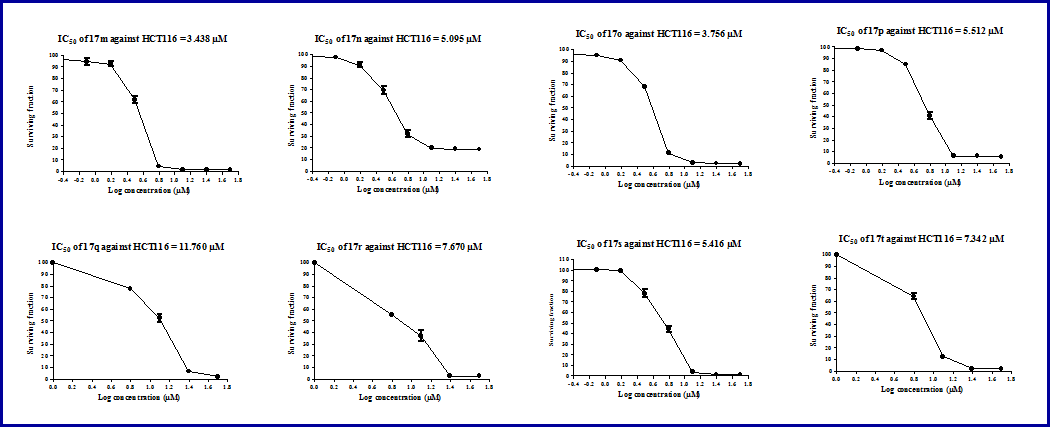


**Fig. S25.** Dose response curves of **17a‒t** against HCT116 (colon) cancer cell line.


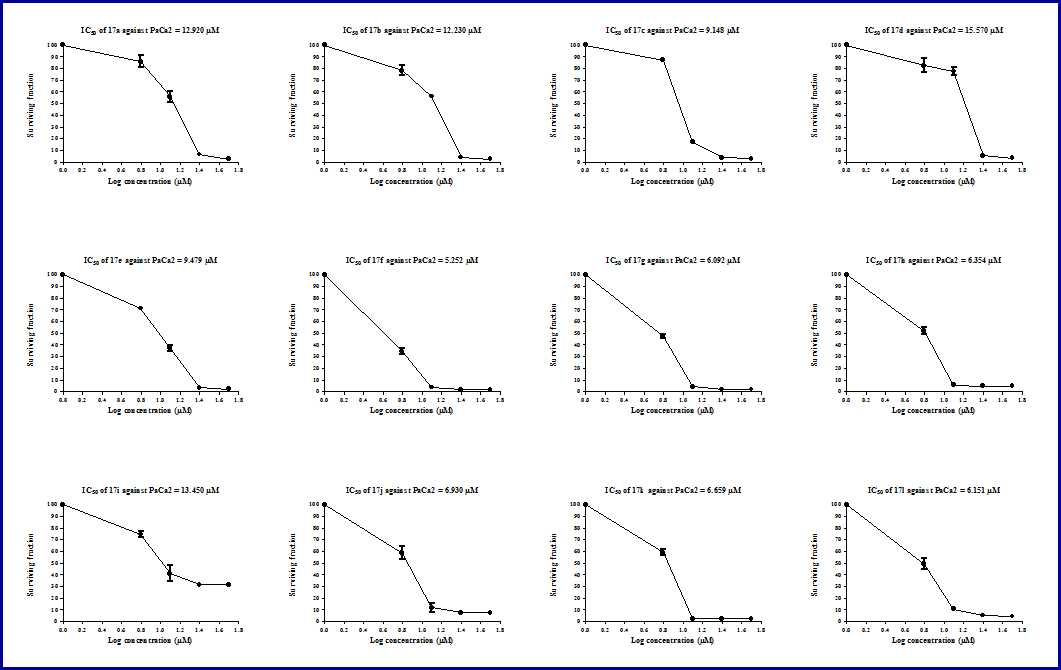


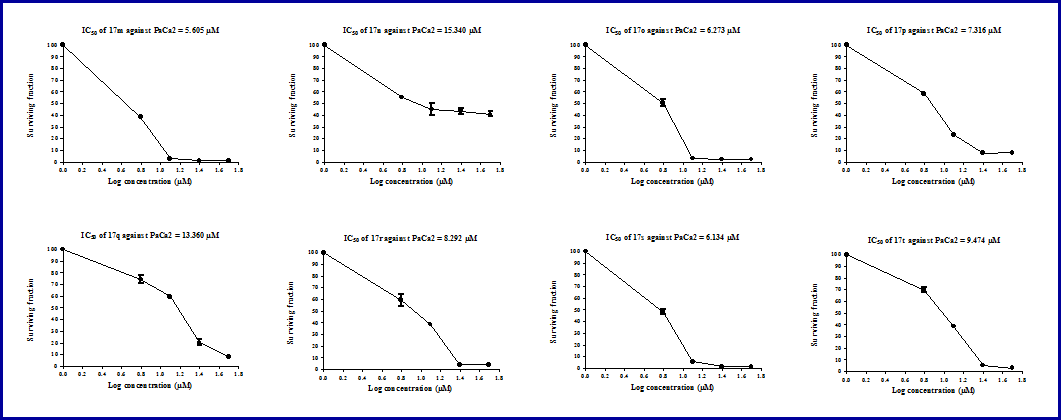


**Fig. S26.** Dose response curves of **17a‒t** against PaCa2 (pancreatic) cancer cell line.


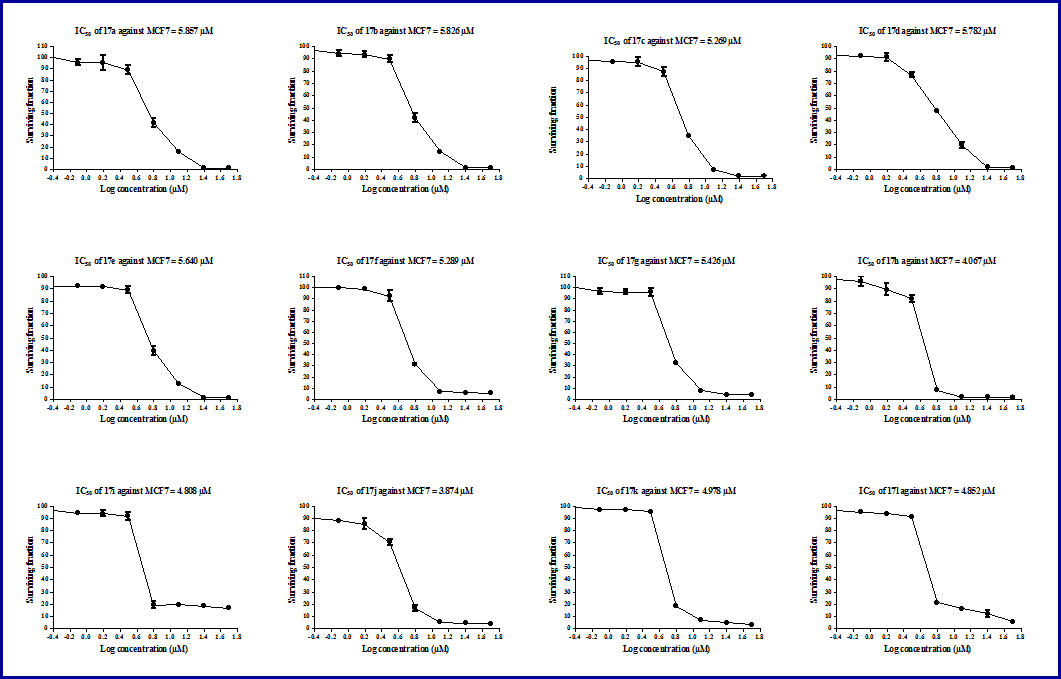


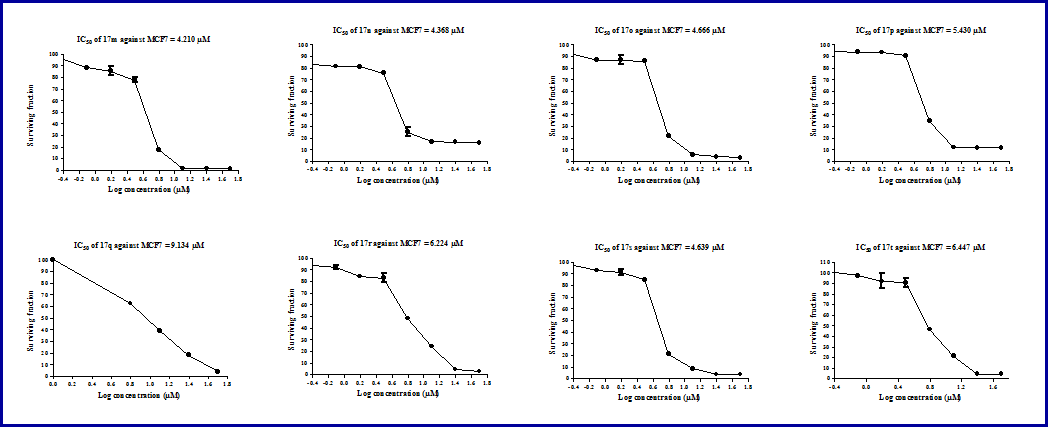


**Fig. S27.** Dose response curves of **17a‒t** against MCF7 (breast) cancer cell line.


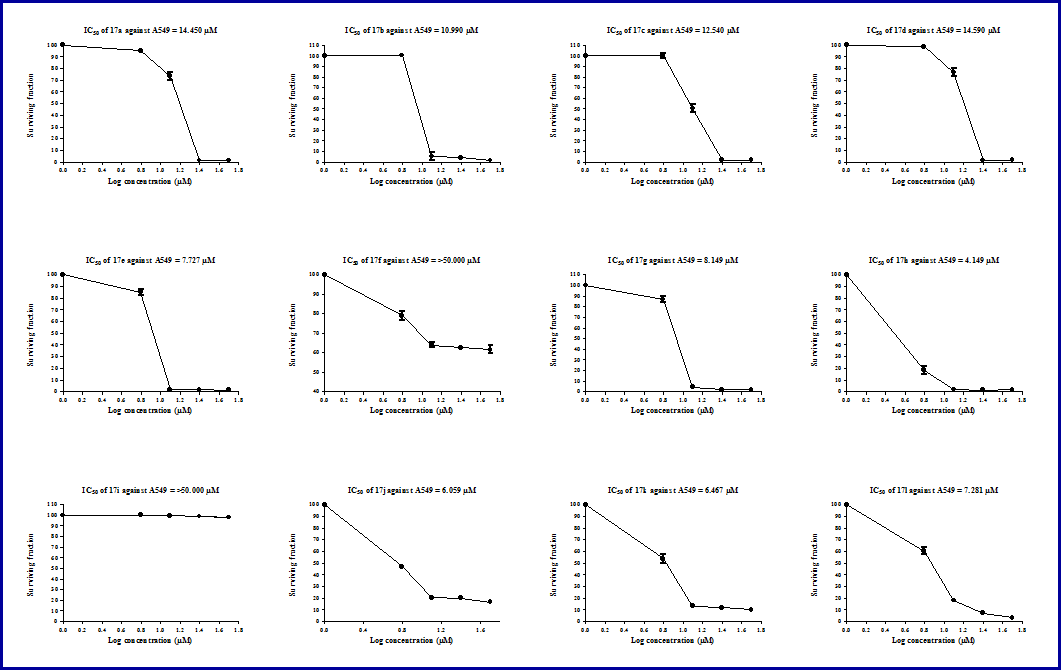


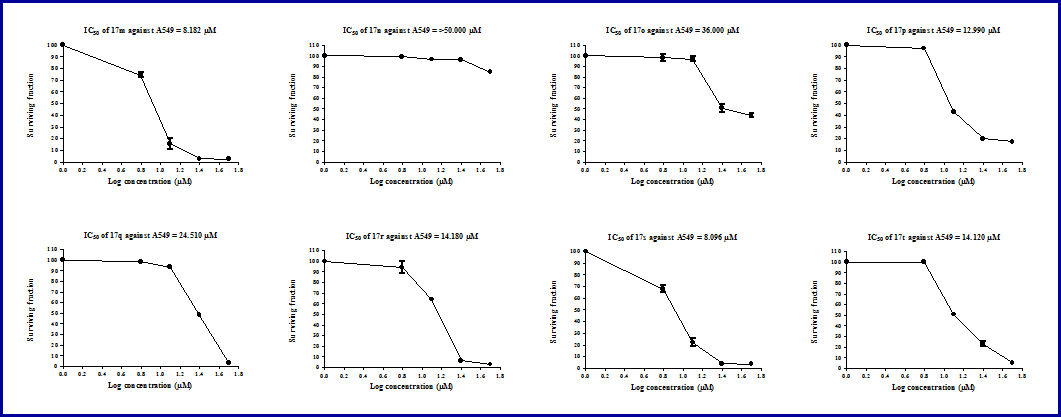


**Fig. S28.** Dose response curves of **17a‒t** against A549 (lung) cancer cell line.


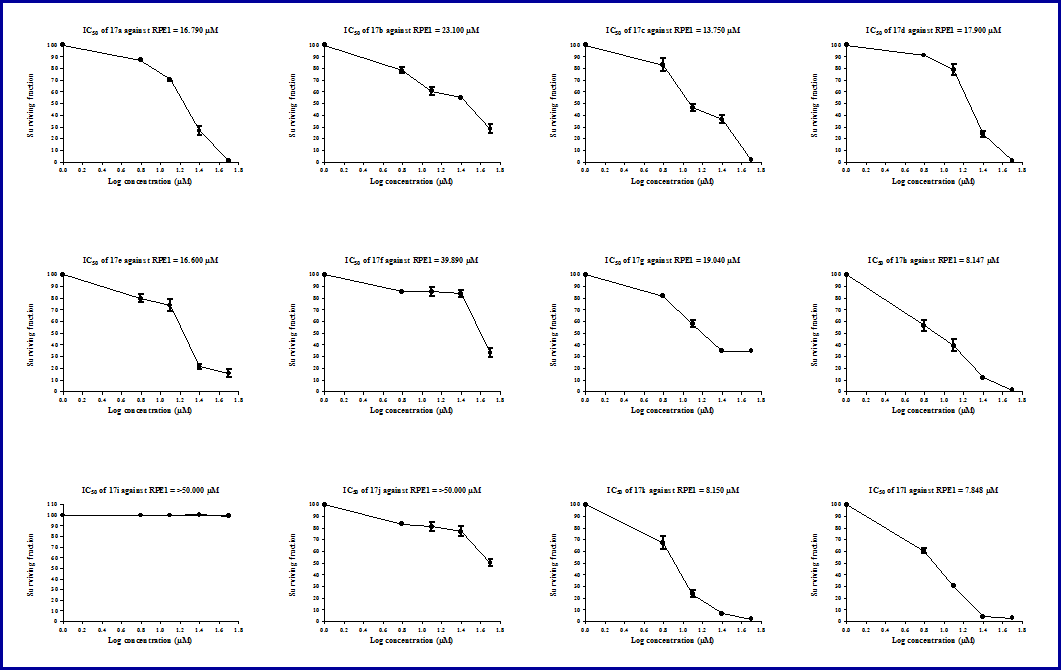


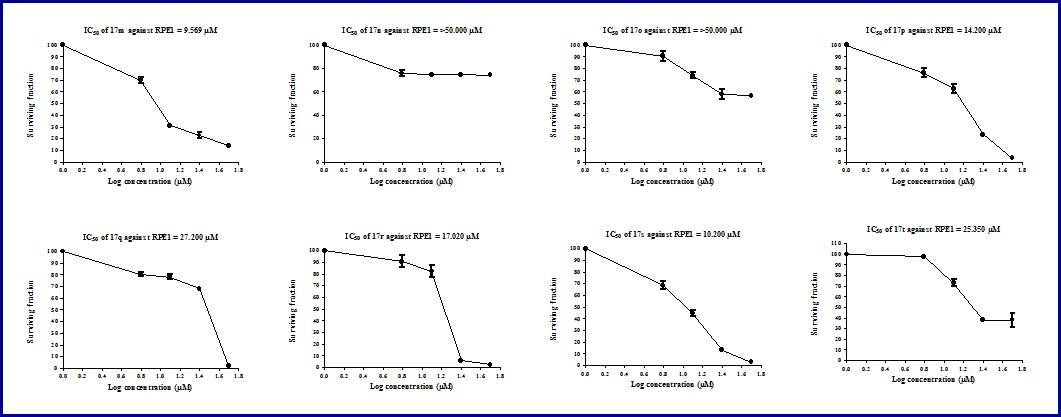


**Fig. S29.** Dose response curves of **17a‒t** against RPE1 normal/non-cancer cell line.


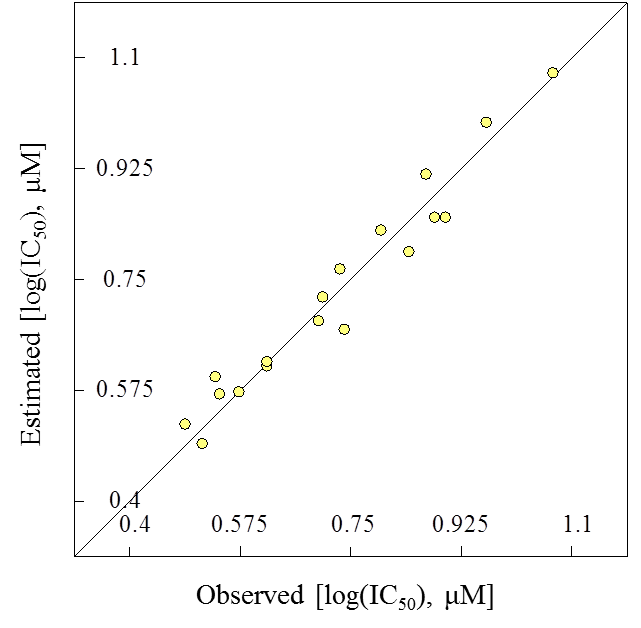


**Fig. S30.** QSAR plot representing the observed versus predicted training set compounds of anti-HCT116 properties [log(IC_50_) μM).


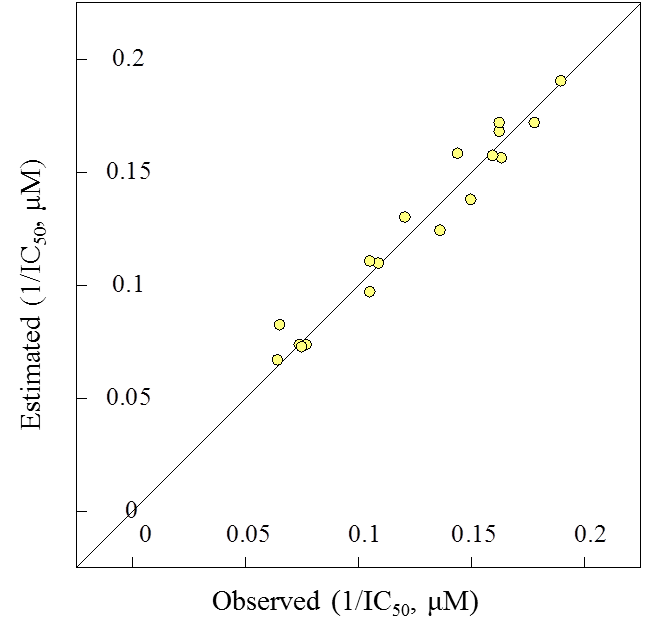


**Fig. S31.** QSAR plot representing the observed versus predicted training set compounds of anti-PaCa2 properties (1/IC_50_, μM).
